# Endorsements vs. information: Experimental evidence of backlash and parallel persuasion during the COVID-19 public health crisis

**DOI:** 10.1093/pnasnexus/pgaf185

**Published:** 2025-06-05

**Authors:** Ryan Baxter-King, Alexander Coppock, Graham Straus, Lynn Vavreck

**Affiliations:** Political Science, University of California, Los Angeles, 4289 Bunche Hall, Los Angeles, CA 90095, USA; Political Science, Yale University, 115 Prospect St, New Haven, CT 06511, USA; Political Science, University of California, Los Angeles, 4289 Bunche Hall, Los Angeles, CA 90095, USA; Political Science, University of California, Los Angeles, 4289 Bunche Hall, Los Angeles, CA 90095, USA

**Keywords:** COVID-19, endorsements, guidance and information, persuasion, survey experiments

## Abstract

Governments try to promote prosocial behaviors like gun safety, environmental protection, opioid awareness, and during COVID-19 pandemic, behaviors like social distancing, masking, and vaccination. Democratic governments generally cannot force these behaviors on citizens; instead, they must persuade. Persuasive appeals mainly fall into three categories: endorsements (cues from leaders, experts, or celebrities), guidance and mandates (policies or practices issued by government), and information (the provision of facts and arguments about benefits). Using data from 10 experiments with 85,191 survey respondents conducted over a 2-year period during the COVID-19 pandemic, we assess the effectiveness of these three types of persuasive messages. We find that endorsements are variously polarizing depending on subjects’ partisan orientation toward the endorser, counterproductive in general, or wholly ineffective. We find that guidance and information treatments—when they are effective at all—move people “in parallel,” i.e. in the direction of information by similar amounts regardless of party affiliation.

Significance Statement Ten experiments conducted between 2020 and 2022 with 85,191 respondents on intentions to vaccinate and wear a mask show factual information and guidance can successfully encourage prosocial behavior among subjects from all partisan backgrounds but endorsements from political leaders and celebrities too frequently cause unintended decreases in prosocial behavior.

## Introduction

Governments routinely try to influence people to adopt prosocial behaviors. Often, these efforts focus on protecting the health, safety, and well-being of communities and citizens. Notable attempts from the 20th century at this kind of persuasion include the Partnership for a Drug Free America’s famous antidrug public service announcement, “this is your brain on drugs,” showcasing an egg being cracked open and fried in a pan; or Nancy Reagan telling America’s youth to “Just Say No” to drugs. In the 21st century, nonprofits have turned their attention toward mental health, gun safety, and awareness of the dangers of Fentanyl. As demonstrated by the Ad Council, a leading public service announcement creator, endorsements by celebrities (Megan Thee Stallion on mental health), guidance from policymakers (the CDC on preventing pre-Diabetes), and the provision of pure information (knowing the signs of Alzheimers) are trusted messaging strategies used by those who engage in promoting the public good. But studies of the effectiveness of these tactics are mixed. A segment on American Public Media’s radio program *Marketplace* reviewed the impact of antidrug messaging from the 1980’s and concluded there was “no good evidence” any of it changed behavior—and worse, that making drug use seem illicit may have led more teens to try it (1).

In 2020, during the SARS-CoV2 COVID-19 pandemic, governments around the world tried to induce their citizens to adopt behaviors that would slow the spread of the disease. These behaviors included postponing travel and visits with friends and family, social distancing, wearing a mask, and getting vaccines and boosters. Many people resisted the recommendations, but in the United States, most people complied. In March of 2020, just a week or so after the World Health Organization declared the disease a global pandemic, surveys showed that nearly everyone in the country was engaged in the COVID-19 mitigation strategies recommended by public health organizations. For example, 89% of Democrats and 84% of Republicans approved of canceling large gatherings and similarly high percentages approved of restricting travel (2). By the Fall of 2020, however, partisan divides had emerged, with roughly half of Republicans continuing to express support for travel restrictions while Democratic support remained higher. Doctors, public health experts, and governments across the country launched a wide variety of campaigns to convince people to protect themselves and those around them.

In order to assess the potential impact of these strategies in real time (and particularly the potential for differential effects by partisan affiliation), the UCLA COVID-19 Health and Politics Project began as a collaboration among social scientists at UCLA, medical doctors at UCLA and Harvard University, and public health officials from state and national government entities. The goal of the project was to provide real-time data on the comparative effectiveness of disease-mitigation messages that medical centers, the US Centers for Disease Control and Prevention, state governments, and other interested parties were using during a politically important time—the lead up to a presidential election. In some cases, the project tested messages that were already in use; in other cases, it tested ideas that might be put into practice. Of particular interest was finding messaging strategies that worked on people in both political parties as the campaign for president gained traction and the parties differentiated themselves on mitigation strategies generally.

In this article, we report findings from 10 messaging experiments (with 41 unique treatments) conducted among 85,191 survey respondents interviewed between 2020 and 2022. Though the project contained dozens more experimental tests, not all of them aimed to persuade people through the provision of information or endorsements (e.g. some tested whether incentives like cash payments or lotteries could change behavior and for whom). Here, we evaluate the potential impact of three strategies to persuade people using only information about mitigation strategies: endorsements of the vaccines by notable people, information on expert guidance and mandates related to the vaccine and wearing a mask, and factual information on vaccine effectiveness. We test the effects of these strategies on self-reported intentions to vaccinate and attitudes about wearing a mask to stop the spread of the virus.

Truth be told, we arrived at these strategies *adaptively*, as befits a research project focused on active policy experimentation. As we will show, our first experiments on endorsements by political figures and political figures underlined the dangers associated with that approach: endorsements by Trump polarized responses by party, endorsements by others either often had null or even negative effects on vaccine intentions. These initial findings prompted the team to investigate the effects of other approaches—the guidance and information experiments. While even these strategies are not always effective, they have similar effects among a wide swath of the public and so do not polarize the way the Trump endorsements did.

Our findings contribute to an emerging consensus in political science on the effects of political communication. Messages containing endorsements by political figures are a form of a “group cue” that have been extensively studied, especially in the form of party cues (e.g. (3–6)). Work in this area demonstrates that partisan cues to support a policy increase support among in-party respondents, but decrease support among out-party respondents. We find this precise pattern in response to Trump endorsements in October 2020. However, by April 2021, we find negative responses to endorsements by Presidents Obama and Biden among still-unvaccinated Democrats and Republicans alike, a finding that surprised us. Possible “explanations” for this pattern may include that still-unvaccinated Democrats as of April 2021 have very weak partisan attachments or they infer negative qualities of the vaccine on the basis of any endorsement.

Persuasive information (facts and information intended to change attitudes in a specific direction in the absence of a group partisan cue) has recently been shown by political scientists to move attitudes “in parallel” (7, 8). Our experiments also show this parallel pattern of effects: information about the effectiveness of vaccines and boosters increases the intention to get a vaccine or a booster by similar amounts for strong Republicans, strong Democrats, and everyone in between.^a^ To be clear, we while find that some information treatments have effects that are positive for all subgroups, we also find that other information treatments have effects that are close to zero for all subgroups. We consider both patterns to be instances of “parallel” updating, since both flat and positively sloped lines can be parallel.

In December of 2023, an interdisciplinary group of scholars summarized the insights from 747 articles written about behavioral science and COVID-19 policy-making (9). Our results are in line with their broad conclusions, and we provide detailed evidence regarding one of their 15 claims, namely that “Identifying trusted sources (e.g. local, religious, political, or community leaders) that are credible to different audiences to share public health messages can be effective in increasing intentions to engage in recommended health behaviors.” Specifically, we emphasize that people adopt behaviors recommended or mandated by *authorities* and that their intentions to vaccinate respond to straightforward information about contagiousness and effectiveness.

An important caveat emerges from our work. Authorities and trusted sources do better at moving people in the intended direction of their message when they are not expressly political figures. Politicians may want to do their part in (or claim credit for) promoting prosocial behaviors, whether those be disease mitigation strategies or any of the other important efforts mentioned above, but our findings suggest that when partisan actors engage in this type of work they may do more harm than good. Moreover, we find that there are simple ways to avoid backlash and nudge everyone in the same direction, at least by small amounts.

## Materials and methods

The UCLA COVID-19 Health and Politics Project is a nationwide, cross-sectional survey, representative of the US adult population. The project consists of eight survey waves spanning more than 2 years. The first four waves, consisting of 15,000 respondents each, were conducted between 2020 May 11 and 24; 2020 July 9 and 22; 2020 October 1 and 17; and 2020 December 4 and 16. Waves five through eight, which consist of 30,000 respondents each, were conducted 2021 March 25 to April 13, 2021 June 17 to July 6, 2021 September 3 to October 4, and 2022 October 24 to December 20. Nearly all waves of the survey contained randomized experiments, but the 10 experiments we report here were conducted in the third wave of the survey, as well as waves five through eight. Table 1 describes the timeline of these experiments.

**Table 1.** Directory of experiments

Experiment name	Date fielded	Sample definition	*N*	Treatment	Experimental groups	Outcome^a^
Endorsement						
E-1: Vaccine endorsement 1	10/2020 (wave 3)	All respondents	14,946	Vaccine endorser (e.g. Trump)	7 endorser treatments + 1 control	Likelihood of getting the vaccine
E-2: Vaccine endorsement 2	04/2021 (wave 5)	Unvaccinated^b^	7,249	Vaccine endorser (e.g. Trump)	8 endorser treatments + 1 control	Likelihood of getting the vaccine
Guidance and mandate						
G-1: CDC mask guidance 1	06/2021 (wave 6)	All respondents	30,857	CDC guidance	1 treatment + 1 control	Views on mask wearing
G-2: Vaccine mandate vignettes	06/2021 (wave 6)	Unvaccinated^b^	10,298	Vaccine requirement to do an activity	4 vignette arms: concert, restaurant, sports team, trip	Likelihood of getting the vaccine
G-3: CDC mask guidance 2	09/2021 (wave 7)	All respondents	33,088	CDC guidance	1 treatment + 1 control	Views on mask wearing
Information						
I-1: Contagiousness conversation	09/2021 (wave 7)	Unvaccinated^b^	8,710	Delta contagiousness information	3 conversation arms: friend, CDC, doctor	Likelihood of getting the vaccine
I-2: Delta variant conversation	09/2021 (wave 7)	Unvaccinated^b^	8,710	Delta contagiousness information	1 treatment + 1 control	Likelihood of getting the vaccine
I-3: Bivalent booster information	10/2022 (wave 8)	Unboosted^c^	10,700	Winter surge warning; booster, and vaccine information	1 treatment + 1 control	Likelihood of children getting the Bivalent booster
I-4: Bivalent booster information (children)	10/2022 (wave 8)	Respondent’s children unboosted^c^	1,628	Winter surge warning; booster, and vaccine information	1 treatment + 1 control	Likelihood of children getting the bivalent booster
I-5: Holiday surge information (children)	10/2022 (wave 8)	Respondent’s children unvaccinated^b^	1,715	Winter surge warning; booster, and vaccine information	1 treatment + 1 control	Likelihood of children getting the vaccine

^a^We convert 4- or 5-point Likert scales into binary measures, with the prosocial option coded as the higher value.

^b^Unvaccinated means the respondent answered that they have received no doses of any available COVID vaccine.

^c^Unboosted means the respondent indicated they have completed a full course of a primary vaccine but have not yet received a booster.

The respondents for the project were recruited by Lucid, a market research platform operating an online exchange. Our samples were constructed according to demographic quotas on age, gender, ethnicity, region, income, and education. Respondents were sent from Lucid directly to survey software operated by a UCLA team of researchers. All respondents completed the survey online and performed an attention check before starting. All surveys were conducted in English. These studies were reviewed and approved by the UCLA IRB (20-000786) and received informed consent from all participants.

The samples were weighted to be representative of the US adult population. We generated the weights using a relatively simple raking technique (which has been found to perform nearly as well as more complex alternatives (10)) as implemented in R package leafpeeper. The targets to which the surveys were weighted were derived from the US adult population of the 2017 American Community Survey of the US Census Bureau. The one exception is the 2016 vote, which was derived from the official election results released by the Federal Election Commission.

We weighted according to the following respondent information: gender, the four major census regions, race, Hispanic ethnicity, household income, education, age, language spoken at home, nativity (US- or foreign-born), 2016 presidential vote, and the urban–rural mix of the respondent’s ZIP code. We also weight on these interactions: Hispanic ethnicity by language spoken at home, education by gender, gender by race, race by Hispanic origin, race by education, and Hispanic origin by education. A detailed description of this survey methodology, including the representativeness of samples constructed using this approach, is available online (11). To get a sense of the representativeness of our data about COVID-19 mitigation, in Spring of 2021, the CDC estimated that 44.6% of the adult US population had received at least one dose of a COVID-19 vaccine. Our survey estimate for this same period is close at 48.8%.

We are aware that treatment effects estimates that are weighted are often less precise than unweighted estimates, with some suggesting that it might not be “worth weighting” (12) as a result. Since our estimands are defined at the population level, it is our view that we just have to accept the relatively lower precision. Nevertheless, in the Appendix, we present a comparison of weighted to unweighted estimates (Appendix Fig. S46). While most estimates match in terms of sign, significance, approximate magnitude, the weighted estimates are indeed less precise. Three of the partisan interaction terms that are not significant using weighted data become significant when unweighted data are used. All three are related to the effects of endorsements and in our view, further underline possibly polarizing effects of political endorsements.

Each wave of the project contained a set of common questions regarding respondents’ vaccination status, their concern about COVID-19, perceived risk of contracting COVID-19, and a series of batteries about general health and well-being. These were asked before any experimental treatments. Each wave also included a set of questions specific to the period of time in which the survey was fielded. For example, when schools were re-opening, we asked parents about whether they were comfortable sending their students back to school and when boosters became available, we asked respondents about their intentions to get booster shots.

Each survey ended with a final set of questions about political orientations (including party identification) and other demographic measures. This last set was always asked posttreatment, though a few demographics were passed to us from the sample provider to help fill the quotas detailed earlier.

Two key concepts for this project are party identification and vaccination status. We measure partisanship posttreatment (on posttreatment placement, see (13)) using the standard American National Election Study branching question that categorizes partisans on a scale from 1 (strong Democrat) to 4 (independent) to 7 (strong Republican). We measure a person’s vaccination status by asking respondents: “How many doses of a COVID-19 vaccine have you received to date, if any?” Respondents who indicated they had received at least one dose (regardless of manufacturer) were considered vaccinated. The full question wording is available in the Appendix.

Since intention to vaccinate is the main outcome in the endorsement and information experiments, we conducted those experiments only on unvaccinated respondents. (In the CDC guidance experiments, we intended to influence attitudes about wearing a mask, so we included the full sample regardless of vaccination status.) Restricting the sample to the unvaccinated affects both how we interpret and how we analyze the resulting data. In October 2020, vaccines were unavailable to everyone, so the first endorsement experiment is conducted among all subjects. Later endorsement and information experiments were conducted among the ever-shrinking fraction of the sample that remained unvaccinated. This design choice is purposeful—the goal of this study was to learn what messages lead the unvaccinated population to become vaccinated—but it does somewhat complicate cross-study comparisons of average causal effects. In the main text, we make no adjustment for the changing sample and present results according to the design. In the Appendix, we report estimates among the subgroup who, using a machine-learning model, we predict *will* be unvaccinated at the time of the final wave of the study. This approach facilitates comparisons across studies in the sense that the resulting sample is demographically similar across all waves. Results of this exercise show similar patterns of effect heterogeneity and homogeneity, though due to the relatively small sizes in the “predicted to be unvaccinated at the final wave” group, our uncertainty is correspondingly larger.

In each experiment, subjects were randomized into treatment conditions with equal probability (using Bernoulli “simple” random assignment). CONSORT diagrams detailing the precise survey flows for each experiment are presented in the Appendix. For each study, we estimate causal effects with a regression of the binary outcome variable on treatment assignment, with weights as described above. We estimate HC1 robust standard errors. We estimate the average treatment effect and also conditional average treatment effects by the seven levels of partisan identification. The analyses of these experiments were not preregistered owing to the fast pace of design required by our research partners’ dynamic policy environment.

For one experiment in each of the three messaging types, we present the difference-in-means estimate of the average treatment effect and of the conditional average treatment effects by each level of seven-point party identification in figure form.^b^ These figures give a visual sense of the patterns we uncover. Further, since we break our moderator (7-pt party identification) into seven discrete bins, we can reassure the reader that when we later linearize over these seven values, we have not masked important nonlinearites (14).

Indeed, the effect estimates in the tables use the Lin adjustment procedure (15), which amounts to an OLS regression of the outcome on treatment, interacted with mean-centered versions of the covariates. In addition to the precision gains offered by this procedure, it has some interpretative advantage. The intercept of this regression refers to the covariate-adjusted estimate of the average level of the outcome in the control group. The coefficient on the treatment variable represents the covariate-adjusted estimate of the average treatment effect. The coefficient on the interaction term has the usual interpretation, i.e. how much more effective the treatment is (on average) for people who score one scale point higher on the seven points of the party ID scale. In these regressions we adjust for the following demographic covariates: race/ethnicity, age, education, gender, and household income. Additionally, we adjust for several covariates specifically related to COVID-19: level of worry about COVID-19, perceived risk of getting COVID-19 (over 30 days, a lifetime, and relative to the average person of the same age), and whether the respondent received a flu shot in the study year. A comparison of the figures to the tables reveals that the two approaches yield substantively similar results.

In the Appendix, we present some alternative analyses. In addition to estimates without survey weights and estimates among the “predicted to be vaccinated” (Appendix Figs. S23–S45), we also present estimates of conditional average treatment effects (CATE) among partisans (including leaners) and the corresponding differences-in-CATEs for all experiments (Appendix Figs. S48–S61). These analyses confirm the models we present in the main text: the sign and significance of the interaction terms in the main text match the sign and significance of the difference-in-CATEs in all cases but one. Finally, since one of our claims is that the effects of the guidance and information treatments is similar across partisan groups, we also present equivalence tests to guard against “accepting the null” that there are no differences in effects when that difference may be estimated relatively imprecisely. We present tests with equivalence bands of 5 and 10 points. In the G-1 and G-3 mask guidance experiments, we can affirm equivalence at 5 points. G-2 is more nuanced; we can affirm equivalence at 10 points in two cases but cannot in two cases. Among the five information experiments, none of the differences-in-CATEs is significant, and we can affirm equivalence at 10 points in three cases but cannot in two.

## Results

### Endorsement experiments

In the October 2020 (E-1) experiment, we asked respondents: “If a safe and effective vaccine for COVID-19 were made easily available through a fast-track approval process at no cost to everyone in the next several weeks, how likely would you be to get it? Assume the vaccine has the following properties: It has only a few, mild side effects, like stiffness at the injection site. It would protect you from getting COVID-19 for at least a year [and would also help to protect others by not spreading the disease to people around you]. It was endorsed by [endorser].” In the 2021 experiment, the text was modified to indicate that the vaccine was already in use. We dichotomize the four response options: “very likely” and “somewhat likely” (1), and “somewhat unlikely” and “very unlikely” (0).

In E-1 set of endorsers included (i) their health insurance company, (ii) their pharmacy, (iii) their physician, (iv) religious/spiritual leaders, (v) President Donald Trump, (vi) Dr. Anthony Fauci, or (vii) both President Trump and Dr. Fauci; the control group saw no endorsement. In E-2, treatment group subjects could be assigned to any of eight endorsers: President Trump, Dr. Fauci, Trump and Fauci, NBA star LeBron James, Univision news anchor Jorge Ramos, President Barack Obama, President Joe Biden, and Biden and Fauci. In the October experiment (E-1), we split respondents into two arms: the first (Personal) told respondents the vaccine would offer a year of protection and the second (Social) added that the shot “would also help to protect others by not spreading the disease to people around you.” We conducted a joint significance test of the null hypothesis that the effects of the endorsements do not vary according to the Personal vs Social variation (p = 0.37); the 2021 (E-2) experiment does not feature this design wrinkle.

**Fig. 1.**
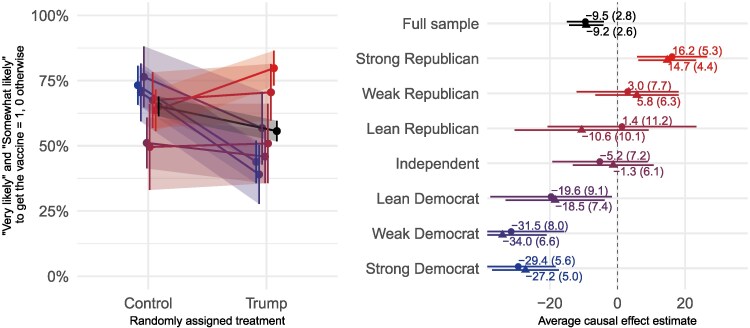
Effects of Trump endorsement on intentions to vaccinate (October 2020, experiment E-1) Plotting symbols on the right-side panel represent the conditional average treatment effects within each row. Circles show the effects without covariates. Triangles represent the estimates from a model with covariates as discussed earlier. Lines on the left-side panel represent the average effects overall (black topmost line) and for each level of partisanship (red for Republicans towards the top and blue for Democrats towards the bottom). Shades of the lines increase with increasing partisan intensity.

In Fig. 1, we present estimates of the effect of President Trump’s endorsement of the vaccine in October 2020. On average, Trump’s endorsement decreased people’s intentions to get vaccinated by more than 9 points (β=−0.092,SE=0.026). The conditional average treatment effects by levels of party identification suggest that Trump’s endorsement *polarized* intentions to vaccinate: the effect was negative for strong and weak Democrats as well as independents who lean towards the Democratic party; in contrast, it was positive among strong Republicans. This pattern can be seen in the visualization in the left panel of the figure, which shows blue lines sloping down (decreasing intentions) and red lines remaining flat or sloping up. The panel on the right side of Fig. 1 also shows the polarization pattern, as effect sizes move from the negative range (bottom-left) for Democrats through the middle of the figure for independents and up to the positive range for Republicans. The criss-crossing lines on the left panel and the diagonal pattern of plotting symbols on the right panel show how the effects of Trump’s endorsement were highly heterogeneous.

**Fig. 2.**
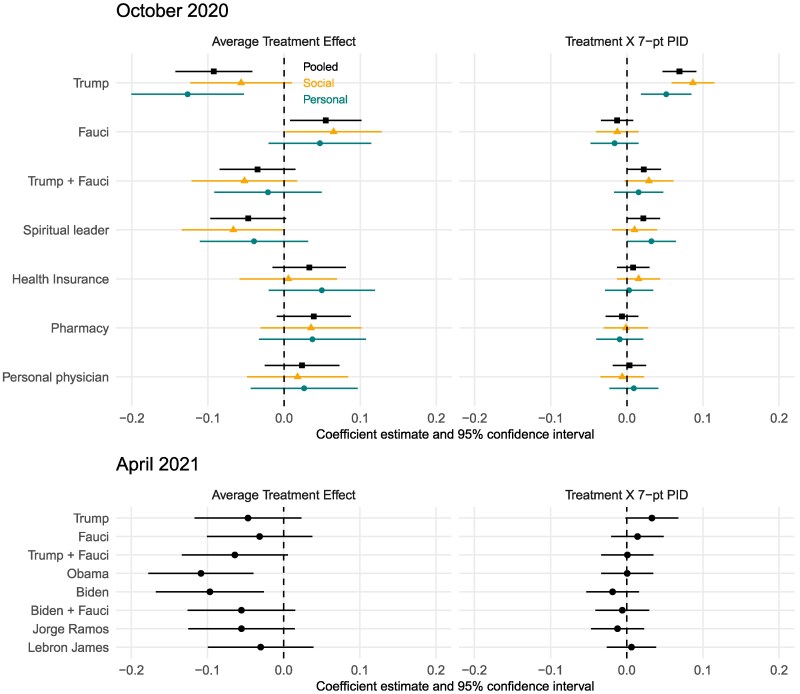
Average and heterogeneous effects of endorsers, October 2020 (E-1) and April 2021 (E-2) Estimates derived from the Lin regression model described in text, where the intercept corresponds to the control group mean, the coefficient on the treatment indicator corresponds to the average treatment effect, and the coefficient on the interaction term between treatment and party describes how much more effective the endorsement is for subjects who are more Republican on the 1–7 party identification scale.

The first row of Fig. 2 presents the coefficients from a regression model (represented by square plotting symbols) estimating the effect of Trump’s endorsement, as presented in Fig. 1. The model interacts treatment with party identification to simplify the presentation of the conditional average treatment effects in Fig. 1. The positive coefficient in column 2 of Fig. 2 indicates a heterogeneous treatment effect that is stronger for Republicans. Estimates from the personal arms are plotted with circles while estimates from the social arm that included additional information that the vaccine would protect other people are plotted with triangles. Rows 2 and 3 present two additional regression models for endorsements by Dr. Anthony Fauci, Director of the National Institute of Allergy and Infectious Diseases, and a third test of an endorsement by both Fauci and Trump. These regression models also control for covariates measuring several demographics and COVID-19 associated attitudes as mentioned above. The final four rows show the remaining endorsers tested in October 2020. Corresponding regression tables are presented in Appendix Tables S1–S3.

In the control group, roughly two-thirds of Americans expressed an intention to get the vaccine once it became available—even without an endorsement. As discussed above, Trump’s endorsement decreased intentions to vaccinate by more than 9 points (β=−0.092,SE=0.026) on average and polarized intentions by party identification. Fauci’s endorsement, in contrast, increased intentions to vaccinate by 5.5 points (β=0.055,SE=0.024) on average but, as a nonpolitical, medical expert, his endorsement did not have differential effects across partisan groups. This differential is captured by the interaction between the treatment and party identification. When Trump’s polarizing endorsement is countered with Fauci’s nonpolarizing endorsement, both the average negative effect of the Trump endorsement and its polarizing effect were neutralized. Altogether, Trump’s endorsement appears uniquely polarizing in October of 2020.^c^

The bottom panel of Fig. 2 presents results from the second set of endorsement experiments conducted in March and April 2021 (E-2). Very importantly, many Americans were vaccinated by this time, and our experiments were conducted among still unvaccinated respondents. Furthermore, by Spring of 2021, intentions to vaccinate were strongly associated with party identification, with Democrats being much more likely, on average, to report an intention to vaccinate in the future relative to Republicans. Among the 7,249 unvaccinated people we interviewed in Spring of 2021, 79% of the Democrats said they were likely to get the vaccine, whereas only 45% of Republicans said this. Independents were in the middle at 50%.

The findings from this wave of endorsements all suggest negative or null average effects and muted heterogeneity by partisanship (among still unvaccinated respondents). For example, newly elected President Biden’s endorsement decreased intentions to vaccinate among the remaining unvaccinated population on average by 9.7 points (β=−0.097,SE=0.036), with slightly more negative effects among Republicans and slightly less negative effects among Democrats, though these differences are not precisely estimated. The pattern of results for former President Obama are similar to Biden’s with large negative effects on intentions (β=−0.109,SE=0.035).

The politics of COVID shifted over this period, with President Trump moving to strongly criticize Dr. Fauci after failing to win re-election in November 2020. Our evidence partially reflects this story: in October 2020, we see positive effects of Fauci’s endorsement without much evidence of partisan heterogeneity, but by April 2021, the average effect of his endorsement has become negative and nonsignificant. Unfortunately, the comparison of these two tests does not pin down the changing environment because the two samples themselves are different. In October 2020, everyone was unvaccinated, but in April 2021, the unvaccinated is a different slice of society.^d^

### Guidance and mandates experiments

In June and September of 2021, the use of masks as a mitigation strategy was changing across time, space, and by vaccination status. In June, the CDC recommended that vaccinated people did not need to wear a mask indoors in public, but unvaccinated people did. By September, their recommendation changed following a surge in COVID-19 cases due to the Delta variant. The CDC returned to recommending that everyone should continue to wear a mask when indoors in public regardless of vaccination status. Our experiments on people’s compliance with wearing a mask to stop the spread of COVID-19 were fielded coinciding with this policy shift, one in June (G-1) and the other in September (G-3). We wanted to know whether there was any value in telling people a simple fact—that the CDC had made a recommendation about who should wear a mask and when. The change in their guidance on the matter provides an interesting wrinkle, allowing us to estimate the effects of expert guidance that is more restrictive and then less restrictive.

Our experiments prompted all respondents (vaccinated and unvaccinated) with this stem: “Thinking about wearing masks inside of public places, do you think…” The outcome categories provided respondents with three choices: “everyone should continue to do this for a little while longer regardless of vaccination status,” “everyone should stop doing this now regardless of vaccination status,” or “vaccinated people don’t need to do this but unvaccinated people do.” Respondents assigned to the control group saw the stem and outcome categories exactly as presented above. Respondents in the treatment group had the phrase “Following CDC recommendations,” added to the outcome category corresponding to the CDC’s position at the time (which was different in June and September). In June, this phrase came before “vaccinated people don’t need to do this, but unvaccinated people do,” and in September it came before the outcome “everyone should continue to do this for a little while longer regardless of vaccination status.”

**Fig. 3.**
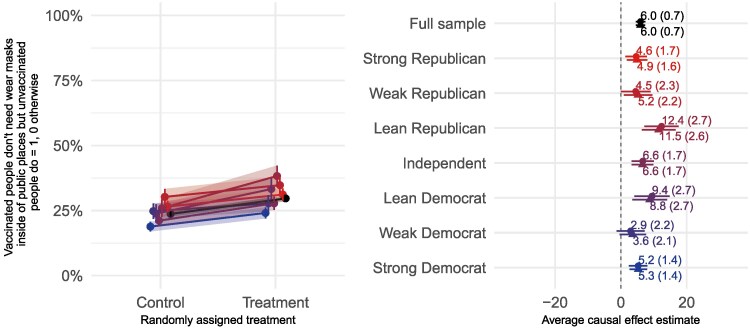
Effects of CDC guidance on attitudes about wearing a mask (June 2021, experiment G-1). Plotting symbols on the right side panel represent the conditional average treatment effects within each row. Circles show the effects without covariates. Triangles represent the estimates from a model with covariates as discussed earlier. Lines on the left-side panel represent the average effects overall (black topmost line) and for each level of partisanship (red for Republicans towards the top and blue for Democrats towards the bottom). Shades of the lines increase with increasing partisan intensity.

In Fig. 3, we present results from the June 2021 experiment that took place prior to the Delta variant surge (G-1). Adding a simple piece of information to the treatment indicating that the CDC was recommending one of the outcome options increased support for that policy by six points, on average—with no differential effects by partisanship. Everyone increased their support for mask-wearing tied to vaccination status when they learned the CDC was recommending it. We show these results in the form of the same two panels we presented for the endorsement experiments. The panels looks different, of course, as everyone moves in the same direction and by roughly the same amounts (though they start and end at different places). This pattern is “persuasion in parallel.” The treatment made everyone more likely to believe that vaccinated people did not need to wear masks indoors in public, but unvaccinated did, regardless of party affiliation.

In Table 2, we show the modeled results for the June and September guidance-experiments. In June, support for vaccine-based differences in mask-wearing policy was low—only 23.7% of respondents supported this mixed strategy that alerted people as to whether someone was vaccinated. But, as noted above, adding that this was the CDC’s recommendation increased support for the policy by 6 points with no heterogeneous effects by party. In September, baseline support for the more restrictive policy of making everyone wear a mask indoors in public was quite high, 68%. Still, adding the CDC treatment increased support by a point and a half on average. Again, persuasion occurred in parallel across partisan orientations.^e^

**Table 2.** Guidance experiments

	Less restrictive guidance (G-1)	More restrictive guidance (G-3)
(Intercept)	0.237^a^	0.680^a^
	(0.005)	(0.005)
Treatment	0.060^a^	0.015^a^
	(0.007)	(0.007)
Party ID (7-Point)	0.003	−0.053^a^
	(0.002)	(0.002)
Treatment × Party ID	−0.001	0.000
	(0.003)	(0.003)
Num.Obs.	30.751	32.933
$R^{2}$	0.073	0.153
Covariates	Yes	Yes
Sample	All respondents	All respondents

^a^ *P*<0.05.

By June 2021, some public officials had enacted vaccine requirements in order to participate in activities like travel or entertainment. To explore whether vaccine mandates increase intentions to get the vaccine, experiment G-2 randomized whether unvaccinated respondents were told that being vaccinated was required in order to do an activity. Subjects were randomized into one of four activities: going to a restaurant, a concert, a sports game, and taking a trip. We further varied whether the respondent was asked to consider the activity for themselves—or for a friend who would really enjoy the activity. The question in the “friend” arm was as follows: “Your [friend’s]  [favorite band is giving a concert near your town]. You know it would [be the perfect gift for your friend’s birthday and] it costs exactly what you had hoped to spend. [You want to surprise your friend with this gift.]  [‘Because’ or ‘Even though’] there will be lots of people together, proof of a COVID-19 vaccination [‘is’ or ‘is NOT’] required to enter the venue. Which of the following best describes what you would do in this situation?” We present results separately for each arm (friend and solo) and pooled. We dichotomize the five response options: “I would definitely get vaccinated and go” and “I would probably get vaccinated and go” (1), and “I would not get vaccinated and still try to go,” “I would probably not get vaccinated and stay home,” and “I would definitely not get vaccinated and stay home” (0).

**Fig. 4.**
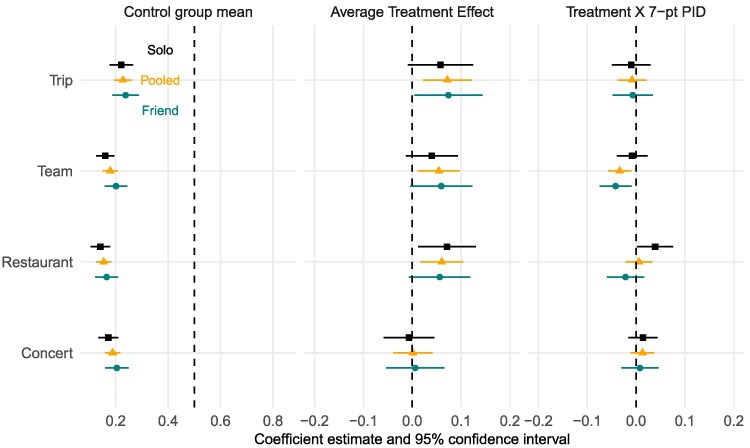
Effects of mandates on willingness to vaccinate (June–July 2021, experiment G-2) Estimates derived from the Lin regression model described in text, where the intercept corresponds to the control group mean, the coefficient on the treatment indicator corresponds to the average treatment effect, and the coefficient on the treatment × party interaction term describes how much more effective the endorsement is for subjects who are more Republican on the 1–7 party identification scale.

As these subjects are all unvaccinated, it is unsurprising that baseline levels of willingness to get vaccinated in order to participate in these activities was low, around 20%, as presented in column 1 of Fig. 4. The pooled average treatment effects for each vignette, displayed in column 2 by square plotting symbols, suggest that mandating vaccines to do things had small positive effects on respondents’ vaccine intentions in most cases (restaurants, sports, and trips) and null effects in others (concerts). Overall, the effects of the activity-specific mandate did not consistently vary depending on whether the activity was for the respondent or their friend).^f^ Mandates do not appear to have systematically polarized willingness to get the vaccine and altogether, these results suggest that vaccine requirements operate in a similar manner to guidance: on net, individuals move in parallel in response to mandates.

## Information experiments

By September of 2021, everyone in the country had access to a COVID-19 vaccine and most people had gotten at least one shot of a vaccine. Roughly 2 in 10 adults remained unvaccinated and finding ways to reach this resistant population became a priority of policymakers and medical professionals. Among public health experts, one thought was that mobile vaccination units might encourage people to get vaccinated if a doctor were able to talk to a patient and vaccinate them on site. Another idea that surfaced in 2022 was that reminding people about the holiday surge in 2021 and how many families had to cancel travel and vacation plans due to COVID-19 illness would motivate many unvaccinated people to protect themselves heading into the holidays in 2022.

To assess the effectiveness of these ideas, across 2021–2022 we fielded five experiments that provided respondents with accurate information about the virus, vaccines, and boosters (I-1 through I-5). In September 2021, we conducted two vignette experiments that placed people in an imaginary doctor’s office and gave respondents information about the contagiousness of the recent variant of the virus and the vaccine’s effectiveness in preventing serious illness (I-1 and I-2). In October of 2022 we abandoned vignettes and reminded people of the 2021 holiday surge, giving them information about the effectiveness of the Bivalent booster. We used this treatment to test three different outcomes (I-3 to I-5). Below we describe all five experiments before moving to a presentation of the effects.

Experiment I-1 conducted in September 2021 took the form of a vignette. In this experiment, unvaccinated adults were randomly assigned to three groups and asked to imagine that someone (either a friend, their doctor, or the CDC) was giving them information about the effects of COVID-19 and its contagiousness. Within each set, we randomly assigned people to treatment or control. Both were told that the virus had become more contagious, but the treatment group received specific information about how much more contagious the new variant was.

In the control group, we asked respondents: “Imagine [a friend, your doctor, the CDC] mentions that lots of unvaccinated people are being hospitalized for COVID-19 right now. Imagine [a friend, your doctor, the CDC] also says that it seems like the virus has become more contagious. Would this information make you more or less likely to get vaccinated?” In the treatment group, we offered respondents a more detailed description of the contagiousness, asking, “Imagine [a friend, your doctor, the CDC] mentions that over 90% of Americans in the hospital right now due to COVID-19 are unvaccinated. Imagine [a friend, your doctor, the CDC] also says that the Delta variant of the virus is more than twice as contagious as the original virus and that it is as contagious as Chicken Pox. Would this information make you more or less likely to get vaccinated?” Response options for both treatment and control were “more likely (1), less likely (0), or it wouldn’t affect my decision (0).”

Experiment I-2 also took the form of a vignette in which unvaccinated adults were asked to imagine that they were talking with their doctor. Respondents assigned to control were told that their doctor was urging them to get the vaccine. Treatment respondents were told to imagine they were having a conversation with their doctor in which the doctor was urging them to get the vaccine *and* that their doctor was emphasizing the increased contagiousness of the Delta variant of the virus. Respondents were asked what they would do in this scenario and given four outcome choices: let the doctor vaccinate them that day in the office (1), make an appointment to get vaccinated and keep it (1), make an appointment and cancel it (0), or decline to be vaccinated (0).

The Fall 2022 experiments leveraged information on the COVID-19 Bivalent booster shots. The (I-3) Bivalent booster experiment was fielded on people who had received a *full* vaccine course but had not yet received a booster shot. Control respondents were asked: “Knowing that another COVID-19 surge is likely between November and January, how likely are you to get the ‘Bivalent’ COVID-19 booster this year?” Treatment respondents were given more information about the expected surge in cases. They were asked: “Doctors and researchers are warning Americans that another COVID-19 surge will occur this Winter though they are not yet sure how it will compare to last year’s Omicron surge. The CDC reports that vaccines and boosters are the best way to protect yourself and your family against severe COVID-19 disease, potential long-term complications, and death. Knowing that another COVID-19 surge is likely between November and January, how likely are you to get the ‘Bivalent’ COVID-19 booster this year?” In both cases, respondents had five response options gauging their likelihood of getting the booster shot: “I will not get it (0), I am not very likely to get it (0), I am somewhat likely to get it (1), I am very likely to get it (1), or I will definitely get it (1).”

Experiments I-4 and I-5 build off the randomization in the previous experiment to conduct two additional tests on respondents who reported having children in their households. We asked respondents in the previous experiment (who had already been assigned to either treatment or control) to tell us their likelihood of getting a booster shot for their fully vaccinated but un-boosted children (I-4). If respondents in the previous experiment had children in their household who were not fully vaccinated, in I-5 we asked about their intentions to vaccinate their children (having already been exposed to either the treatment or control conditions above). Outcome categories were identical to those detailed above.

**Fig. 5.**
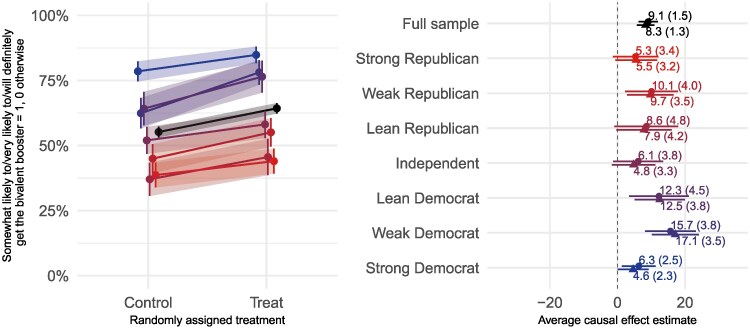
Effects of information about contagiousness on intentions to get booster shot (October 2022, experiment I-3) Plotting symbols on the right side panel represent the conditional average treatment effects within each row. Circles show the effects without covariates. Triangles represent the estimates from a model with covariates as discussed earlier. Lines on the left-side panel represent the average effects overall (black topmost line) and for each level of partisanship (red for Republicans towards the top and blue for Democrats towards the bottom). Shades of the lines increase with increasing partisan intensity.

Figure 5 presents the results of the 2022 Bivalent Booster experiment (I-3) among vaccinated adults who were not yet boosted. A familiar pattern emerges. On the left panel, we see that all of the effects move in the same direction, at roughly the same rate, regardless of party affiliation. As we saw with the guidance experiments, people start and end at different points, but the information about the contagiousness of the disease, the upcoming holiday surge, and the effectiveness of the booster increased vaccinated people’s intentions to get a booster shot by more than eight points, on average, and similarly across partisan groups. On the right panel, all of the estimates are positive and roughly in line.

In Table 3, we show the results for all five of the information experiments. Once again, partisanship has a direct effect on people’s intentions to vaccinate (themselves or their children) or to get the booster (for themselves or their children), but none of the treatment effects vary across partisan groups (see row four).

**Table 3.** Information experiments

	Contagiousness (I-1)	Delta (I-2)	Adult booster (I-3)	Child booster (I-4)	Child vaccine (I-5)
(Intercept)	0.197^a^	0.277^a^	0.556^a^	0.598^a^	0.391^a^
	(0.009)	(0.010)	(0.010)	(0.024)	(0.024)
Treatment	−0.010	0.016	0.083^a^	0.162^a^	0.057
	(0.013)	(0.014)	(0.013)	(0.032)	(0.034)
Party ID (7-Point)	−0.032^a^	−0.045^a^	−0.061^a^	−0.058^a^	−0.056^a^
	(0.006)	(0.006)	(0.005)	(0.013)	(0.011)
Treatment × Party ID	0.008	0.009	0.000	0.019	−0.001
	(0.008)	(0.009)	(0.006)	(0.017)	(0.017)
Num.Obs.	8.659	8.690	10.677	1.626	1.711
$R^{2}$	0.165	0.207	0.197	0.198	0.146
Covariates	Yes	Yes	Yes	Yes	Yes
Sample	Unvacc. adults	Unvacc. adults	Vacc. adults	Vacc. kids	Unvacc. kids
			No booster	In household	In household

^a^ *P*<0.05.

The vignette experiments, which encouraged unvaccinated respondents to get vaccinated based on requests from their doctor or other trusted sources had relatively large effects even when detailed information was absent (in the control groups). For example, in the “Delta” experiment (I-2), 28% of unvaccinated people reported that if they were in their doctor’s office and the doctor was urging them to get vaccinated, they would either get the vaccine that day or make an appointment to get it in the future and keep the appointment. In experiment I-1, 20% said they would get vaccinated at the request of a doctor, friend, or public health agency. Importantly, most of these people told us earlier in the survey that they would probably get vaccinated at some point in time, even though they had not yet done so. This within-subject consistency of intentions lends credence to our measurement, method, and design. Adding detailed information about the new variant being twice as contagious as the original and as contagious as the Chicken Pox; or information about the rate of hospitalizations for those without vaccines (in the treatment groups) did not significantly increase intentions to vaccinate.^g^ By Fall of 2021, the remaining unvaccinated people were very hard to move.

Between the 2021 information experiments and the October 2022 information experiments, the Omicron variant swept the nation and shut most things down again over the holidays. Many people were caught unprepared for the virus’s swift spread and had to cancel family visits and vacations. When January came, many students found themselves back to remote learning as some states and institutions returned to mitigation in the form of isolation.

Columns 3–5 of Table 3 show the results for the October 2022 experiments reminding vaccinated respondents about the possibility of another holiday surge that might resemble 2021’s Omicron surge (I-3 through I-5). These experiments also delivered information about the effectiveness of the booster shot. Among adults, as discussed earlier in the detailed figure, the increased information increased intentions to get the booster by 8.3 points (β=0.083,SE=0.013) net of other factors, with no evidence of heterogeneity by party.

We also asked people with vaccinated children in their households whether they would get their children a booster shot (for those who were eligible by age). Here, too, we see large effects of the information on intentions to get children a booster. Though nearly 60% of vaccinated parents intended to get their children a booster even without the treatment, providing the additional information about the expected upcoming Winter surge increased parents’ intentions to boost their vaccinated children by 16.2 points (β=0.162,SE=0.032). This effect estimate is the largest of any message we tested and the results do not vary significantly by the party affiliation of the parent being interviewed. All vaccinated parents were more likely to get their vaccinated children a booster shot after reading detailed information about it, regardless of their partisanship.

We also asked vaccinated parents of unvaccinated children if they were likely to get these children a vaccine dose after reading about the upcoming holiday surge and effectiveness of vaccines and the booster. The treatment was not effective and showed no signs of differential effects across party.

## Discussion

In the midst of a politicized global pandemic, we partnered with health authorities to study the effectiveness of various messaging strategies to affect people’s willingness to vaccinate and to engage in other prosocial behaviors to mitigate the spread of COVID-19. The evolution of our experimental designs was driven by medical professionals, clinicians, policy-makers like the US Centers for Disease Control and Prevention, and other state-government actors, many of whom we met with regularly. The progression of our experimental program reflects the learning we (and they) did about what was working in practice and what could be tried going forward.

Partisan endorsers often caused decreases in vaccination intentions overall and, in the instance of President Trump, polarized responses. Providing expert guidance and delivering nonpartisan information often had positive average effects and rarely had polarizing effects. Despite the politicized nature of virus-mitigation and a hard-fought presidential election in 2020, our study identifies multiple classes of messages that were effective at increasing intentions to vaccinate, wear masks, and get booster shots for people across the political spectrum. The final figure in the Appendix (Fig. S62) collects together the average treatment effects and interaction term estimates from all studies presented here.

Our findings speak to ongoing debates in political science about information processing and attitude change (7, 16, 17), and the role of partisan antipathy in shaping attitudes (18, 19). We extend tests of the notion that information can move people “in parallel” to a crisis domain, and demonstrate that despite partisans holding vastly different attitudes about mitigating COVID-19, information, and guidance delivered by nonpartisan sources can persuade everyone.

Our results also confirm the importance of party cues in at least one instance, showing that when partisan actors endorsed vaccines, the effects polarized by party. All our other experiments, however, show that when confronted with information and guidance from government on a politicized health crisis, a respondent’s party does not interact with that information to result in opposite effects by party. Generalizing from this set of cue effects (polarizing for Trump cues, homogeneously negative or null for the others we consider) is challenging. Both of the leading models of the mechanics of party cue effects (the “identity” model that holds people are motivated to bring their views in line with their identity and the “inference” model that holds people draw inferences about the policy based on heuristics about the cue giver) can accommodate this pattern, so we might venture that cues will be more polarizing the stronger are identities or heuristics. Confirmation of this hunch must await a systematic experimental program that measures identities, heuristics, and cue effects in a wide variety of settings.

Even so, we find just one instance cues working as intended across all audiences (the E-1 Fauci endorsement). By contrast, the guidance and information treatments change the attitudes of Democrats and Republicans (and everyone in between) in a similar fashion in about half the opportunities, with homogeneous nulls in the other half. This possibility of parallel attitude change across party groups on prosocial behaviors is important as the nation attempts to mitigate other social challenges like gun violence, homelessness, mental health crises, opioid addiction, the prevalence of Fentanyl, and climate change. COVID-19 affected many lives and families in tragic ways, but one takeaway from the pandemic is that nonpartisan, prosocial informational, and guidance-based messaging can be effective in improving outcomes.

## Supplementary Material

pgaf185_Supplementary_Data

## Data Availability

The full anonymized datasets and code necessary to reproduce all analysis and figures contained in the main text and the Appendix are available on OSF https://osf.io/bwv3c/.

## Notes

a When we use the term “information” in this article, we always mean “persuasive information” as defined above. Endorsements are, of course, a kind of information too—they are information about who supports what—but we maintain a sharp distinction between endorsements and persuasive information.

b The equivalent figures for the other experiments are presented in the Appendix Figs. S1–S22.

c A formal hypothesis test shows that the difference-in-interactions for the Trump and physician treatments (0.068  (0.012)) is statistically significant at P<0.001.

d When we try to compare apples to apples in terms of sample composition (i.e. those predicted to be unvaccinated at the end of the study period in both waves), we see the same pattern insofar as the effect is positive in October 2020 but null in April 2021 (see Appendix).

e Both of these guidance experiments (G-1 and G-3) collapse a three point outcome scale to a binary scale based on the CDC information category. Full distributional comparisons with associated $\chi^{2}$ tests show differences between treatment and control groups as well (see Appendix Fig. S47).

f We conducted a joint significance test against the null hypothesis that the average and interactive effects of the solo and friend versions are the same; we fail to reject the null (P=0.10)

g In the Appendix, we also show that the source of the information (CDC, doctor, or friend) did not independently affect vaccination intentions. We conduct a joint significance test against the null hypothesis that the average and interaction effects are the same across sources; we fail to reject this null (P=0.91).
