# Supplementary material for: Endorsements vs. information: Experimental evidence of backlash and parallel persuasion during the COVID-19 public health crisis
Source: PNAS Nexus. 2025 Jun 5;4(6):pgaf185. doi: 10.1093/pnasnexus/pgaf185 (PMC12205431; doi:10.1093/pnasnexus/pgaf185)

Online Appendix for Endorsements vs. Information: Experimental Evidence of Backlash and Parallel Persuasion during the COVID-19 Public Health Crisis

Ryan Baxter-King, Alexander Coppock, Graham Straus, and Lynn Vavreck

May 30, 2025

Contents

A Regression tables **3**

B Consort diagrams **7**

C Figures for each experimental contrast **18**

D Estimates among the "predicted to be unvaccinated" **30**

E Additional figures and analysis **42**
E.1 General Analyses . . . 42
E.2 Endorsement Experiments . . . 44
E.3 Guidance Experiments . . . 46
E.4 Information Experiments . . . 60

F Survey instruments **63**
F.1 Activity-Specific Mandates Experiment: June – July 2021 . . . 63
F.2 CDC Mask Guidance Experiments . . . 64
F.3 I-1: Contagiousness Conversation . . . 65
F.4 I-2: Delta Variant Conversation . . . 67
F.5 Bivalent Booster Experiments . . . 67
F.5.1 Step 1: Measure Bivalent Booster Uptake . . . 67
F.5.2 Step 2: Deliver Information About Potential Surge . . . 68
F.5.3 Step 3: Measure Intentions Towards Childrens' Vaccinations . . . 69
F.6 Measuring Party Identification . . . 69
F.7 Measuring Vaccination Status . . . 70
F.7.1 Vaccine Intent: October 2020 and December 2020 . . . 70
F.7.2 Vaccine Uptake: March - April 2021, June - July 2021, and September – October 2021 . . . 70
F.7.3 Vaccine Uptake: September 2022 . . . 71

G Estimates from all studies **72**

A Regression tables

Table S1: Endorsement experiment 1 (E-1)

	Trump	Fauci	Trump and Fauci	Spiritual leader	Health Insurance	Pharmacy	Personal physician
(Intercept)	0.653*	0.650*	0.652*	0.652*	0.650*	0.645*	0.649*
	(0.018)	(0.018)	(0.018)	(0.018)	(0.018)	(0.018)	(0.018)
Treatment	−0.092*	0.055*	−0.035	−0.047	0.033	0.039	0.024
	(0.026)	(0.024)	(0.025)	(0.025)	(0.025)	(0.025)	(0.025)
Party ID (7-Point)	−0.008	−0.008	−0.008	−0.008	−0.008	−0.008	−0.008
	(0.008)	(0.008)	(0.008)	(0.008)	(0.008)	(0.008)	(0.008)
Treatment x Party ID	0.069*	−0.013	0.022	0.022	0.008	−0.006	0.003
	(0.011)	(0.011)	(0.012)	(0.011)	(0.011)	(0.011)	(0.011)
Num.Obs.	3680	3665	3725	3678	3733	3735	3766
R2	0.179	0.202	0.176	0.166	0.170	0.170	0.163
covariates	yes	yes	yes	yes	yes	yes	yes

* $p < 0.05$

Table S2: Endorsement experiment 2 (E-2)

	Trump	Fauci	Trump and Fauci	Obama	Biden	Biden and Fauci	Jorge Ramos	Lebron James
(Intercept)	0.636*	0.629*	0.640*	0.627*	0.630*	0.624*	0.633*	0.623*
	(0.025)	(0.026)	(0.025)	(0.026)	(0.026)	(0.026)	(0.025)	(0.026)
Treatment	−0.047	−0.032	−0.064	−0.109*	−0.097*	−0.056	−0.055	−0.030
	(0.036)	(0.035)	(0.036)	(0.035)	(0.036)	(0.036)	(0.036)	(0.035)
Party ID (7-Point)	−0.061*	−0.061*	−0.061*	−0.061*	−0.061*	−0.061*	−0.061*	−0.061*
	(0.012)	(0.012)	(0.012)	(0.012)	(0.012)	(0.012)	(0.012)	(0.012)
Treatment x Party ID	0.033	0.014	0.001	0.001	−0.019	−0.006	−0.012	0.006
	(0.018)	(0.018)	(0.017)	(0.017)	(0.018)	(0.018)	(0.018)	(0.017)
Num.Obs.	1604	1596	1543	1613	1563	1567	1586	1634
R2	0.165	0.230	0.228	0.244	0.233	0.229	0.263	0.243
covariates	yes	yes	yes	yes	yes	yes	yes	yes

$*p < 0.05$

Table S3: Mandate Experiment (G-2)

	Concert (F)	Restaurant (F)	Team (F)	Trip (F)	Concert (S)	Restaurant (S)	Team (S)	Trip (S)
(Intercept)	0.204*	0.165*	0.201*	0.238*	0.172*	0.142*	0.160*	0.221*
	(0.023)	(0.022)	(0.022)	(0.026)	(0.020)	(0.019)	(0.018)	(0.023)
Treatment	0.006	0.056	0.059	0.074*	−0.007	0.071*	0.040	0.058
	(0.030)	(0.032)	(0.032)	(0.036)	(0.027)	(0.030)	(0.027)	(0.034)
Party ID (7-Point)	−0.052*	−0.020	−0.036*	−0.018	−0.052*	−0.046*	−0.038*	−0.035*
	(0.015)	(0.013)	(0.011)	(0.015)	(0.012)	(0.013)	(0.010)	(0.013)
Treatment x Party ID	0.008	−0.022	−0.042*	−0.007	0.014	0.039*	−0.008	−0.010
	(0.019)	(0.019)	(0.017)	(0.021)	(0.015)	(0.019)	(0.016)	(0.020)
Num.Obs.	1270	1322	1234	1316	1283	1362	1224	1264
R2	0.244	0.173	0.199	0.172	0.229	0.194	0.213	0.151
covariates	yes	yes	yes	yes	yes	yes	yes	yes

* $p < 0.05$
(F) and (S) indicate the ”friend” and ”solo” versions of the vignettes.

B Consort diagrams

E-1: Vaccine Endorsement Experiment

Dates Fielded: 10/01/2020 - 10/17/2020
N: 14,946

Arm A: Personal

N: 7,456

If a safe and effective vaccine for COVID-19 were made easily available through a fast-track approval process at no cost to everyone in the next several weeks, how likely would you be to get it? Assume the vaccine has the following properties:

- It has only a few, mild side effects, like stiffness at the injection site
- It would protect you from getting COVID-19 for at least a year
- It was endorsed by [Field-TREATMENT]

- Control; N: 922/924
- Your local pharmacy or the one that fills your prescriptions; N: 943/949
- A spiritual or religious leader; N: 950/950
- Health insurance company or insurer such as Medicare or Medicaid; N: 942/943
- Your personal physician; N: 969/970
- Donald Trump and Dr. Anthony Fauci, and other scientific sources; N: 896/898
- Donald Trump; N: 937/940
- Dr. Anthony Fauci (Director of the U.S. National Institute of Allergy and Infectious Disease), and other scientific sources; N: 879/882

Arm B: Social

N: 7,490

If a safe and effective vaccine for COVID-19 were made easily available through a fast-track approval process at no cost to everyone in the next several weeks, how likely would you be to get it? Assume the vaccine has the following properties:

- It has only a few, mild side effects, like stiffness at the injection site
- It would protect you from getting COVID-19 for at least a year and would also help to protect others by not spreading the disease to people around you
- It was endorsed by [Field-TREATMENT]

- Control; N: 924/927
- Your local pharmacy or the one that fills your prescriptions; N: 947/947
- A spiritual or religious leader; N: 884/886
- Health insurance company or insurer such as Medicare or Medicaid; N: 945/949
- Your personal physician; N: 952/953
- Donald Trump and Dr. Anthony Fauci, and other scientific sources; N: 983/987
- Donald Trump; N: 897/899
- Dr. Anthony Fauci (Director of the U.S. National Institute of Allergy and Infectious Disease), and other scientific sources; N: 941/942

Response Options:

•Very likely (1) •Somewhat likely (1) •Somewhat unlikely (0) •Very unlikely (0)

E-2: Vaccine Endorsement Experiment

Dates Fielded: 03/24/2021 - 04/14/2021
N: 14,557

Unvaccinated,
Include
N: 7,249

Now that a safe and effective vaccine for COVID-19 will be easily available to everyone at no cost in the next several weeks, how likely will you be to get it? Assume the vaccine has the following properties:

- It has only a few, mild side effects, like stiffness at the injection site
- It will protect you from getting COVID-19 for at least a year
- It is endorsed by [Field-TREATMENT]

Vaccinated (at least one dose),
Exclude
N: 7,308

- Control; N: 788/791
- Barack Obama; N: 828/833
- Donald Trump; N: 817/822
- Joe Biden; N: 788/791
- Jorge Ramos; N: 800/806
- Lebron James; N: 848/850
- Dr. Anthony Fauci (Dirctor of the U.S. National Isnstitute of Allergy and Infectious Disease), and other scientific sources; N: 809/811
- Donald Trump and Dr. Anthony Fauci (Dirctor of the U.S. National Isnstitute of Allergy and Infectious Disease), and other scientific sources; N: 758/764
- Joe Biden and Dr. Anthony Fauci (Dirctor of the U.S. National Isnstitute of Allergy and Infectious Disease), and other scientific sources; N: 783/787

Response Options:

•Very likely (1) •Somewhat likely (1) •Somewhat unlikely (0) •Very unlikely (0)

G-1: CDC Mask Advice Experiment

Dates Fielded: 06/17/2021 - 07/06/2021
N: 30,857

Control;
N: 15,449/15,493

Thinking about wearing masks inside of public places, do you think...

Treat; CDC Mask Option,
N: 15,311/15,364

Thinking about wearing masks inside of public places, do you think...

Control Response Options:
- Everyone should continue to do this for a little while longer regardless of vaccination status (0)
- Everyone should stop doing this now regardless of vaccination status (0)
- Vaccinated people don't need to do this but unvaccinated people do (1)

Treat Response Options:
- Everyone should continue to do this for a little while longer regardless of vaccination status (0)
- Everyone should stop doing this now regardless of vaccination status (0)
- Following CDC recommendations, vaccinated people don't need to do this but unvaccinated people do (1)

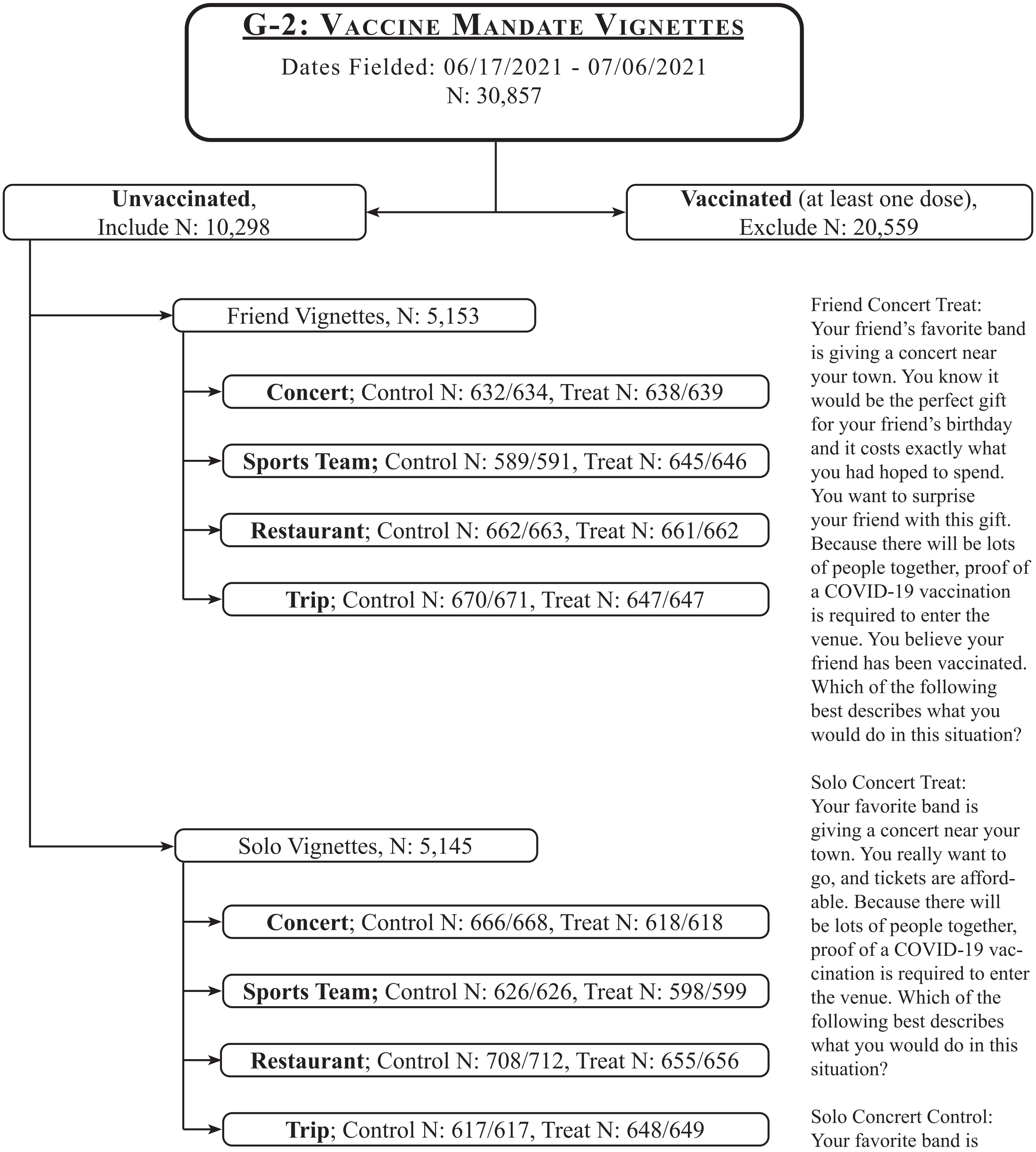

Friend Concert Treat:
Your friend's favorite band is giving a concert near your town. You know it would be the perfect gift for your friend's birthday and it costs exactly what you had hoped to spend. You want to surprise your friend with this gift. Because there will be lots of people together, proof of a COVID-19 vaccination is required to enter the venue. You believe your friend has been vaccinated. Which of the following best describes what you would do in this situation?

Solo Concert Treat:
Your favorite band is giving a concert near your town. You really want to go, and tickets are affordable. Because there will be lots of people together, proof of a COVID-19 vaccination is required to enter the venue. Which of the following best describes what you would do in this situation?

Solo Concrert Control:
Your favorite band is giving a concert near your town. You really want to go, and tickets are affordable. Even though there will be lots of people together, proof of a COVID-19 vaccination is NOT required to enter the venue. Which of the following best describes what you would do in this situation?

Response Options:
- I would definitely get vaccinated and go (1)
- I would probably get vaccinated and go (1)
- I would not get vaccinated and still try to go (0)
- I would probably not get vaccinated and stay home (0)
- I would definitely not get vaccinated and stay home (0)

G-3: CDC Mask Advice Experiment

Dates Fielded: 09/03/2021 - 10/05/2021
N: 33,088

Control;
N: 16,501/16,567

Thinking about wearing masks inside of public places, do you think...

Treat; CDC Mask Option,
N: 16,449/16,521

Thinking about wearing masks inside of public places, do you think...

Control Response Options:
- Everyone should continue to do this for a little while longer regardless of vaccination status (1)
- Everyone should stop doing this now regardless of vaccination status (0)
- Vaccinated people don't need to do this but unvaccinated people do (0)

Treat Response Options:
- Following CDC recommendations, everyone should continue to do this for a little while longer regardless of vaccination status (1)
- Everyone should stop doing this now regardless of vaccination status (0)
- Vaccinated people don't need to do this but unvaccinated people do (0)

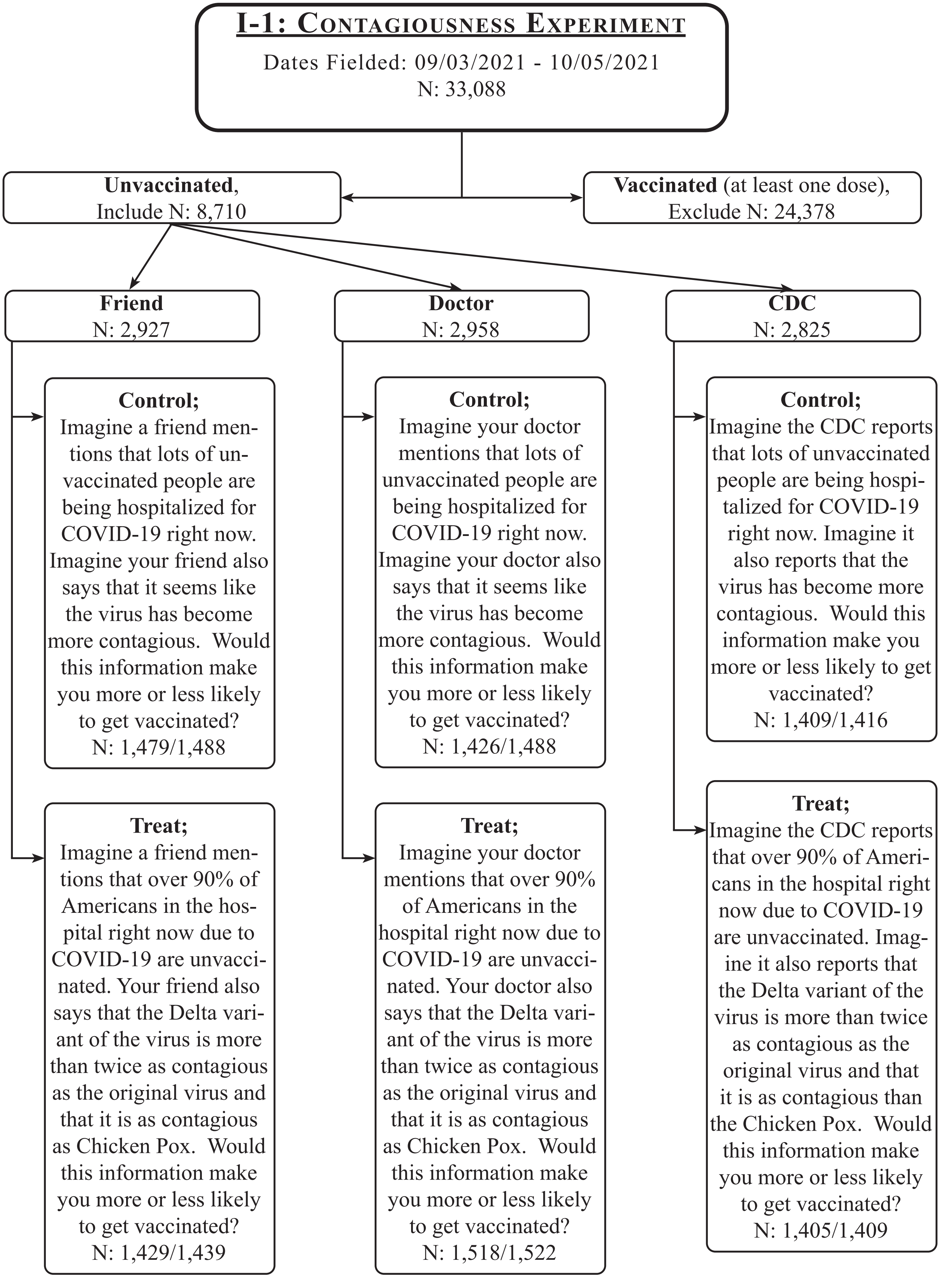

Response Options:

• More likely (1) • Less likely (0) • It wouldn't affect my decision (0)

I-2: Delta Conversation Experiment

Dates Fielded: 09/03/2021 - 10/05/2021
N: 33,088

Unvaccinated,
Include N: 8,710

Vaccinated (at least one dose),
Exclude N: 24,378

Control;
Imagine you’re having a conversation with your doctor about the way businesses in your neighborhood are reacting to the state of the virus. Your doctor listens to your concerns about the vaccine and understands your worry but emphasizes the risks are minimal. Your doctor urges you to get a vaccine shot. Would you…
N: 4,357/4,364

Treat; Delta
Imagine you’re having a conversation with your doctor about the way businesses in your neighborhood are reacting to the spread of the highly contagious Delta variant of the virus. Your doctor listens to your concerns about the vaccine and understands your worry but emphasizes the increased contagiousness of Delta, saying the risks are minimal. Your doctor urges you to get a vaccine shot. Would you…
N: 4,340/4,346

Response Options:
- Let the doctor vaccinate you in the office that same day (1)
- Make an appointment to get vaccinated later and keep it (1)
- Make an appointment to get vaccinated later and cancel it (0)
- Decline to be vaccinated (0)

I-3: Bivalent Booster Experiment

Dates Fielded: 10/24/2022 - 12/20/2022
N: 29,448

Not fully vaccinated;
Exclude N: 12,951

Have you received a dose of a "bivalent" COVID-19 booster since August 31st, 2022?

No;
Include N: 10,700

Yes;
Exclude N: 4,380

Not sure (I got a booster shot since August 31, but don't know if it was the new one or the original);
Exclude N: 4,380

Control;
How likely are you to get the "bivalent" COVID-19 booster this year?
N: 5,372/5,389

Treat;
Doctors and researchers are warning Americans that another COVID-19 surge will occur this Winter though they are not yet sure how it will compare to last year's Omicron surge. The CDC reports that vaccines and boosters are the best way to protect yourself and your family against severe COVID-19 disease, potential long-term complications, and death.
Knowing that another COVID-19 surge is likely between November and January, how likely are you to get the "bivalent" COVID-19 booster this year?
N: 5,308/5,311

Response Options:

- I will definitely get it (1)
- I am very likely to get it (1)
- I am somewhat likely to get it (1)
- I am not very likely to get it (0)
- I will not get it (0)

I-4: Child Bivalent Booster Experiment

Dates Fielded: 10/24/2022 - 12/20/2022
N: 29,448

No children/Children not fully vaccinated;
Exclude N: 23,464

Have any of the eligible children in your household received a dose of the "bivalent" COVID-19 booster since October 12, 2022?

No;
Include N: 2,755

Yes;
Exclude N: 2,577

Not sure (They got a booster shot since August 31, but don't know if it was the new one or the original);
Exclude N: 642

Not in bivalent experiment;
Exclude N: 1,127

Control;
How likely are you to get the children living in your household the "bivalent" COVID-19 booster this year?
N: 805/806

Treat;
Doctors and researchers are warning Americans that another COVID-19 surge will occur this Winter though they are not yet sure how it will compare to last year's Omicron surge. The CDC reports that vaccines and boosters are the best way to protect yourself and your family against severe COVID-19 disease, potential long-term complications, and death.
How likely are you to get the children living in your household the "bivalent" COVID-19 booster this year?
N: 822/822

Response Options:
- I will definitely do it (1)
- I am very likely to do it (1)
- I am somewhat likely to do it (1)
- I am not very likely to do it (0)
- I will not do it (0)

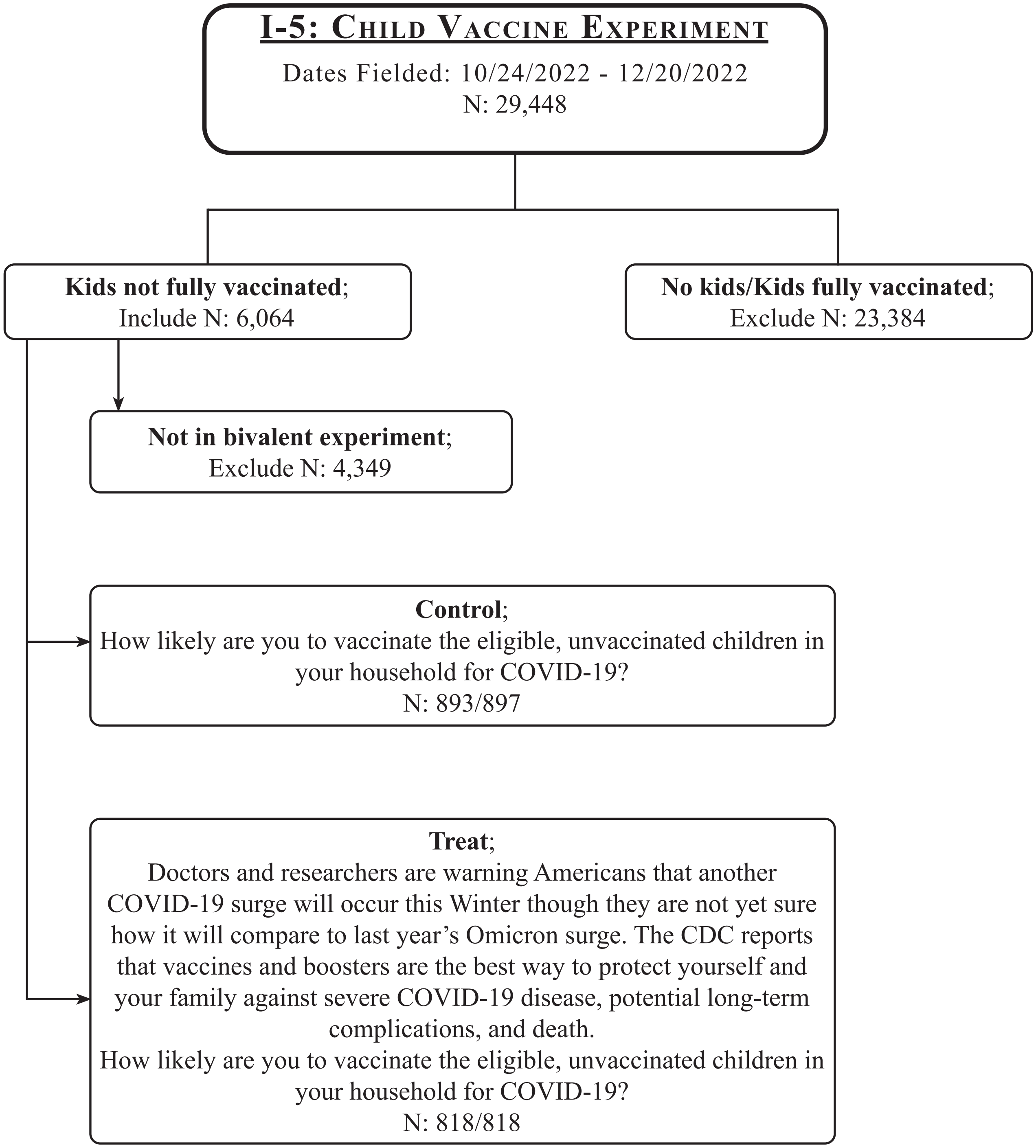

Response Options:

- Extremely likely (1)
- Very likely (1)
- Somewhat likely (1)
- Not at all likely (0)
- Not sure (0)

C Figures for each experimental contrast

Figure S1: Endorsement experiment E-1: Trump

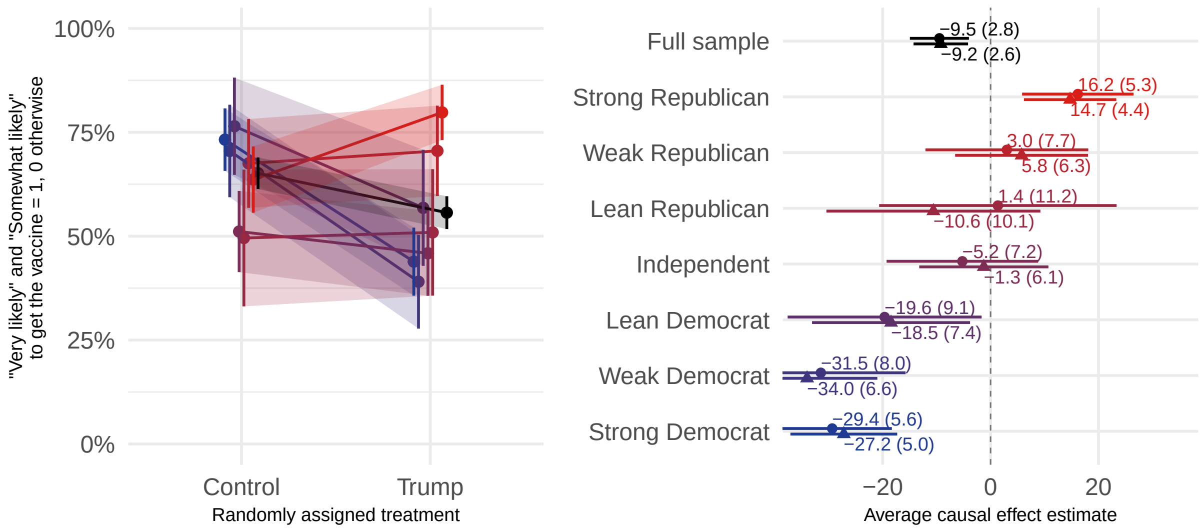

Figure S2: Endorsement experiment E-1: Fauci

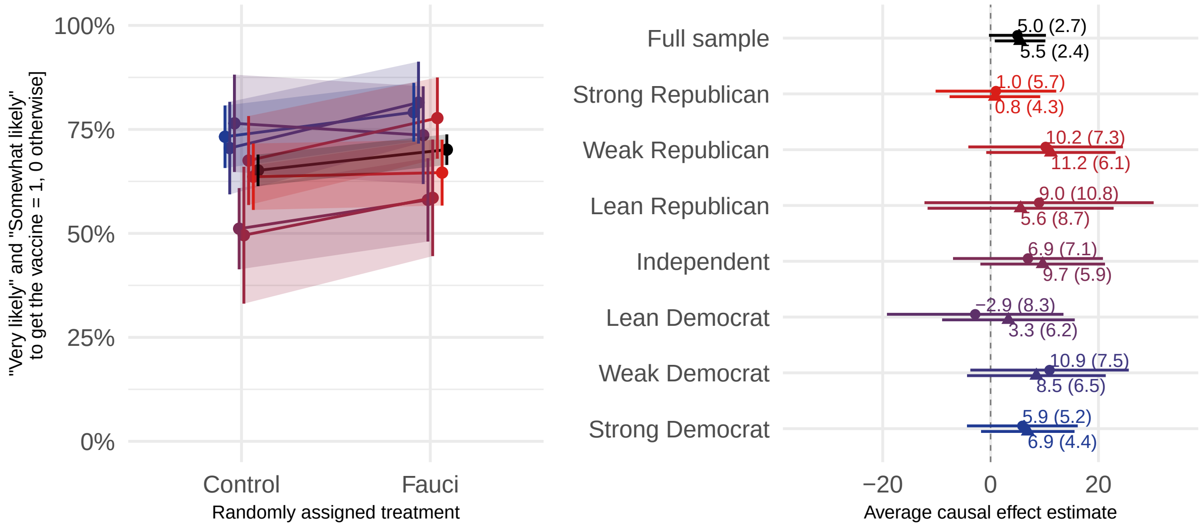

Figure S3: Endorsement experiment E-1: Trump and Fauci

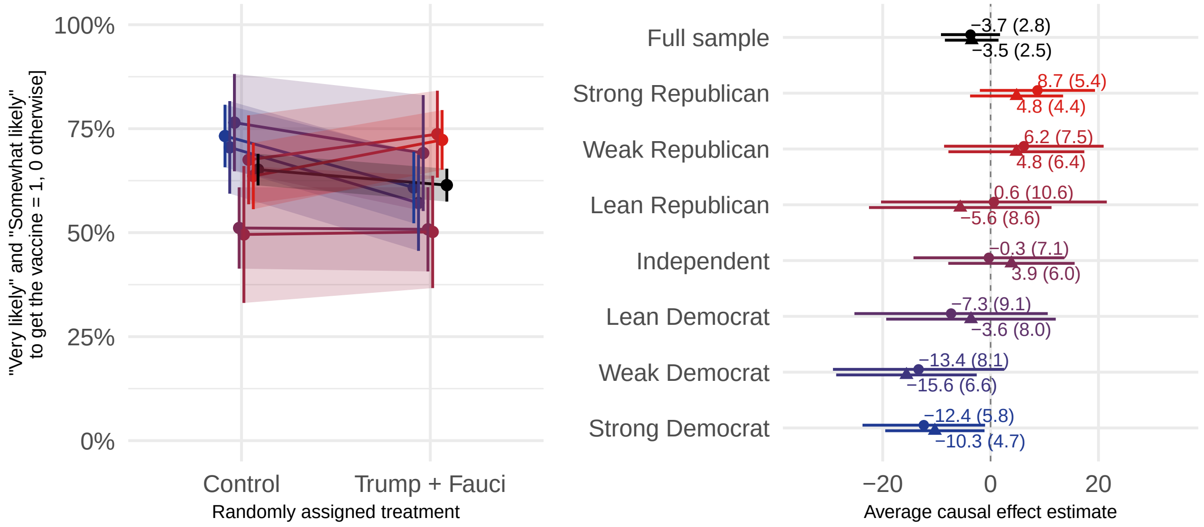

Figure S4: Endorsement experiment E-1: Physician

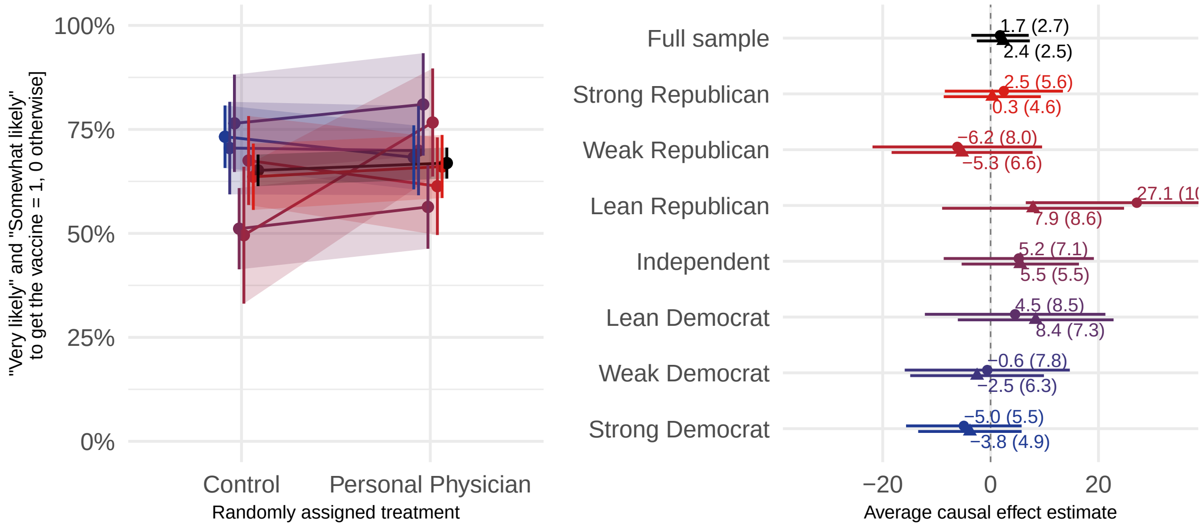

Figure S5: Endorsement experiment E-1: Pharmacy

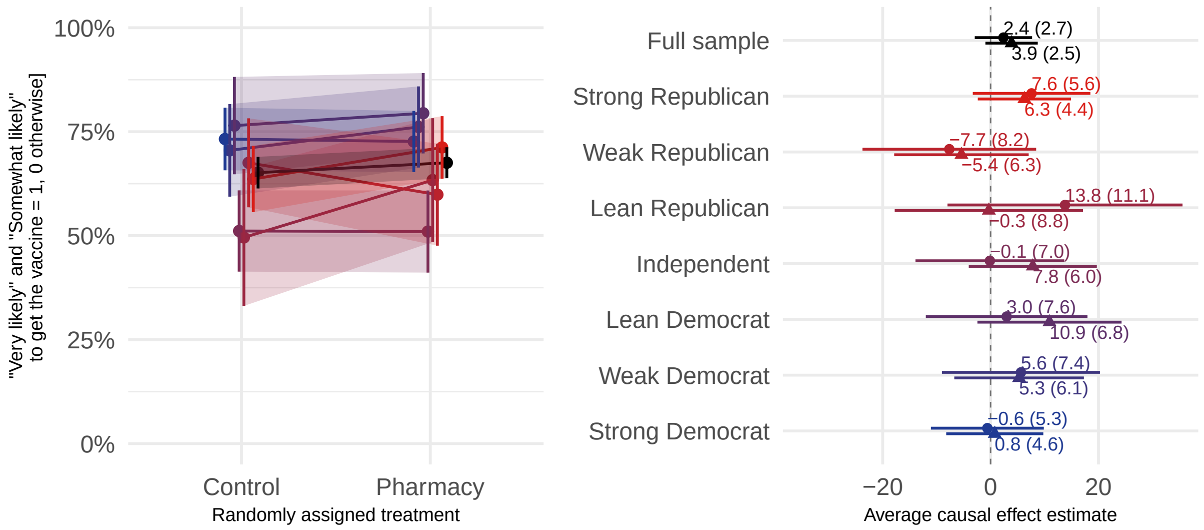

Figure S6: Endorsement experiment E-1: Insurance

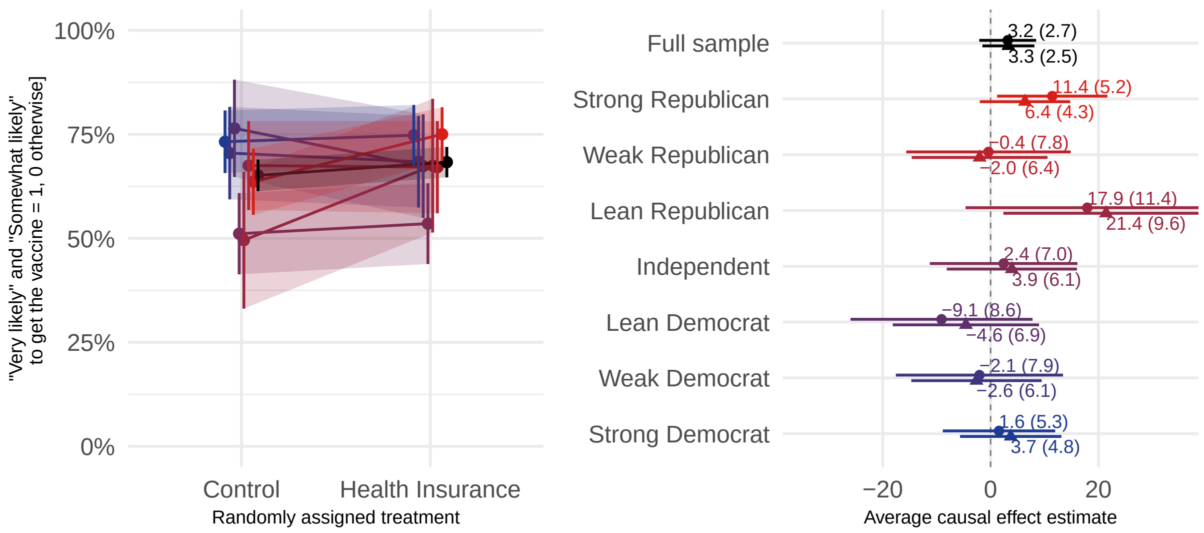

Figure S7: Endorsement experiment E-1: Spiritual Leader

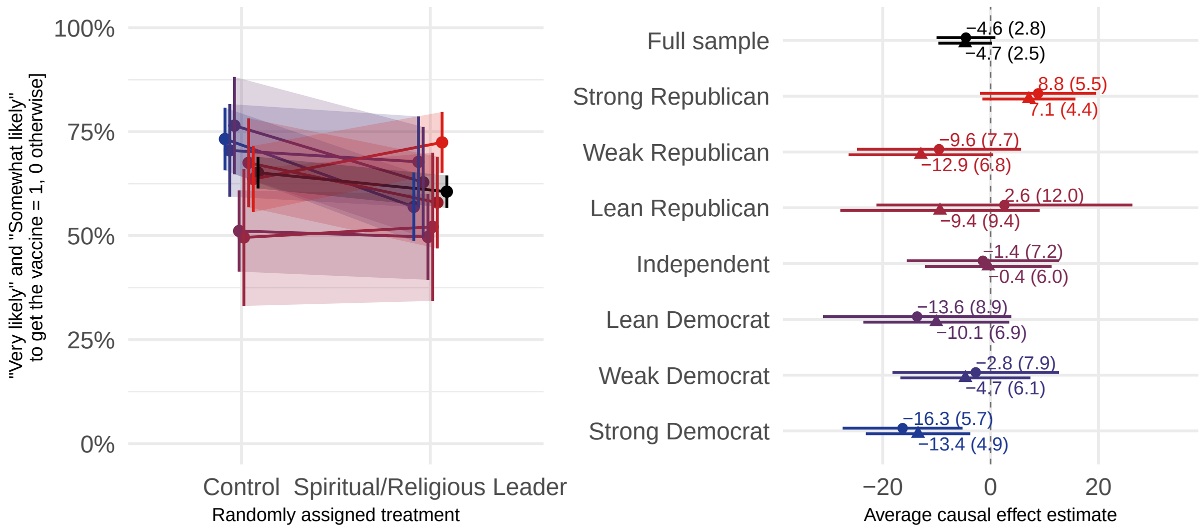

Figure S8: Endorsement experiment E-2: Trump

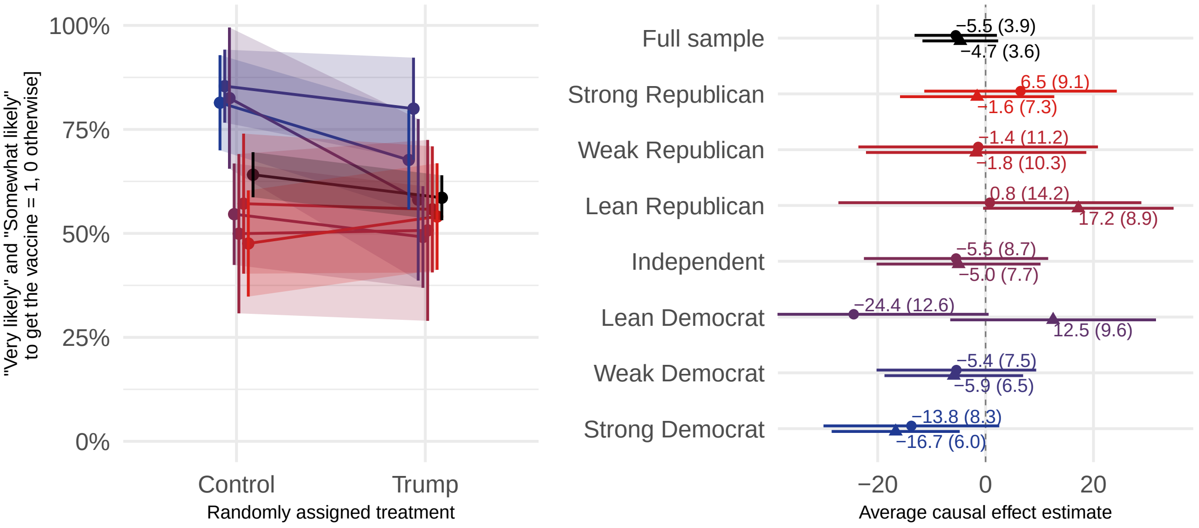

Figure S9: Endorsement experiment E-2: Trump and Fauci

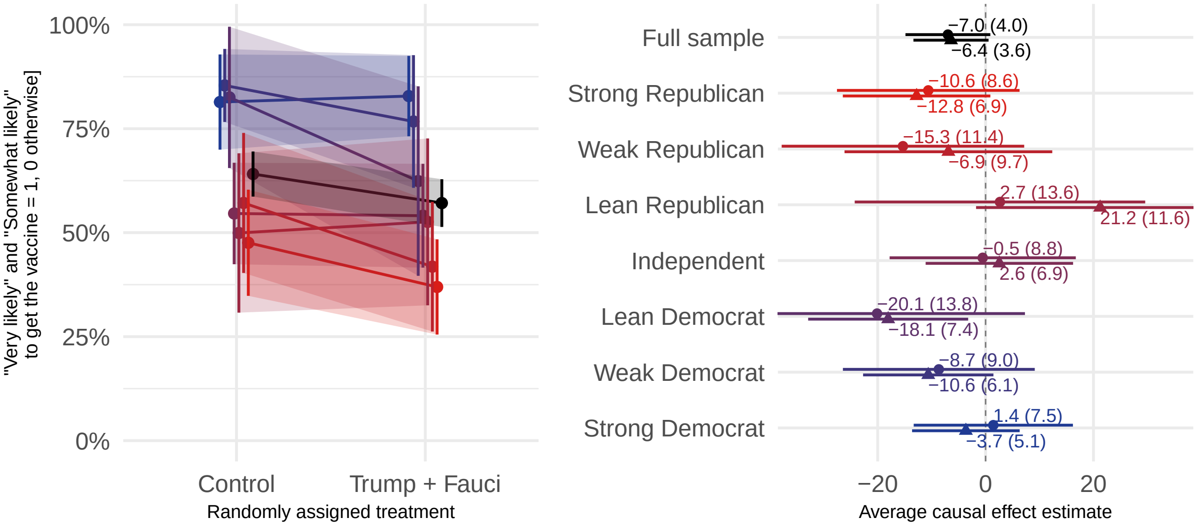

Figure S10: Endorsement experiment E-2: Fauci

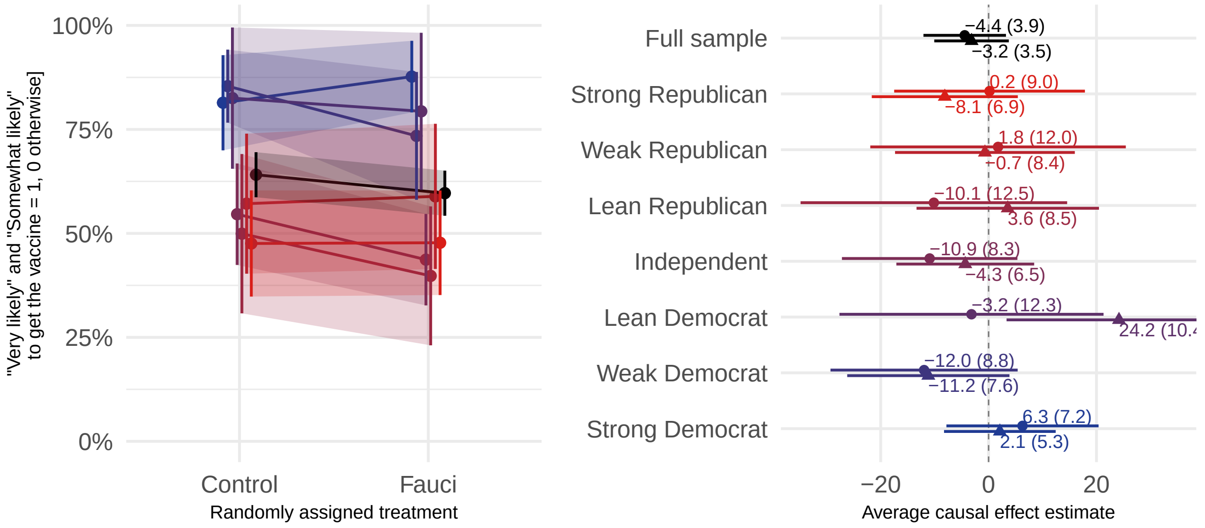

Figure S11: Endorsement experiment E-2: Biden and Fauci

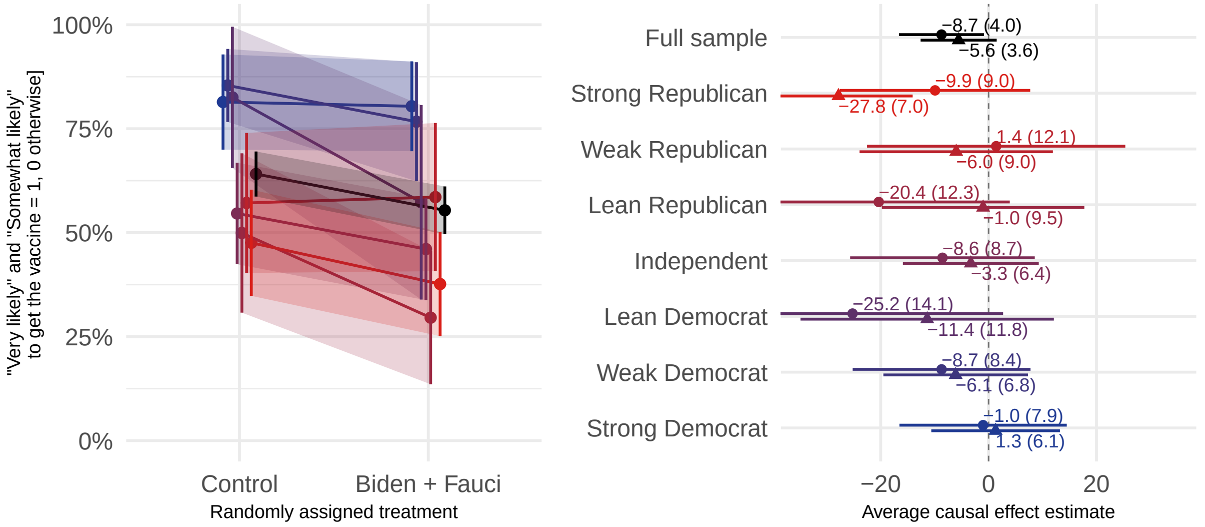

Figure S12: Endorsement experiment E-2: Biden

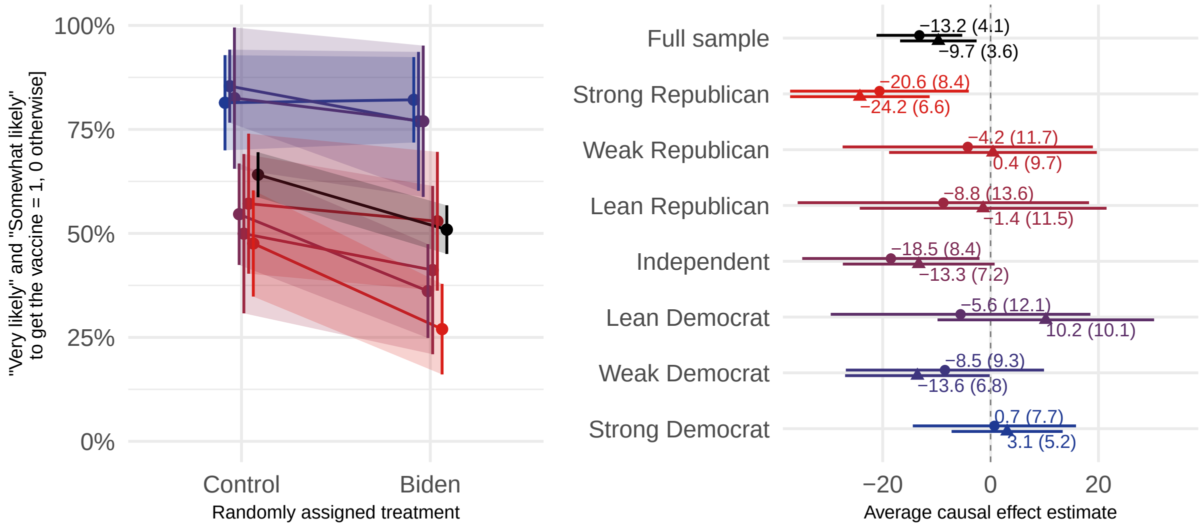

Figure S13: Endorsement experiment E-2: Obama

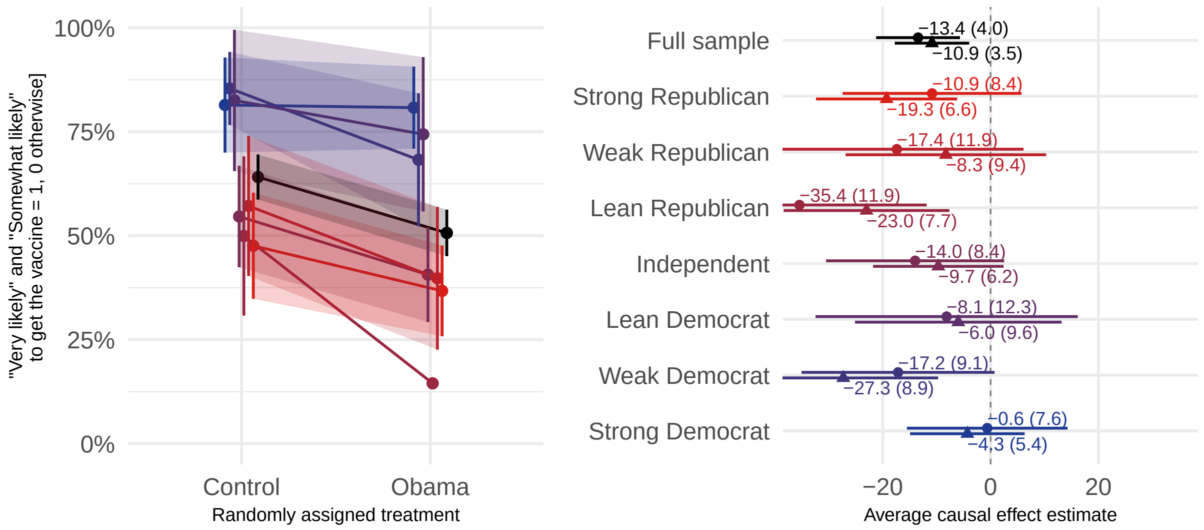

Figure S14: Endorsement experiment E-2: James

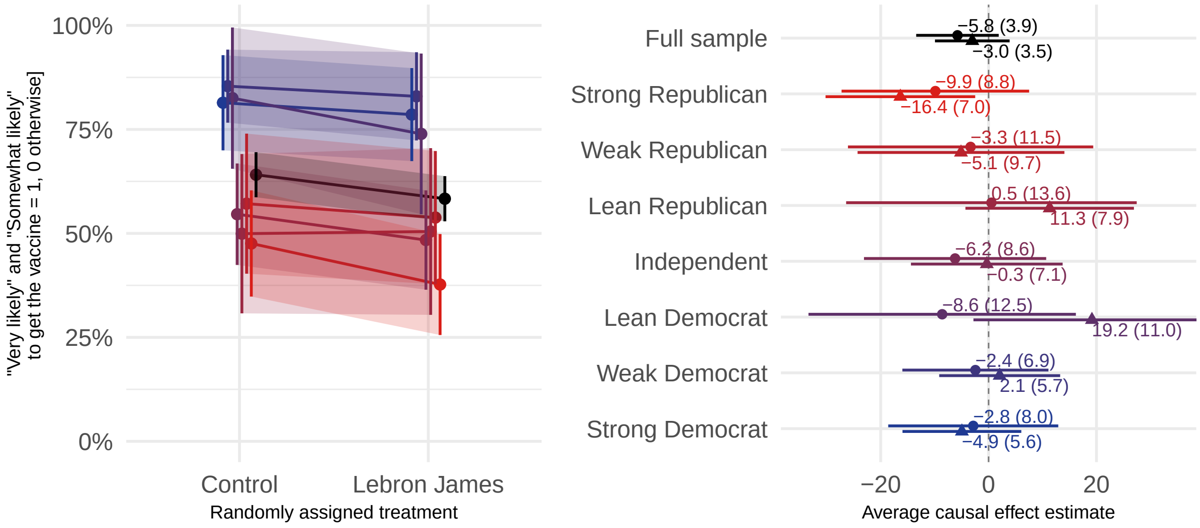

Figure S15: Endorsement experiment E-2: Ramos

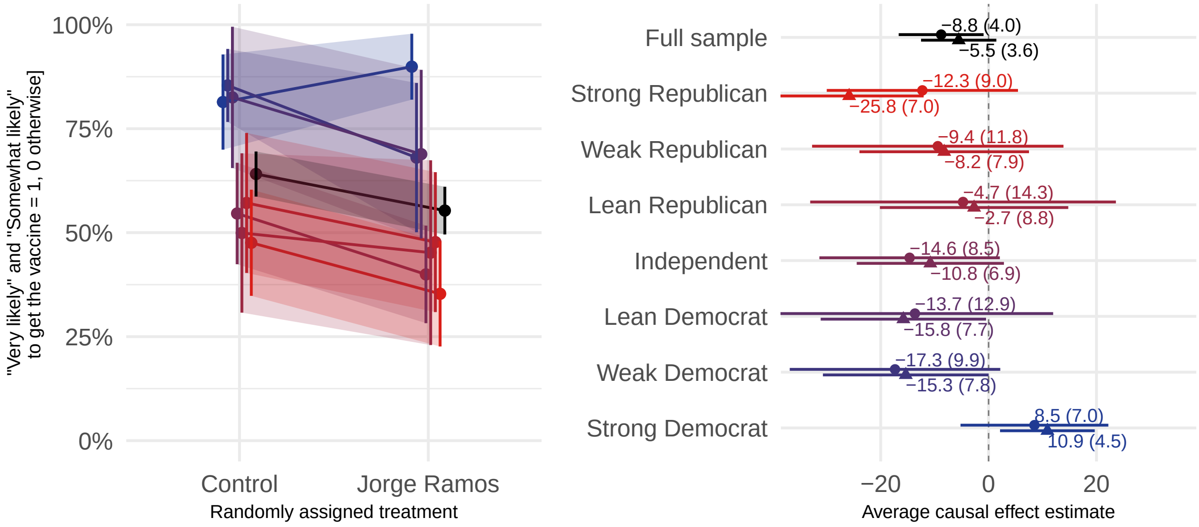

Figure S16: Guidance experiment G-1: Less restrictive mask guidance

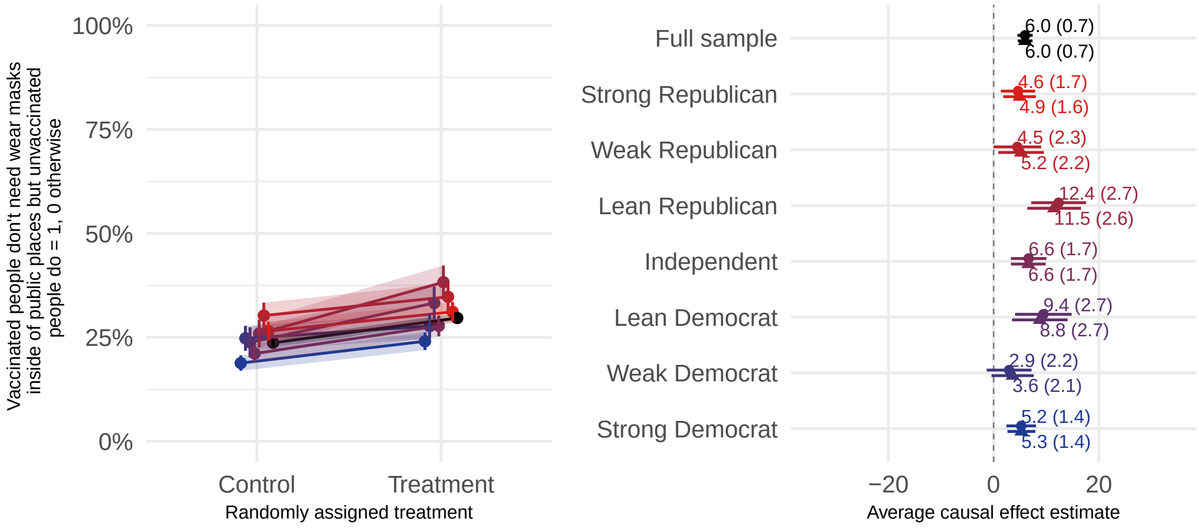

Figure S17: Guidance experiment G-3: More restrictive mask guidance

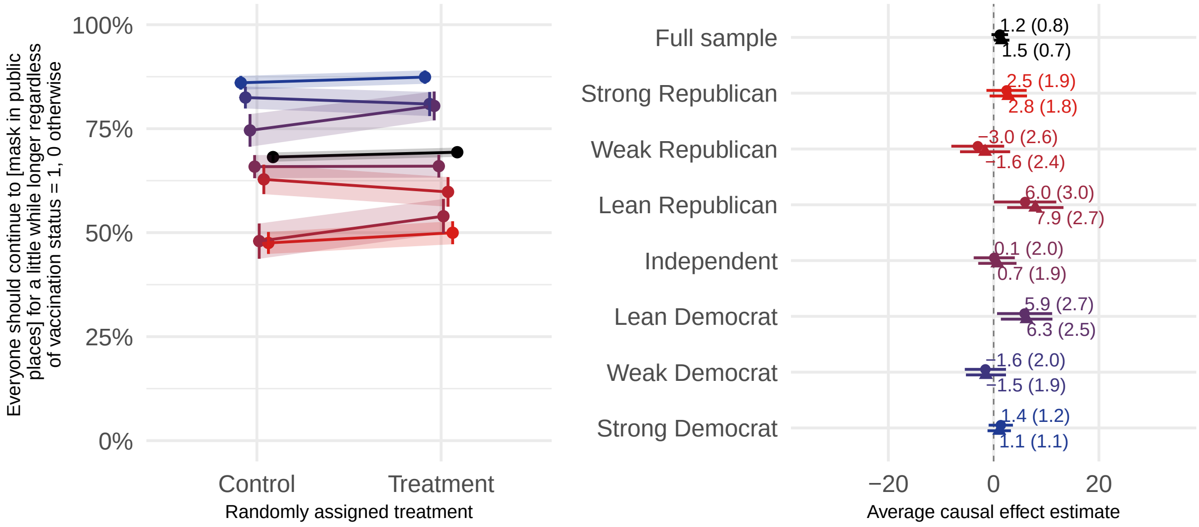

Figure S18: Information experiment I-1: Contagiousness

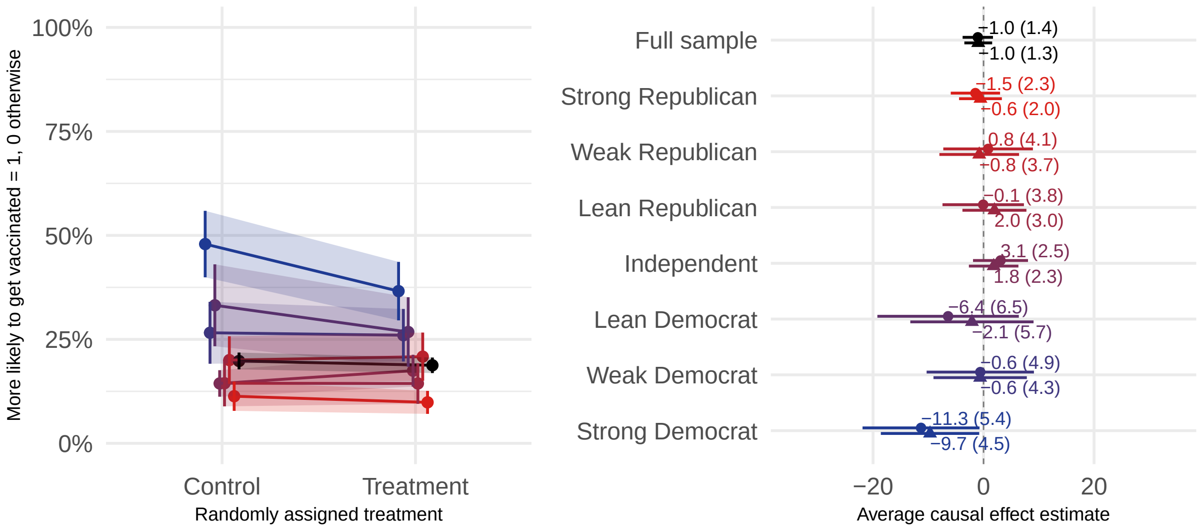

Figure S19: Information experiment I-2: Delta Variant

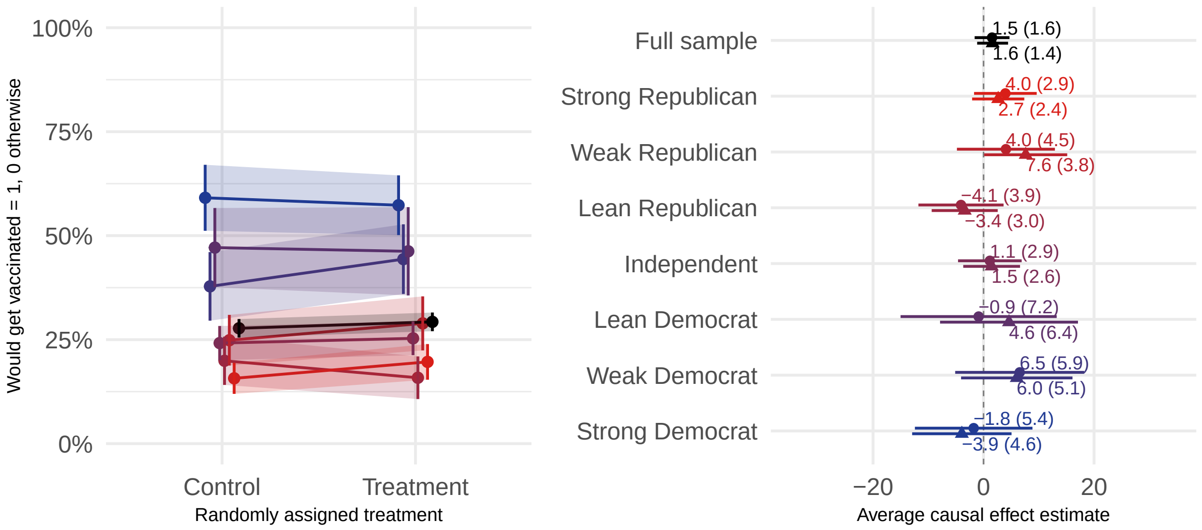

Figure S20: Information experiment I-3: Bivalent Booster - Adult

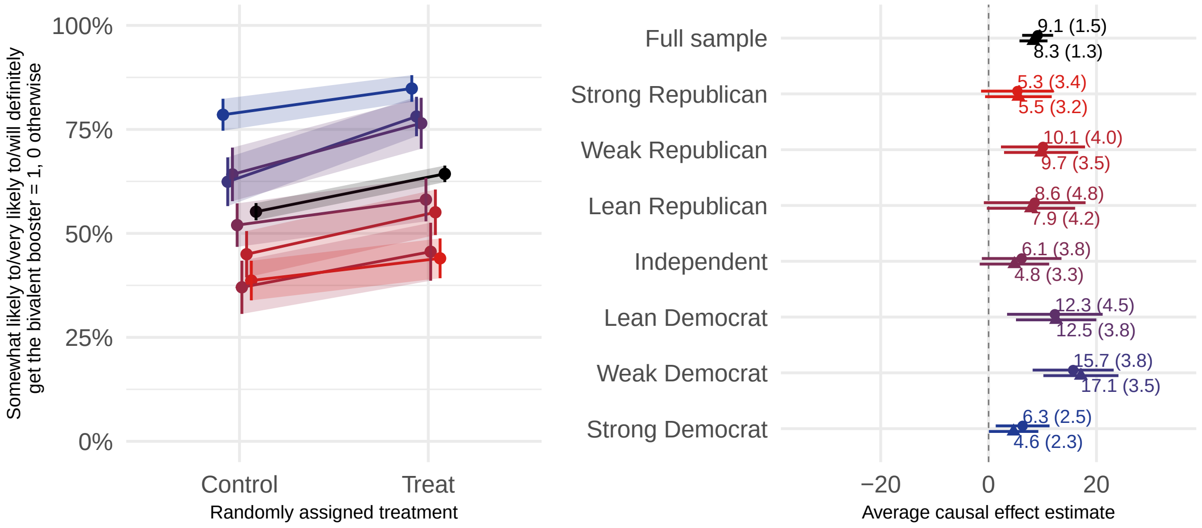

Figure S21: Information experiment I-4: Bivalent Booster - Child

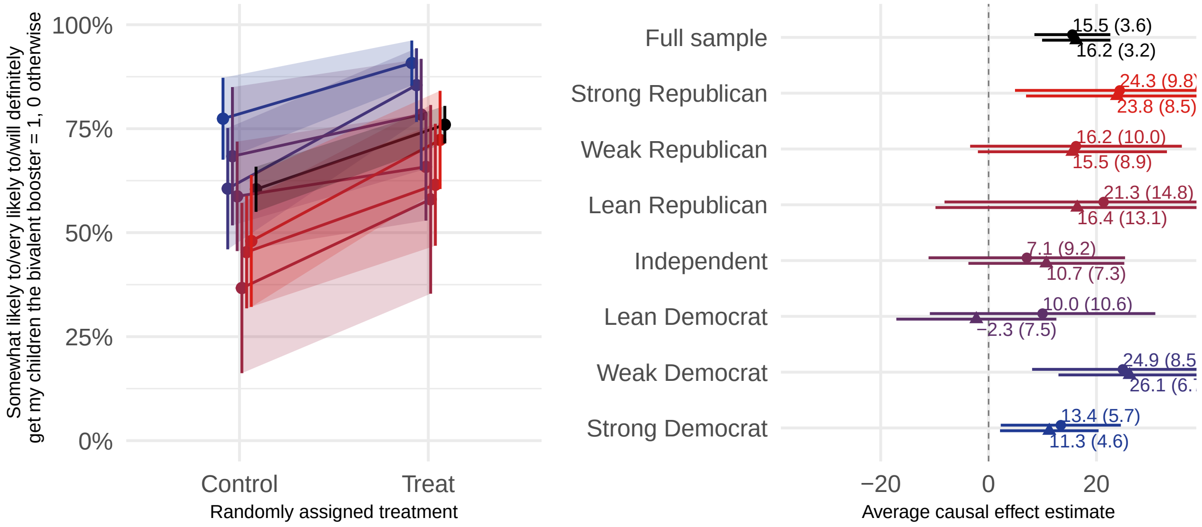

Figure S22: Information experiment I-5: Vaccince - Child

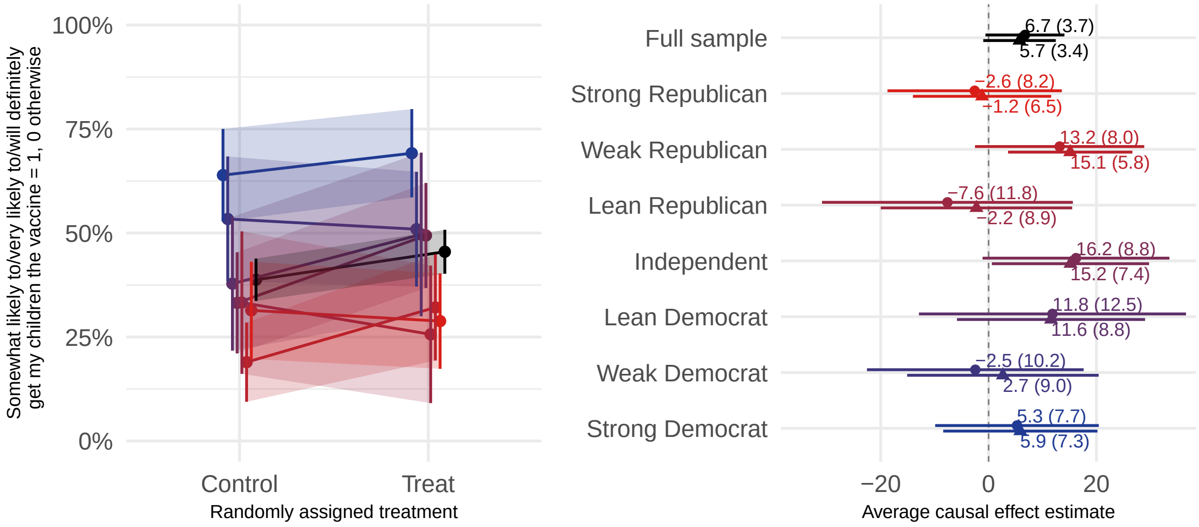

D Estimates among the "predicted to be unvaccinated"

The unvaccinated population changed over the course of our experiments. To add to our comparisons of experiments across time, we would like to know, for example, whether our 2020 survey participants end up vaccinated by our final survey wave in October 2022. We fit a model to the 29,448 final wave respondents whose ultimate vaccination status we do know. 8,738 (29.7%) of these people had not received a single shot at the time of the final wave survey: "never vaxxers."

We used variables in our model from questions which we asked on each prior wave of the survey. This allowed us to fit the model to training data (final wave) and make predictions on earlier wave datasets. The random forest model outperformed logistic regression and gradient boosted tree models. The out-of-bag error rate is 21.33%, and figure S23 shows the variables we included and the relative importance of those variables for model predictions. The mean decrease in accuracy on the y-axis is a measure of how much removing (permuting) one variable throws off predictions made by the model. People's answers to our flu vaccine question along with their party identification and age are relatively more predictive of their eventual COVID-19 vaccine status than health indicator questions (diabetes, blood pressure, etc).

Figure S23: Final Wave Vaccine Status Random Forest Variable Importance

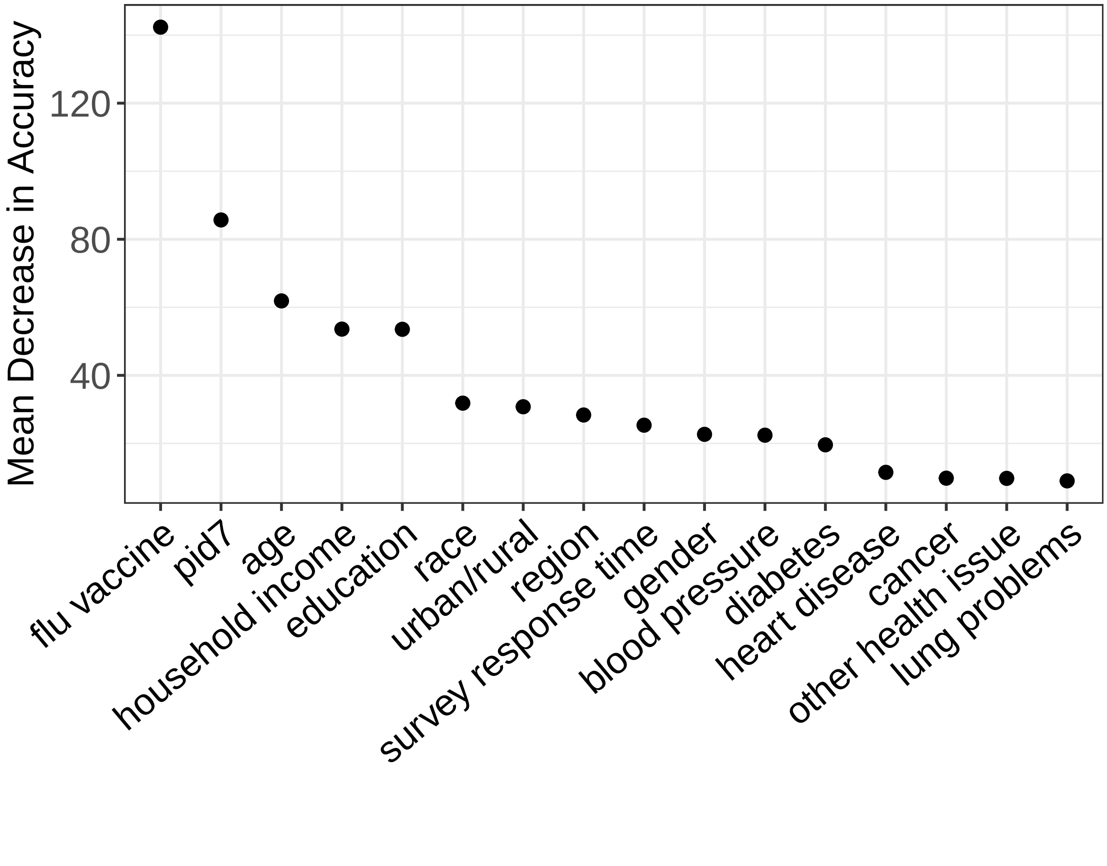

Figure S24: Endorsement experiment E-1: Trump among "never vaxxers"

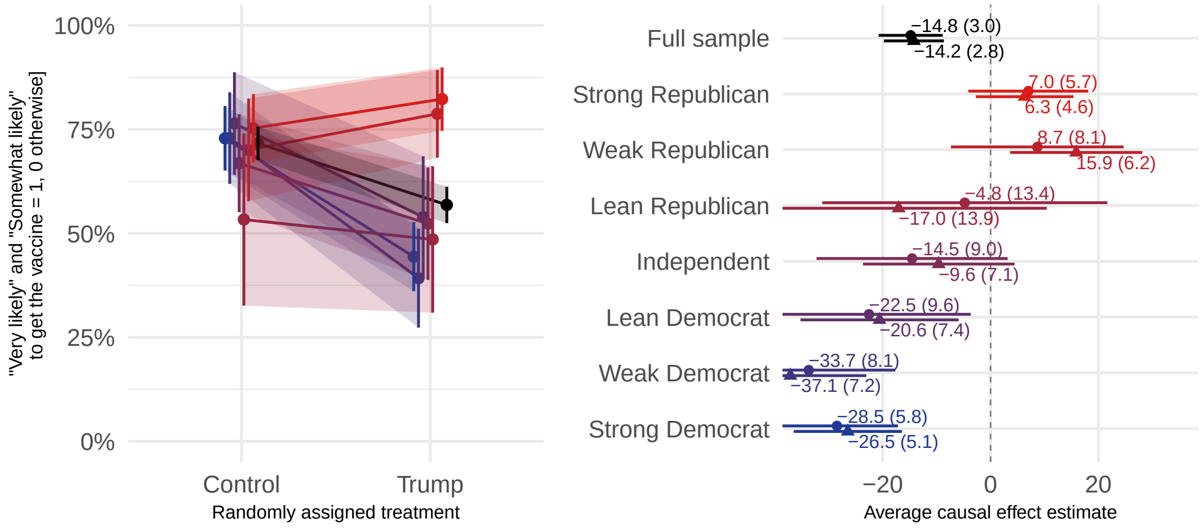

Figure S25: Endorsement experiment E-1: Fauci among "never vaxxers"

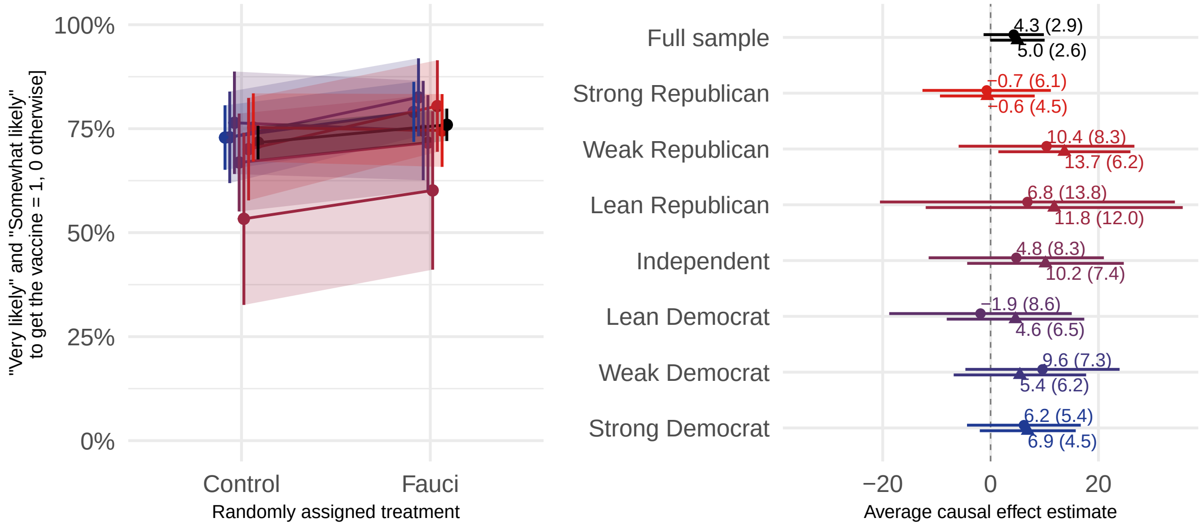

Figure S26: Endorsement experiment E-1: Trump and Fauci among “never vaxxers”

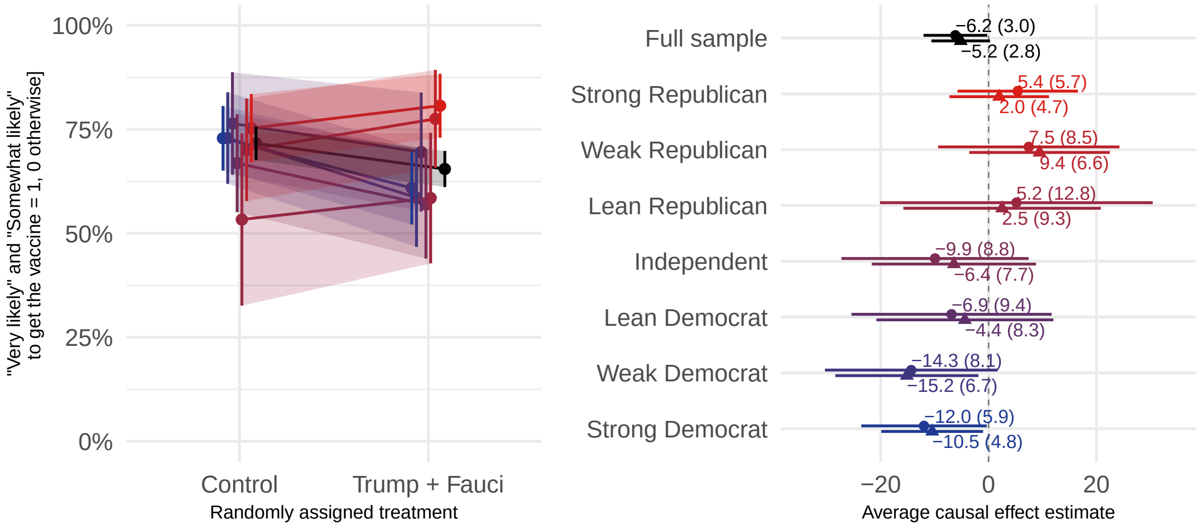

Figure S27: Endorsement experiment E-1: Physician among “never vaxxers”

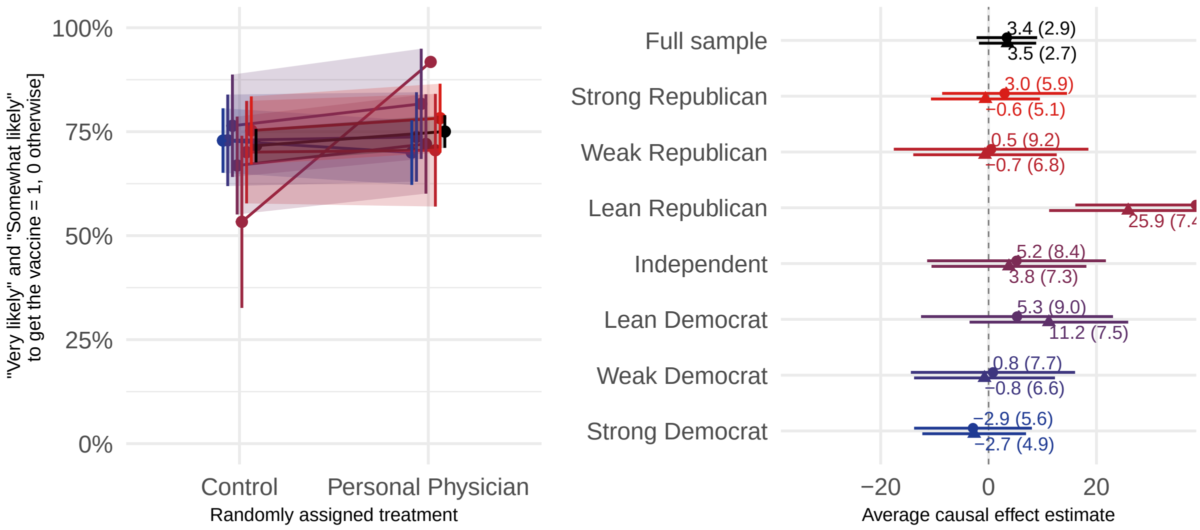

Figure S28: Endorsement experiment E-1: Pharmacy among "never vaxxers"

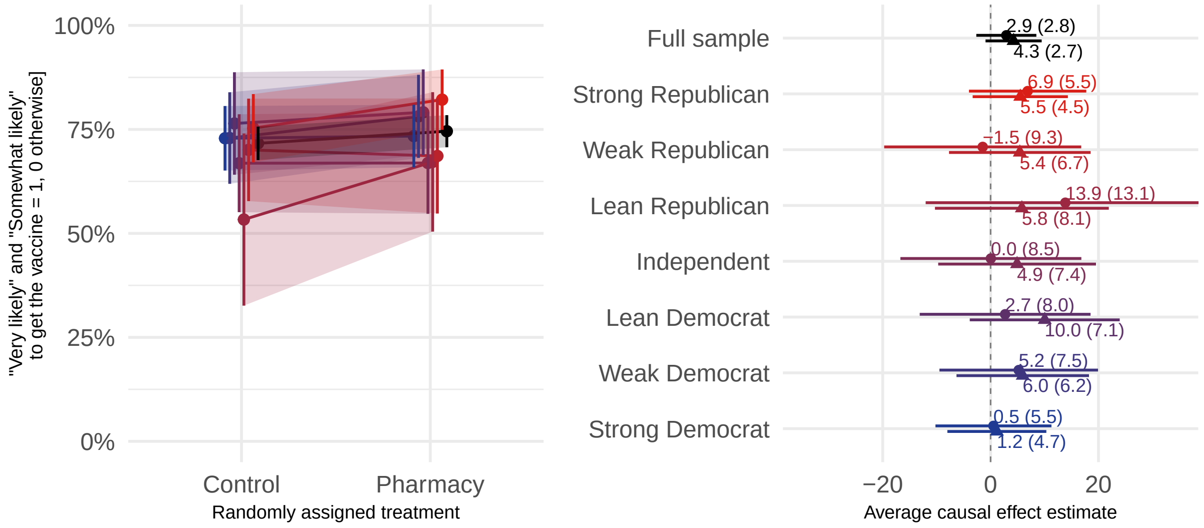

Figure S29: Endorsement experiment E-1: Insurance among "never vaxxers"

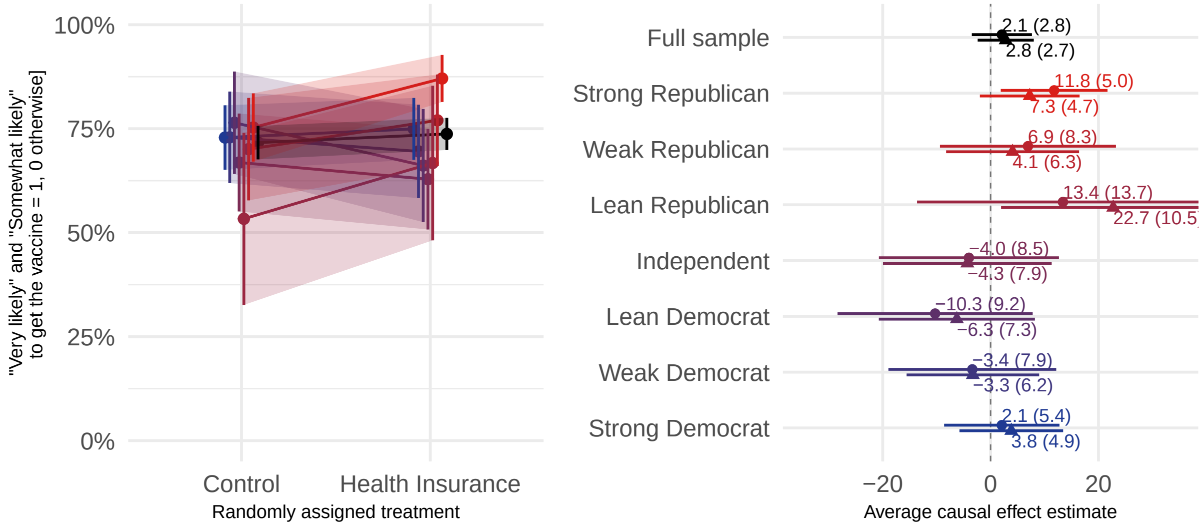

Figure S30: Endorsement experiment E-1: Spiritual Leader among “never vaxxers”

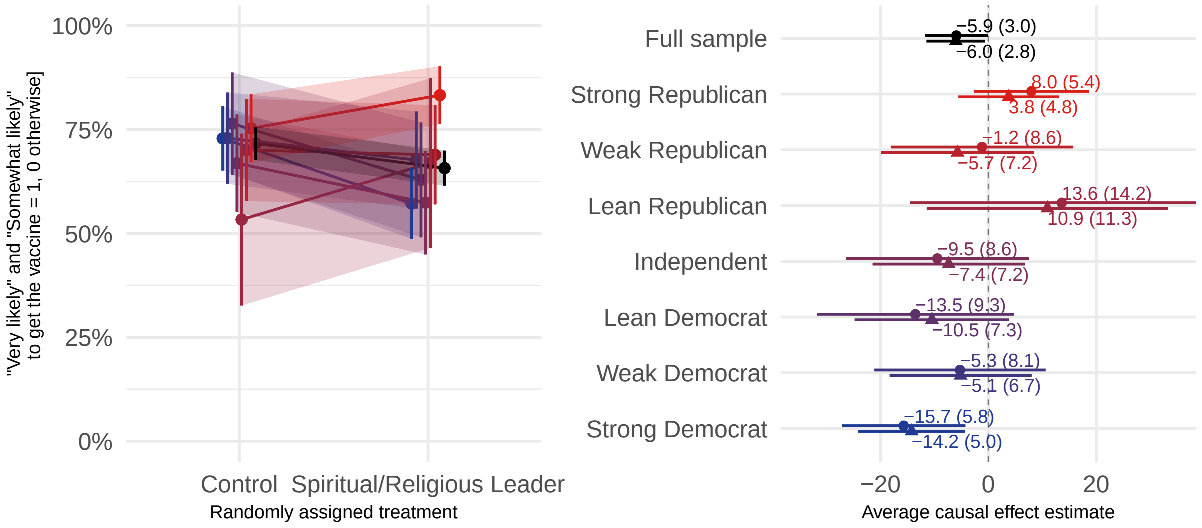

Figure S31: Endorsement experiment E-2: Trump among “never vaxxers”

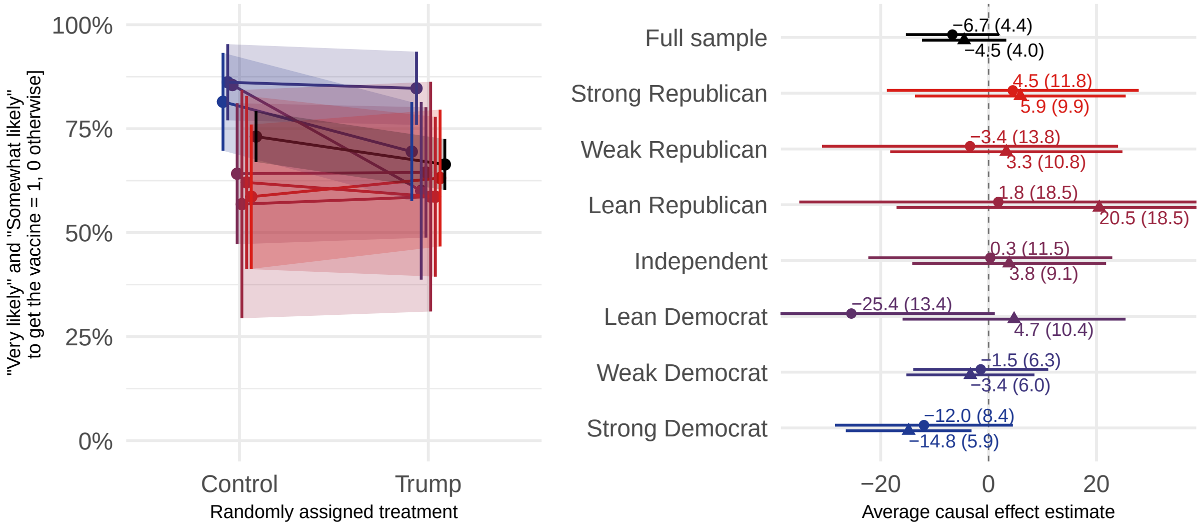

Figure S32: Endorsement experiment E-2: Trump and Fauci among "never vaxxers"

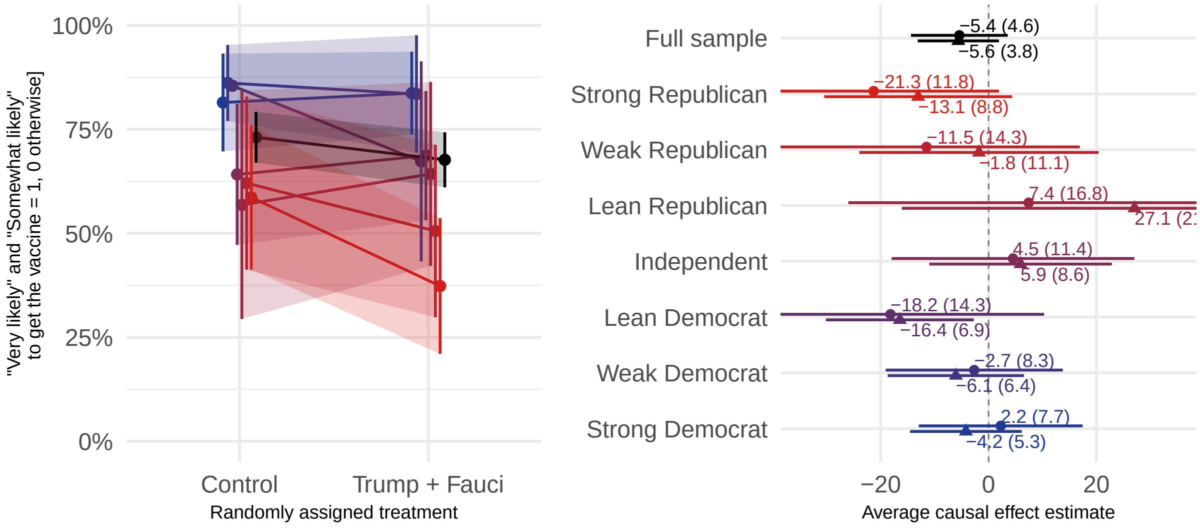

Figure S33: Endorsement experiment E-2: Fauci among "never vaxxers"

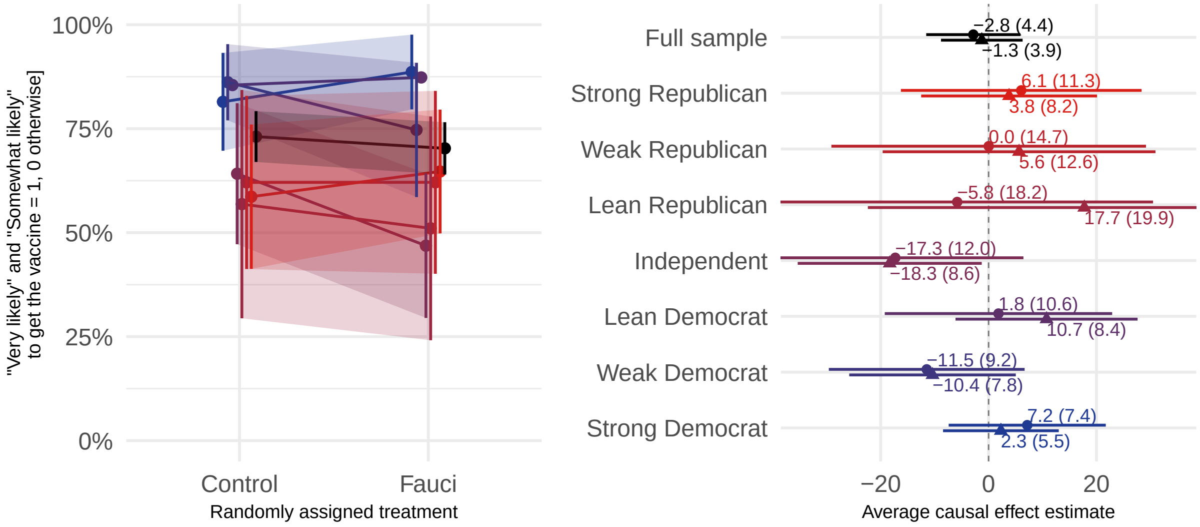

Figure S34: Endorsement experiment E-2: Biden and Fauci among “never vaxxers”

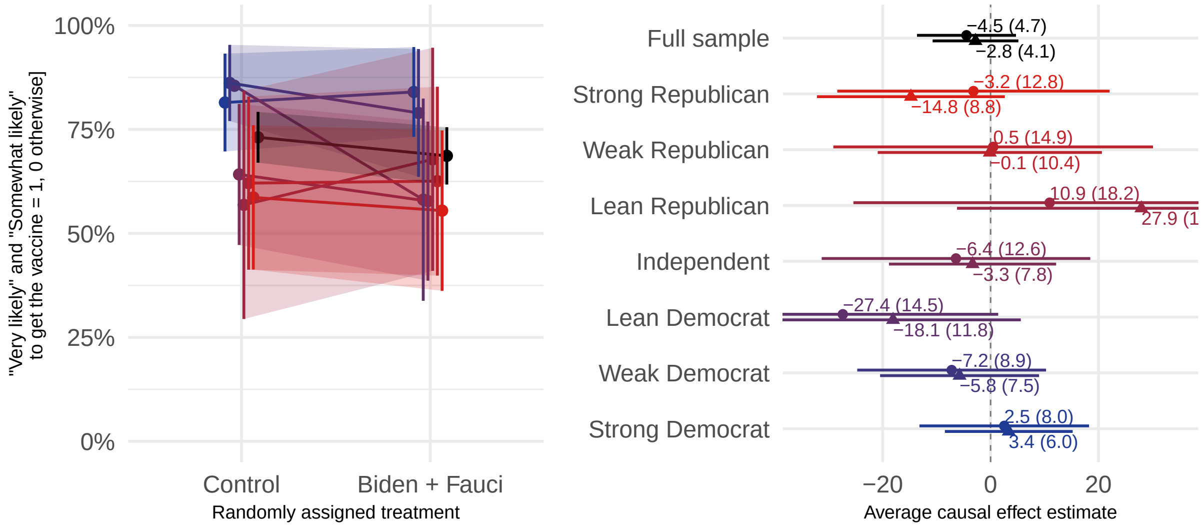

Figure S35: Endorsement experiment E-2: Biden among “never vaxxers”

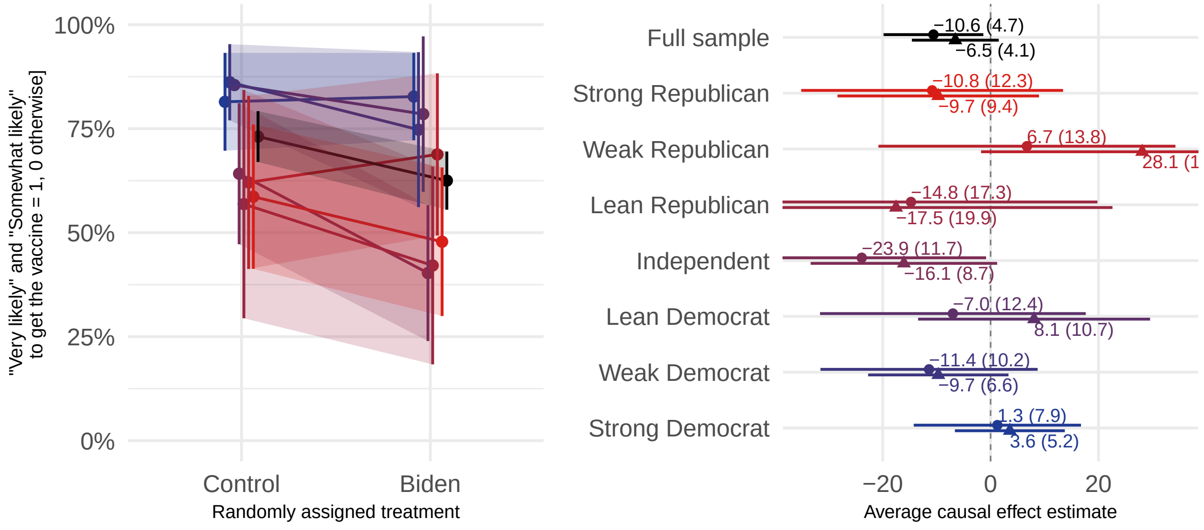

Figure S36: Endorsement experiment E-2: Obama among “never vaxxers”

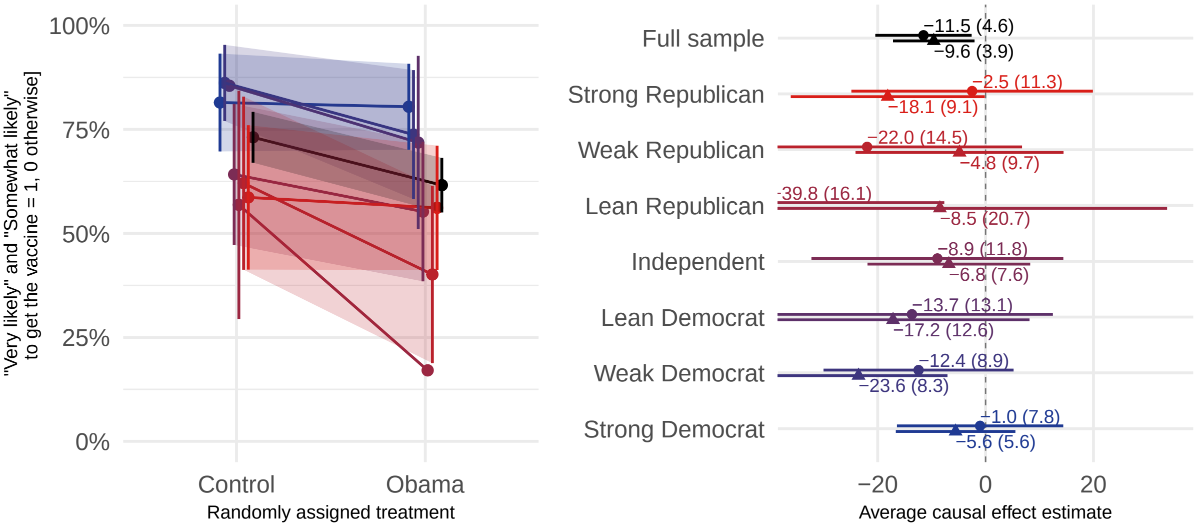

Figure S37: Endorsement experiment E-2: James among “never vaxxers”

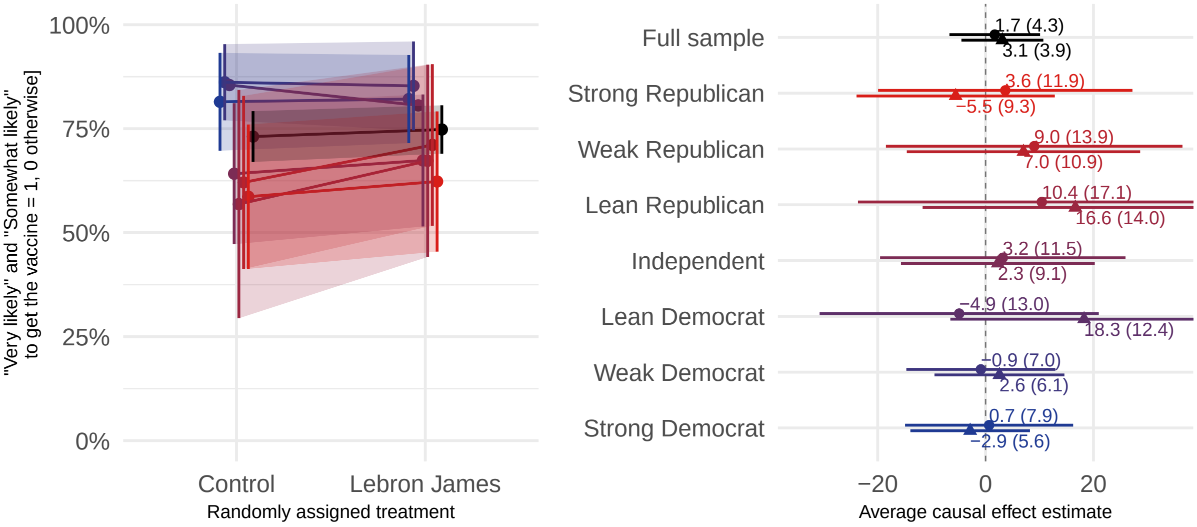

Figure S38: Endorsement experiment E-2: Ramos among "never vaxxers"

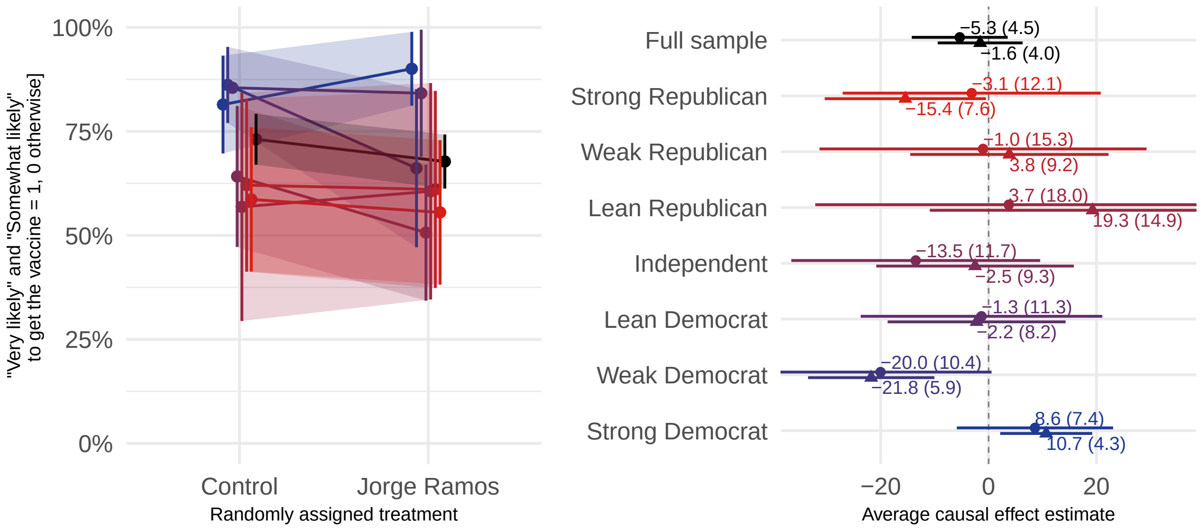

Figure S39: Guidance experiment G-1: Less restrictive mask guidance among "never vaxxers"

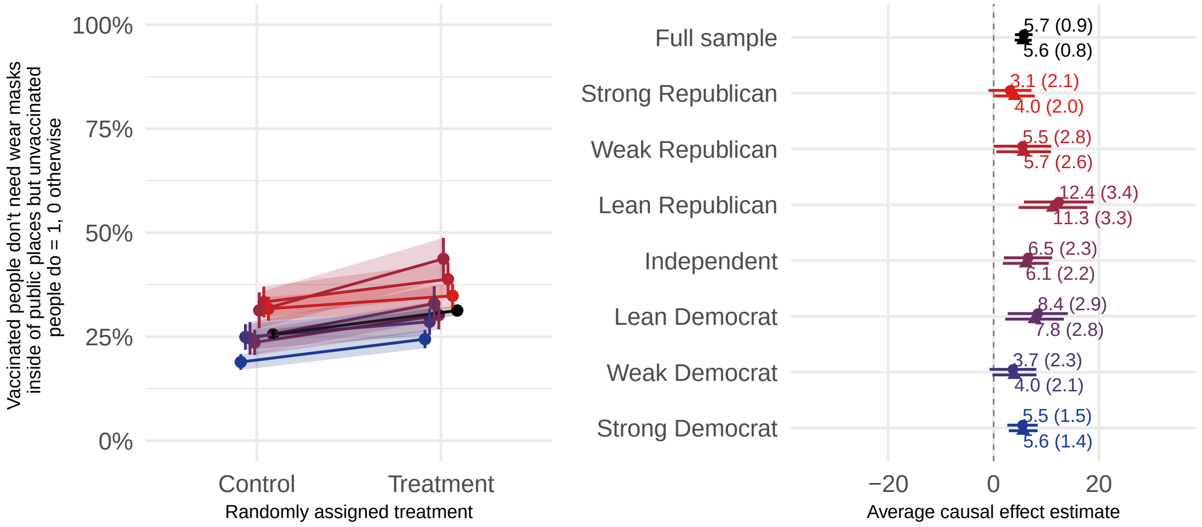

Figure S40: Guidance experiment G-3: More restrictive mask guidance among "never vaxxers"

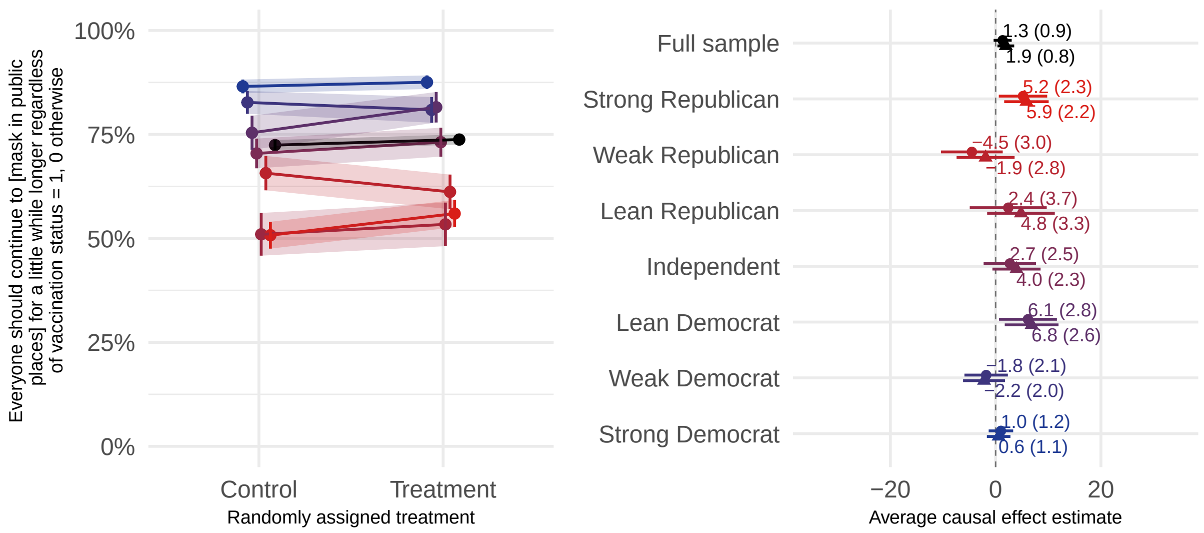

Figure S41: Information experiment I-1: Contagiousness among "never vaxxers"

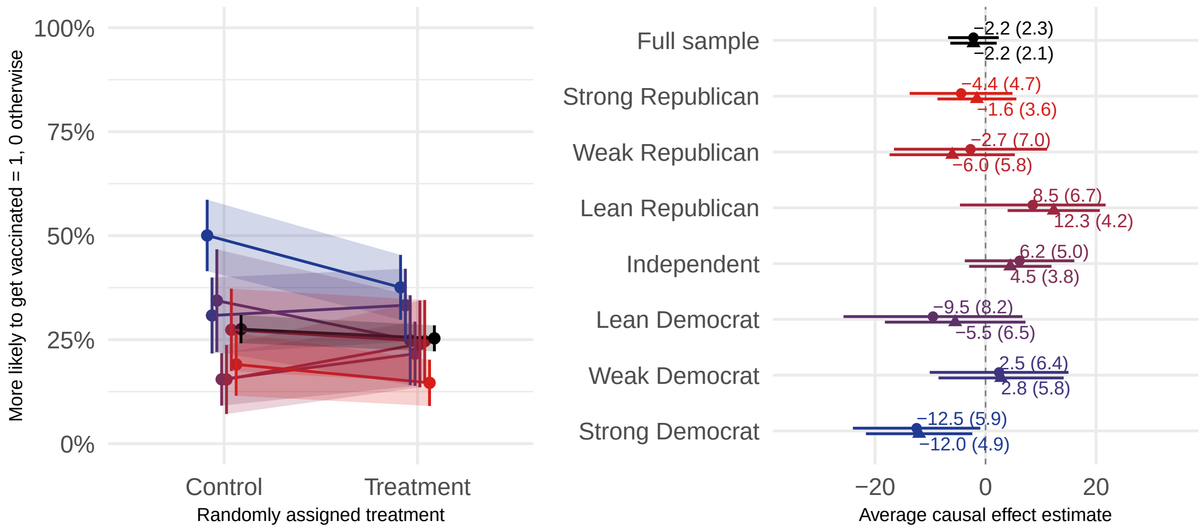

Figure S42: Information experiment I-2: Delta Variant among "never vaxxers"

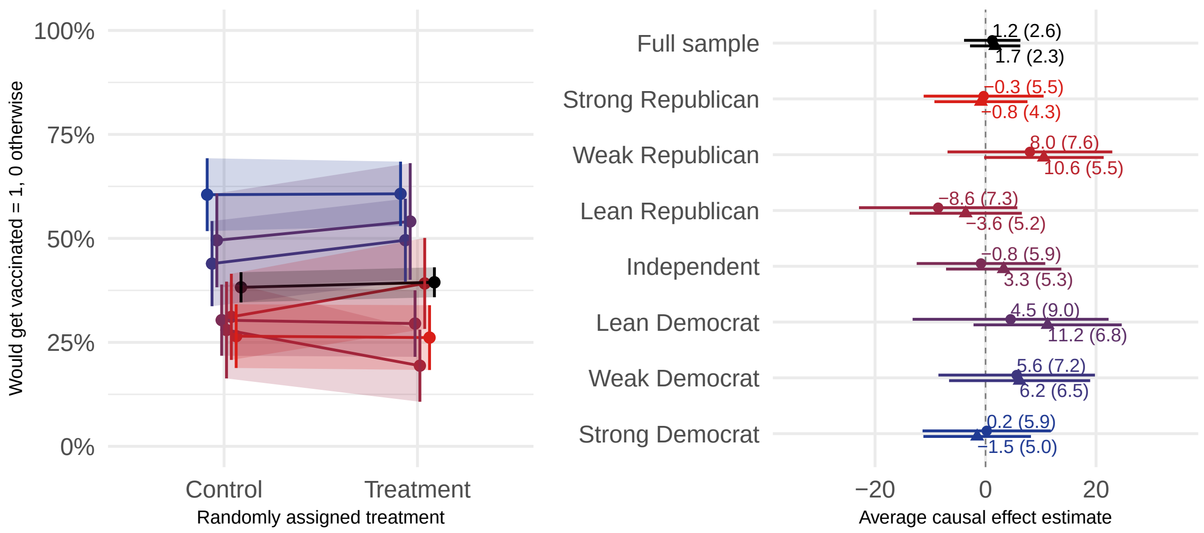

Figure S43: Information experiment I-3: Bivalent Booster - Adult among "never vaxxers"

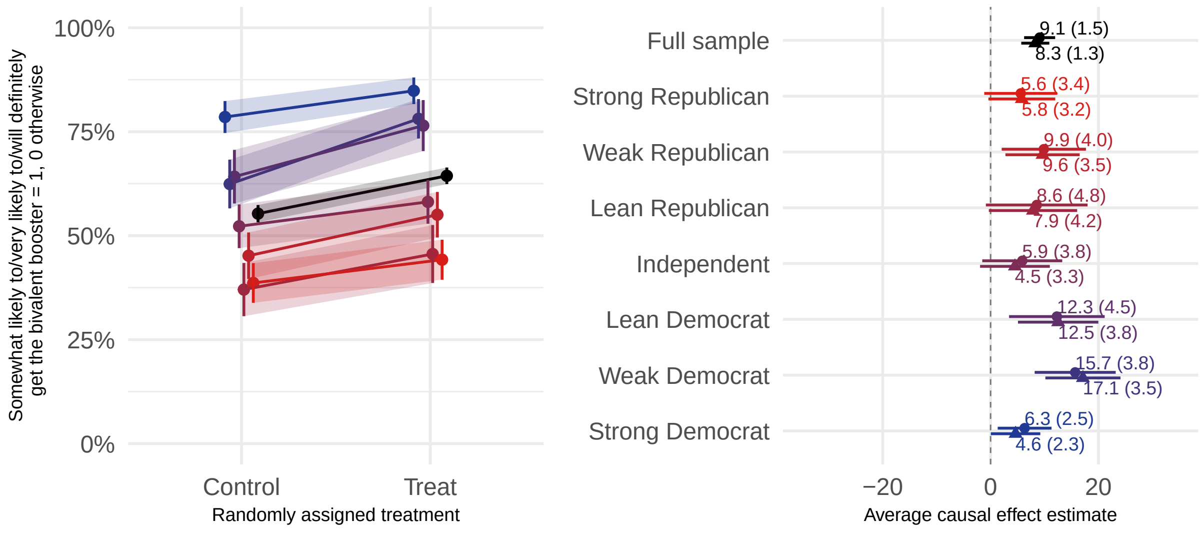

Figure S44: Information experiment I-4: Bivalent Booster - Child among "never vaxxers"

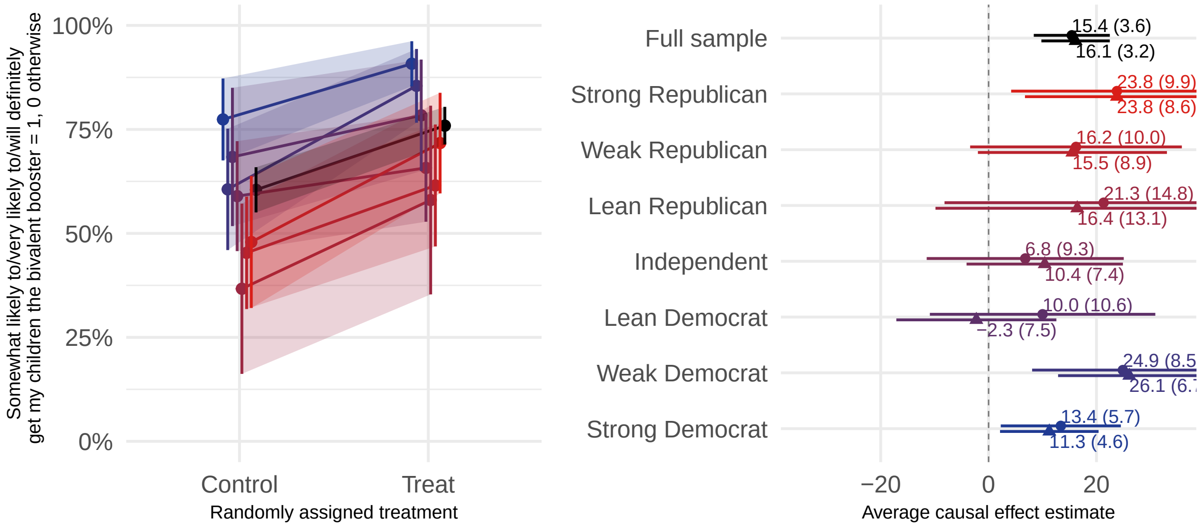

Figure S45: Information experiment I-5: Vaccince - Child among "never vaxxers"

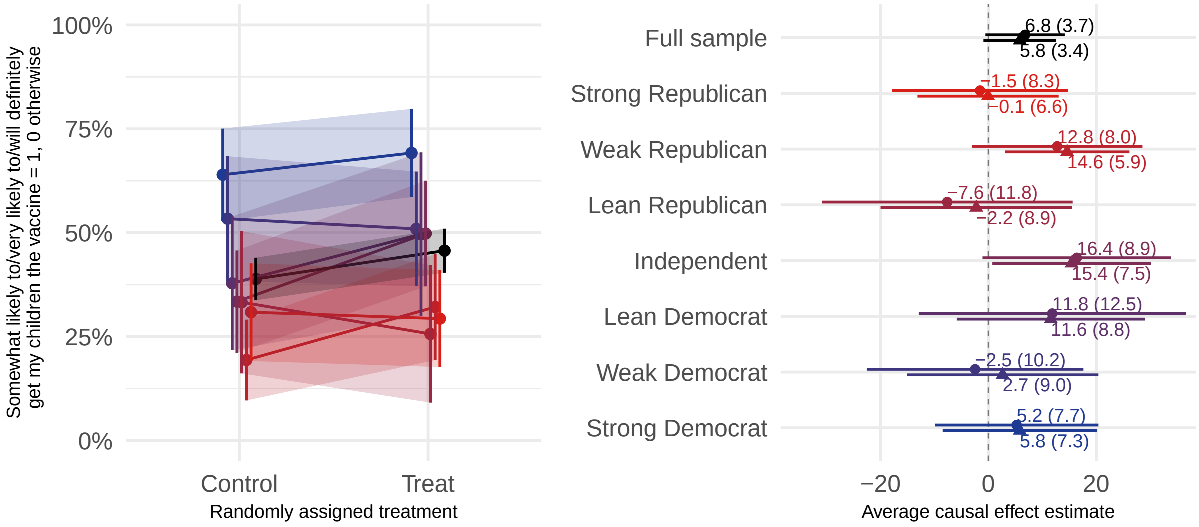

E Additional figures and analysis

E.1 General Analyses

Figure S46 compares the ATE and Treatment x Party ID interaction terms across weighted and unweighted models. The weighted models target population-level estimands (where the population is defined by the 2017 American Community Survey of the U.S. Census Bureau) and the unweighted models target sample-level estimands. A concern is that weighted estimates are less precise than unweighted estimates. Indeed that it is what we find: the weighted standard errors are on average 1.6 times their unweighted counterparts. That said, the signs and significances of our estimates match in most (51 of 60) cases.

There are five estimates in which only the unweighted estimate is significant: the ATE for

E-1: Spiritual leader, G-2: Concert, Pooled and the interaction terms for E-1: Trump and Fauci, E-1: Spiritual leader, and E-2: Trump. There are two estimates for which only the weighted estimate is significant: the ATE and interaction term for G-2: Team, Pooled.

We present weighted estimates in the main text because our estimands are defined at the population level and we are willing to pay the precision penalty to maintain our interest in the population-level estimands. That said, the unweighted analysis provides even further evidence against strong heterogeneity in the effects of the guidance and information experiments and also further evidence that Trump endorsements are polarizing.

Figure S46: Comparing weighted and unweighted estimates

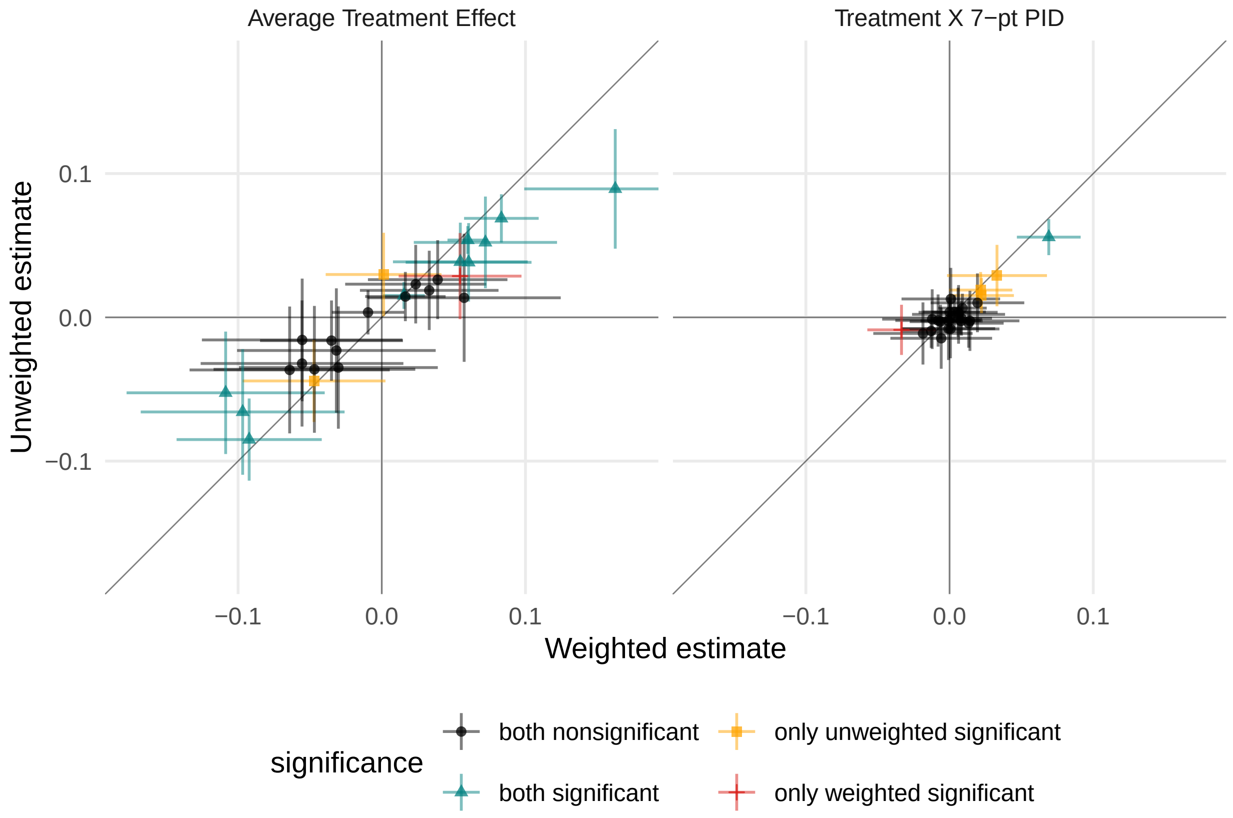

E.2 Endorsement Experiments

Wave 3 Endorsement Experiment CATEs, Difference-in-CATEs, and Equivalence Tests

Endorser	CATEs		DIC	Equivalence Tests	
-	Dem	GOP	GOP - Dem	5pp	10pp
Trump	-0.266 (0.039) *	0.103 (0.038) *	0.369 (0.053) *	1.000	1.000
Fauci	0.062 (0.034)	0.042 (0.036)	-0.020 (0.052)	0.282	0.061
Trump + Fauci	-0.104 (0.039) *	0.029 (0.037)	0.133 (0.053) *	0.942	0.733
Spiritual	-0.095 (0.038) *	0.012 (0.038)	0.107 (0.053) *	0.858	0.553
Health Insurance	0.008 (0.036)	0.071 (0.037)	0.063 (0.052)	0.596	0.236
Pharmacy	0.031 (0.035)	0.038 (0.037)	0.006 (0.052)	0.200	0.035 *
Personal Physician	0.001 (0.037)	0.045 (0.038)	0.045 (0.053)	0.460	0.149

Wave 5 Endorsement Experiment CATEs, Difference-in-CATEs, and Equivalence Tests

Endorser	CATEs		DIC	Equivalence Tests	
-	Dem	GOP	GOP - Dem	5pp	10pp
Trump	-0.114 (0.048) *	0.018 (0.056)	0.132 (0.079)	0.850	0.657
Fauci	0.018 (0.047)	-0.035 (0.058)	-0.053 (0.082)	0.515	0.283
Trump + Fauci	-0.098 (0.046) *	-0.053 (0.057)	0.044 (0.081)	0.473	0.245
Obama	-0.088 (0.049)	-0.173 (0.053) *	-0.085 (0.074)	0.683	0.422
Biden	-0.005 (0.046)	-0.127 (0.056) *	-0.122 (0.079)	0.819	0.610
Biden + Fauci	-0.052 (0.050)	-0.126 (0.057) *	-0.074 (0.081)	0.617	0.374
Ramos	-0.017 (0.046)	-0.099 (0.057)	-0.082 (0.080)	0.656	0.413
James	-0.019 (0.043)	-0.073 (0.057)	-0.055 (0.081)	0.524	0.288

E.3 Guidance Experiments

Figure S47 evaluates the three option outcome distribution for each guidance experiment instead of collapsing it to binary based on which recommendation is CDC-backed at the time. χ^2 tests show the distributions are significantly different between treatment and control groups.

Figure S47: χ^2 Test for G-1 and G-3

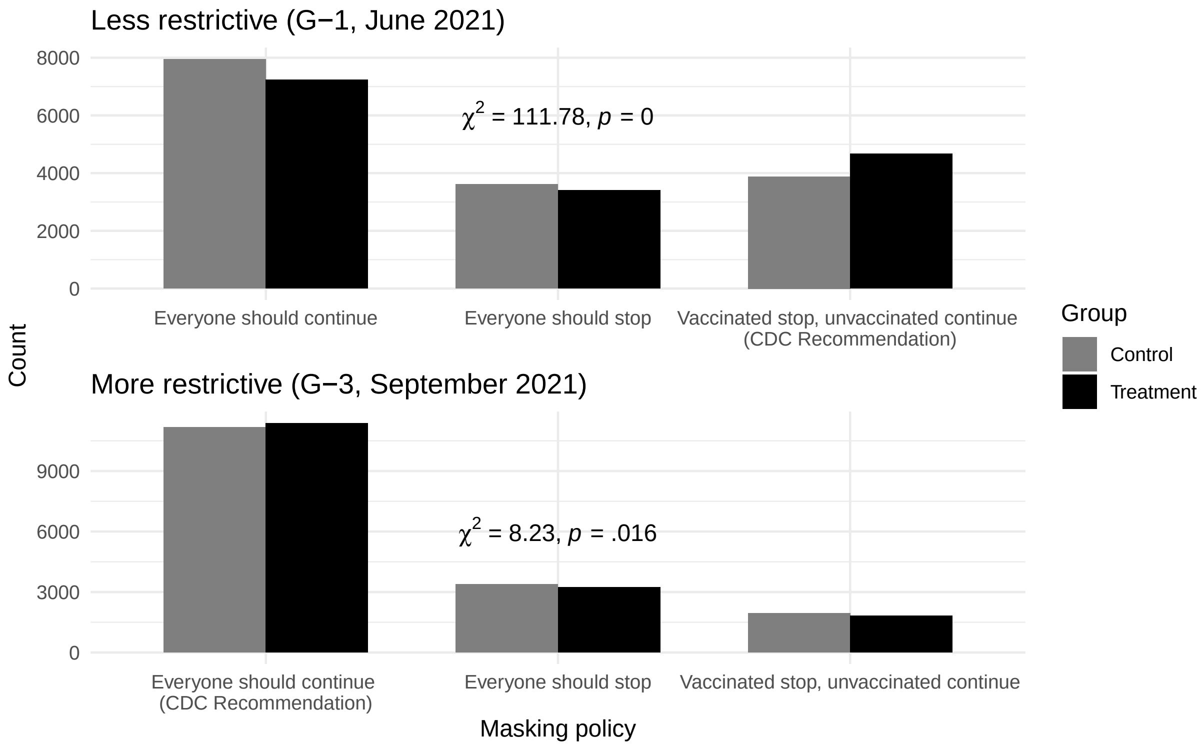

Table S4: Mandate Experiment

	Concert	Restaurant	Team	Trip
(Intercept)	0.188*	0.154*	0.179*	0.228*
	(0.015)	(0.015)	(0.015)	(0.018)
Treatment	0.001	0.060*	0.054*	0.072*
	(0.021)	(0.022)	(0.022)	(0.025)
Party ID (7-Point)	−0.053*	−0.033*	−0.032*	−0.027*
	(0.010)	(0.010)	(0.008)	(0.011)
Treatment x Party ID	0.013	0.006	−0.033*	−0.008
	(0.012)	(0.014)	(0.012)	(0.015)
Num.Obs.	2553	2684	2458	2580
R2	0.215	0.153	0.174	0.129
covariates	yes	yes	yes	yes

* $p < 0.05$
(F) and (S) indicate the "friend" and "solo" versions of the vignettes.

Guidance Experiments CATEs, Difference-in-CATEs, and Equivalence Tests

Experiment	CATEs		DIC	Equivalence Tests	
-	Dem	GOP	GOP - Dem	5pp	10pp
G-1: CDC Mask Guidance 1	0.015 (0.009)	0.028 (0.013) *	0.013 (0.018)	0.022 *	0.000 *
G-3: CDC Mask Guidance 2	0.054 (0.011) *	0.064 (0.012) *	0.010 (0.016)	0.007 *	0.000 *

G-2: Vaccine Mandate Vignette Experiment CATEs, Difference-in-CATEs, and Equivalence Tests

Experiment	CATEs		DIC	Equivalence Tests	
-	Dem	GOP	GOP - Dem	5pp	10pp
Concert (Friend)	0.007 (0.063)	-0.015 (0.034)	-0.021 (0.048)	0.274	0.050
Restaurant (Friend)	0.076 (0.063)	0.029 (0.045)	-0.047 (0.064)	0.481	0.203
Team (Friend)	0.139 (0.074)	-0.005 (0.034)	-0.144 (0.048) *	0.975	0.819
Trip (Friend)	0.176 (0.065) *	0.048 (0.044)	-0.128 (0.062) *	0.897	0.675
Concert (Self)	-0.058 (0.054)	-0.007 (0.030)	0.051 (0.043)	0.508	0.126
Restaurant (Self)	0.072 (0.065)	0.073 (0.030) *	0.001 (0.042)	0.122	0.009 *
Team (Self)	0.036 (0.063)	0.018 (0.029)	-0.017 (0.041)	0.214	0.022 *
Trip (Self)	0.057 (0.072)	0.074 (0.044)	0.017 (0.062)	0.295	0.090
Concert (Pooled)	-0.046 (0.045)	-0.007 (0.024)	0.039 (0.034)	0.375	0.038 *
Restaurant (Pooled)	0.052 (0.047)	0.046 (0.028)	-0.006 (0.039)	0.128	0.008 *
Team (Pooled)	0.130 (0.049) *	-0.013 (0.023)	-0.144 (0.032) *	0.998	0.913
Trip (Pooled)	0.130 (0.052) *	0.058 (0.034)	-0.072 (0.048)	0.672	0.278

Figure S48: Effects of Trip Mandate (Pooled) on Intentions to Vaccinate (June 2021, experiment G-2)

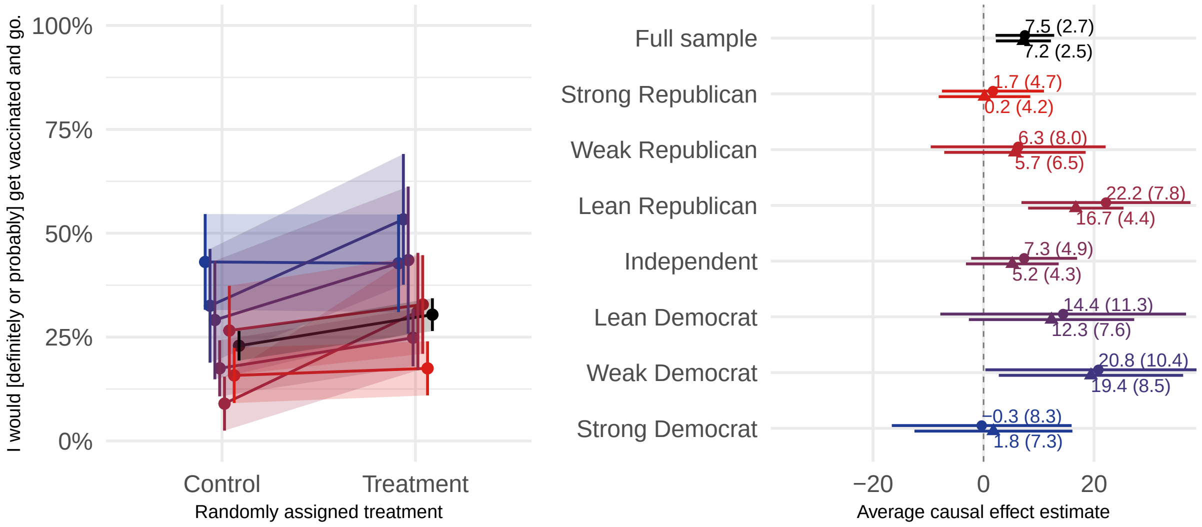

NOTE: Plotting symbols on the right-side panel represent the conditional average treatment effects within each row. Circles show the effects without covariates. Triangles represent the estimates from a model with covariates as discussed earlier. Lines on the left-side panel represent the average effects overall (black line) and for each level of partisanship (red for Republicans and blue for Democrats). Shades of the lines increase with increasing partisan intensity.

Figure S49: Effects of Trip Mandate (Friend) on Intentions to Vaccinate (June 2021, experiment G-2)

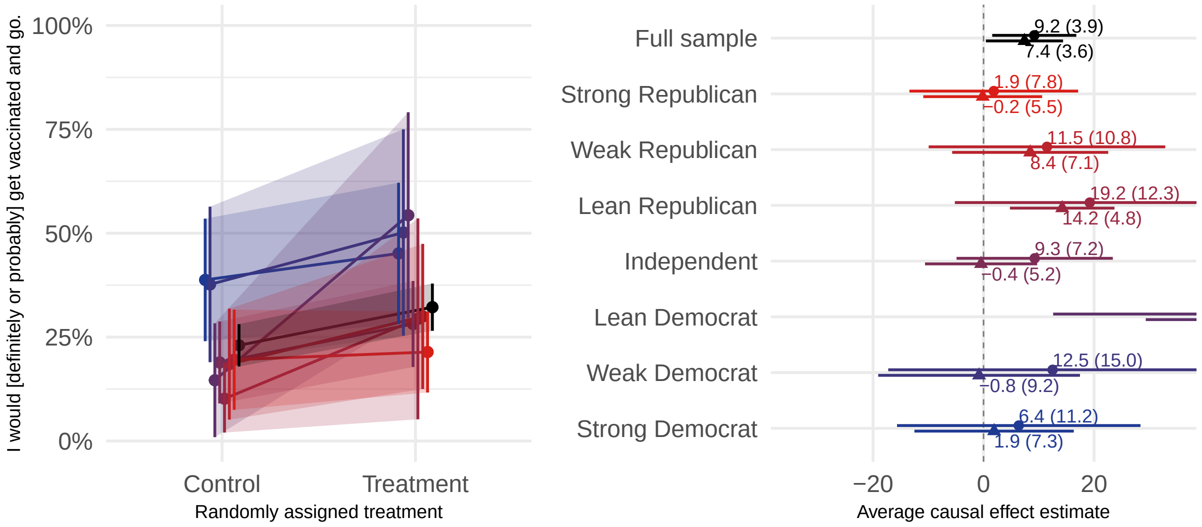

NOTE: Plotting symbols on the right-side panel represent the conditional average treatment effects within each row. Circles show the effects without covariates. Triangles represent the estimates from a model with covariates as discussed earlier. Lines on the left-side panel represent the average effects overall (black line) and for each level of partisanship (red for Republicans and blue for Democrats). Shades of the lines increase with increasing partisan intensity.

Figure S50: Effects of Trip Mandate (Solo) on Intentions to Vaccinate (June 2021, experiment G-2)

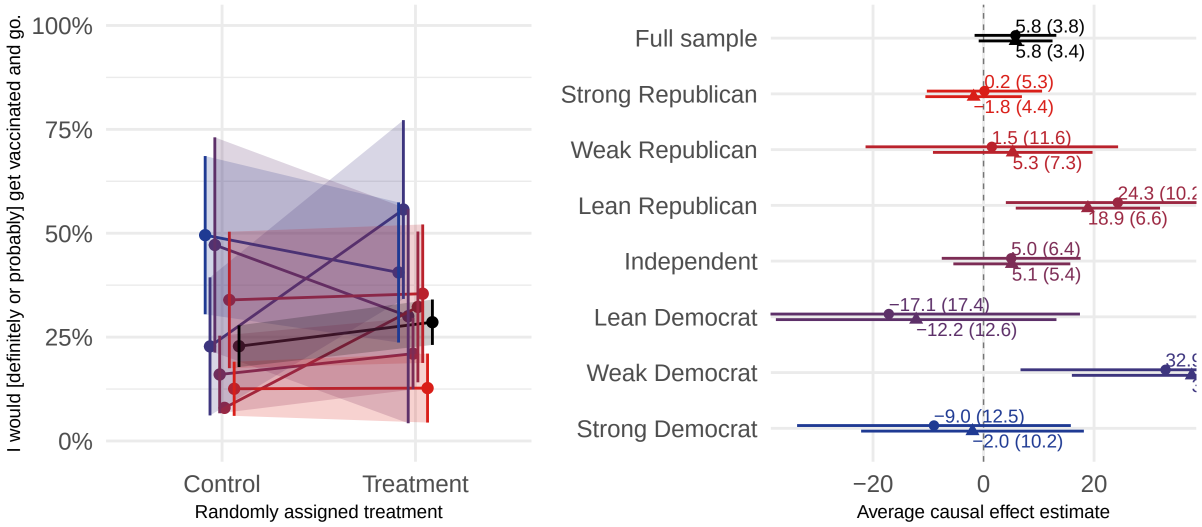

NOTE: Plotting symbols on the right-side panel represent the conditional average treatment effects within each row. Circles show the effects without covariates. Triangles represent the estimates from a model with covariates as discussed earlier. Lines on the left-side panel represent the average effects overall (black line) and for each level of partisanship (red for Republicans and blue for Democrats). Shades of the lines increase with increasing partisan intensity.

Figure S51: Effects of Concert Mandate (Pooled) on Intentions to Vaccinate (June 2021, experiment G-2)

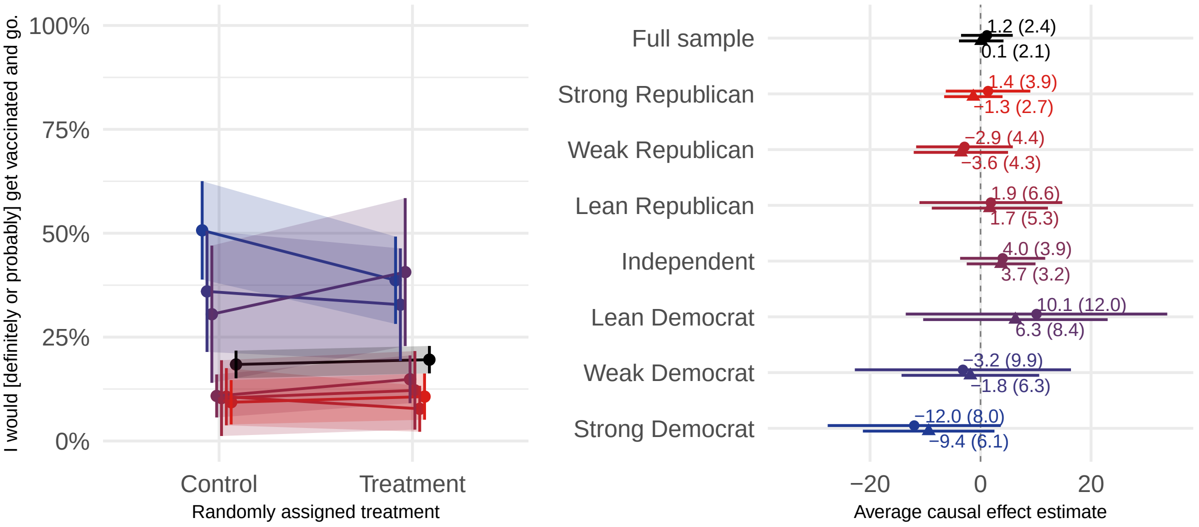

NOTE: Plotting symbols on the right-side panel represent the conditional average treatment effects within each row. Circles show the effects without covariates. Triangles represent the estimates from a model with covariates as discussed earlier. Lines on the left-side panel represent the average effects overall (black line) and for each level of partisanship (red for Republicans and blue for Democrats). Shades of the lines increase with increasing partisan intensity.

Figure S52: Effects of Concert Mandate (Friend) on Intentions to Vaccinate (June 2021, experiment G-2)

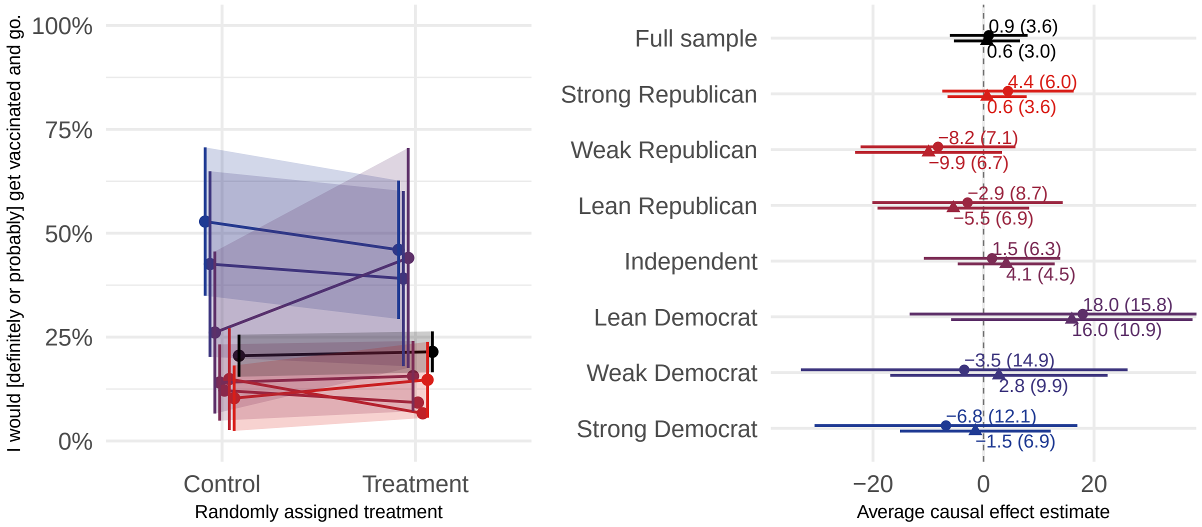

NOTE: Plotting symbols on the right-side panel represent the conditional average treatment effects within each row. Circles show the effects without covariates. Triangles represent the estimates from a model with covariates as discussed earlier. Lines on the left-side panel represent the average effects overall (black line) and for each level of partisanship (red for Republicans and blue for Democrats). Shades of the lines increase with increasing partisan intensity.

Figure S53: Effects of Concert Mandate (Solo) on Intentions to Vaccinate (June 2021, experiment G-2)

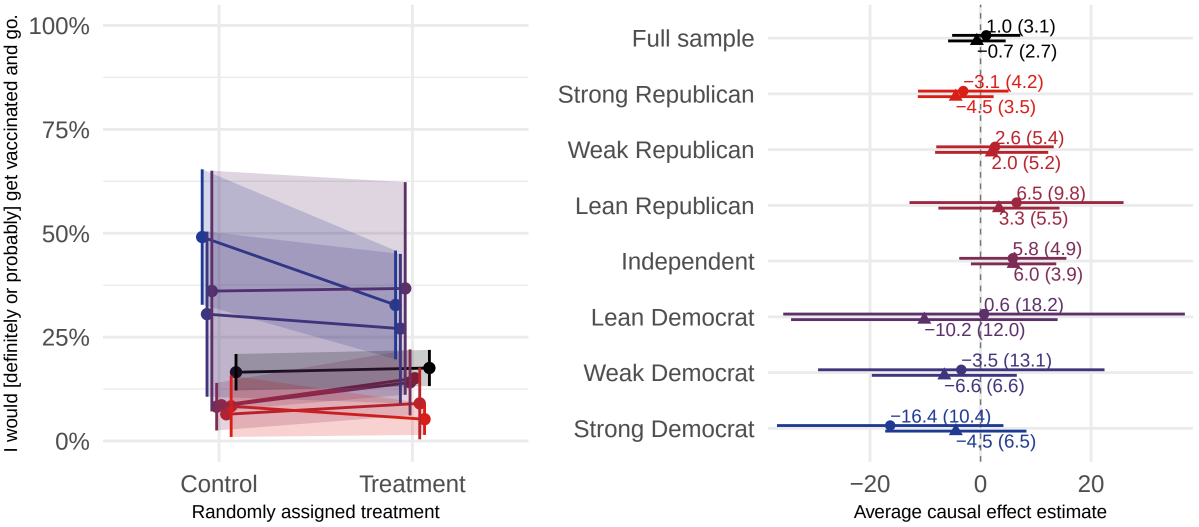

NOTE: Plotting symbols on the right-side panel represent the conditional average treatment effects within each row. Circles show the effects without covariates. Triangles represent the estimates from a model with covariates as discussed earlier. Lines on the left-side panel represent the average effects overall (black line) and for each level of partisanship (red for Republicans and blue for Democrats). Shades of the lines increase with increasing partisan intensity.

Figure S54: Effects of Restaurant Mandate (Pooled) on Intentions to Vaccinate (June 2021, experiment G-2)

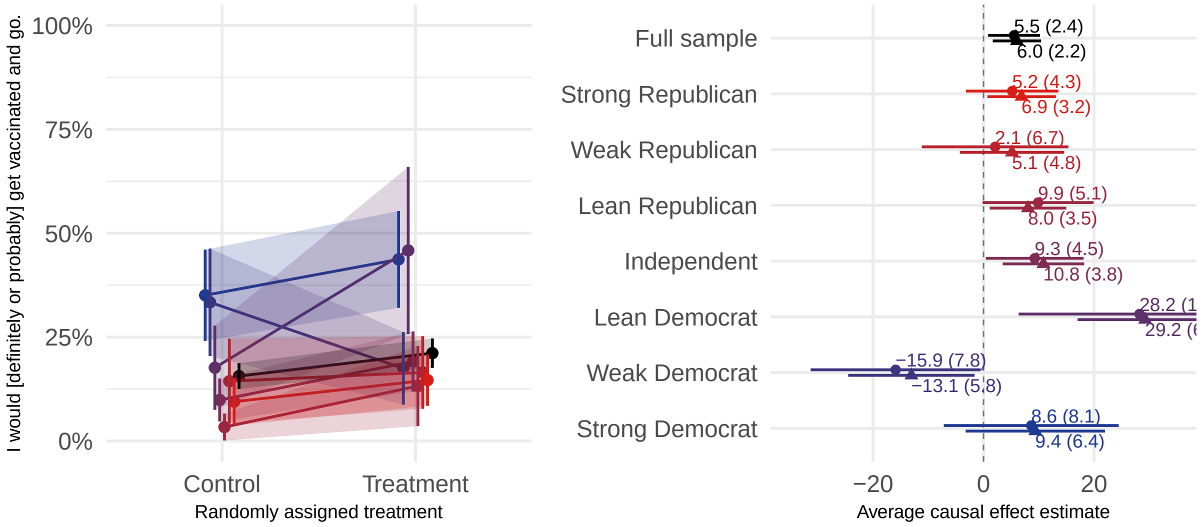

NOTE: Plotting symbols on the right-side panel represent the conditional average treatment effects within each row. Circles show the effects without covariates. Triangles represent the estimates from a model with covariates as discussed earlier. Lines on the left-side panel represent the average effects overall (black line) and for each level of partisanship (red for Republicans and blue for Democrats). Shades of the lines increase with increasing partisan intensity.

Figure S55: Effects of Restaurant Mandate (Friend) on Intentions to Vaccinate (June 2021, experiment G-2)

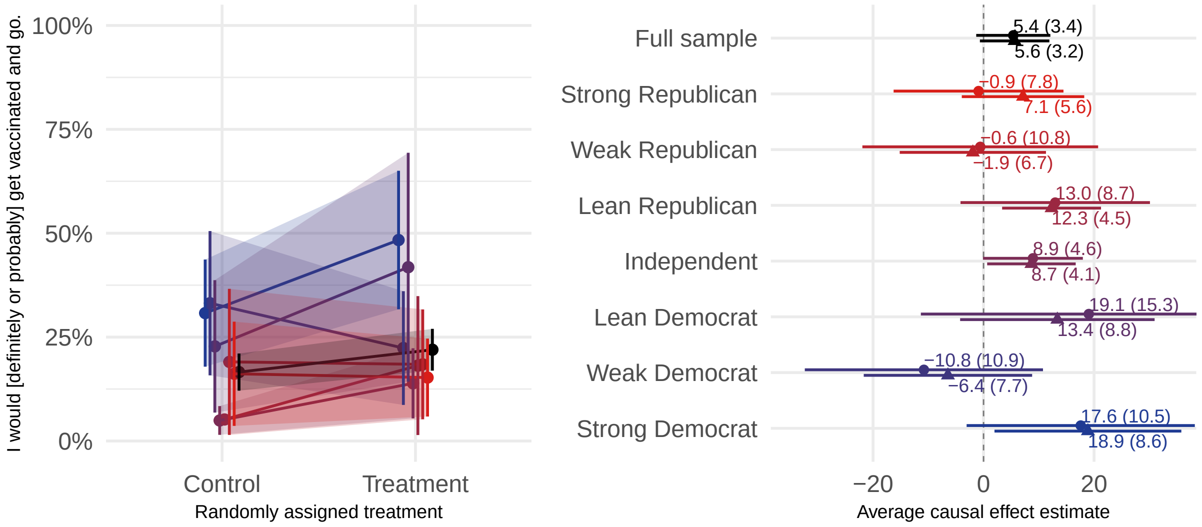

NOTE: Plotting symbols on the right-side panel represent the conditional average treatment effects within each row. Circles show the effects without covariates. Triangles represent the estimates from a model with covariates as discussed earlier. Lines on the left-side panel represent the average effects overall (black line) and for each level of partisanship (red for Republicans and blue for Democrats). Shades of the lines increase with increasing partisan intensity.

Figure S56: Effects of Restaurant Mandate (Solo) on Intentions to Vaccinate (June 2021, experiment G-2)

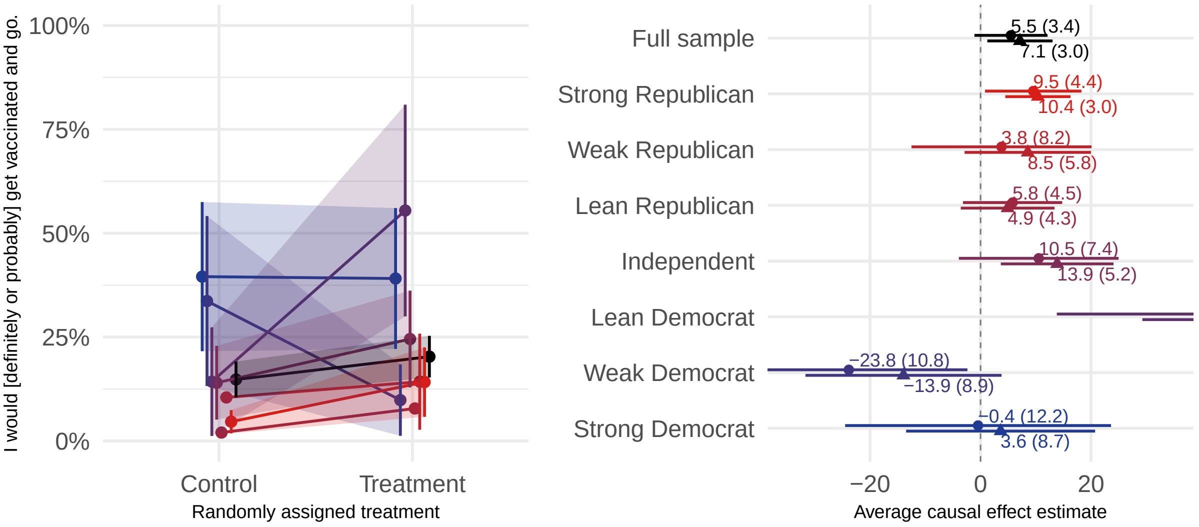

NOTE: Plotting symbols on the right-side panel represent the conditional average treatment effects within each row. Circles show the effects without covariates. Triangles represent the estimates from a model with covariates as discussed earlier. Lines on the left-side panel represent the average effects overall (black line) and for each level of partisanship (red for Republicans and blue for Democrats). Shades of the lines increase with increasing partisan intensity.

Figure S57: Effects of Team Mandate (Pooled) on Intentions to Vaccinate (June 2021, experiment G-2)

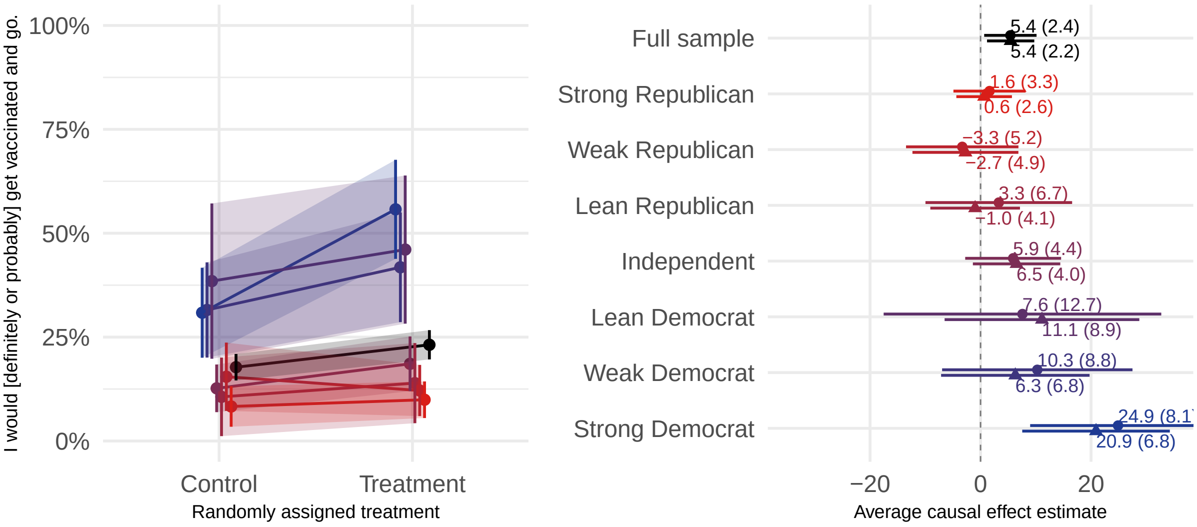

NOTE: Plotting symbols on the right-side panel represent the conditional average treatment effects within each row. Circles show the effects without covariates. Triangles represent the estimates from a model with covariates as discussed earlier. Lines on the left-side panel represent the average effects overall (black line) and for each level of partisanship (red for Republicans and blue for Democrats). Shades of the lines increase with increasing partisan intensity.

Figure S58: Effects of Team Mandate (Friend) on Intentions to Vaccinate (June 2021, experiment G-2)

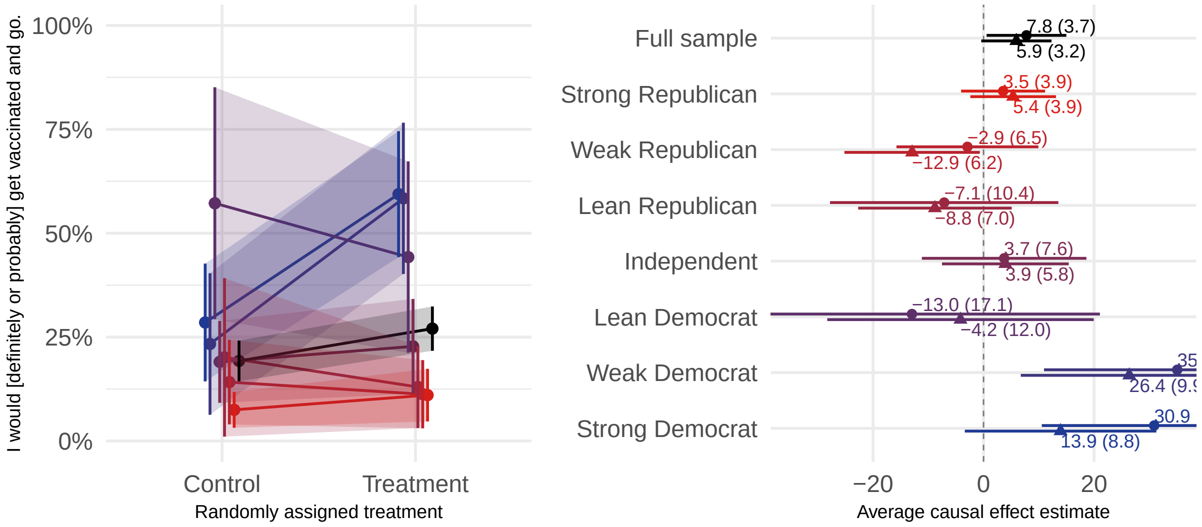

NOTE: Plotting symbols on the right-side panel represent the conditional average treatment effects within each row. Circles show the effects without covariates. Triangles represent the estimates from a model with covariates as discussed earlier. Lines on the left-side panel represent the average effects overall (black line) and for each level of partisanship (red for Republicans and blue for Democrats). Shades of the lines increase with increasing partisan intensity.

Figure S59: Effects of Team Mandate (Solo) on Intentions to Vaccinate (June 2021, experiment G-2)

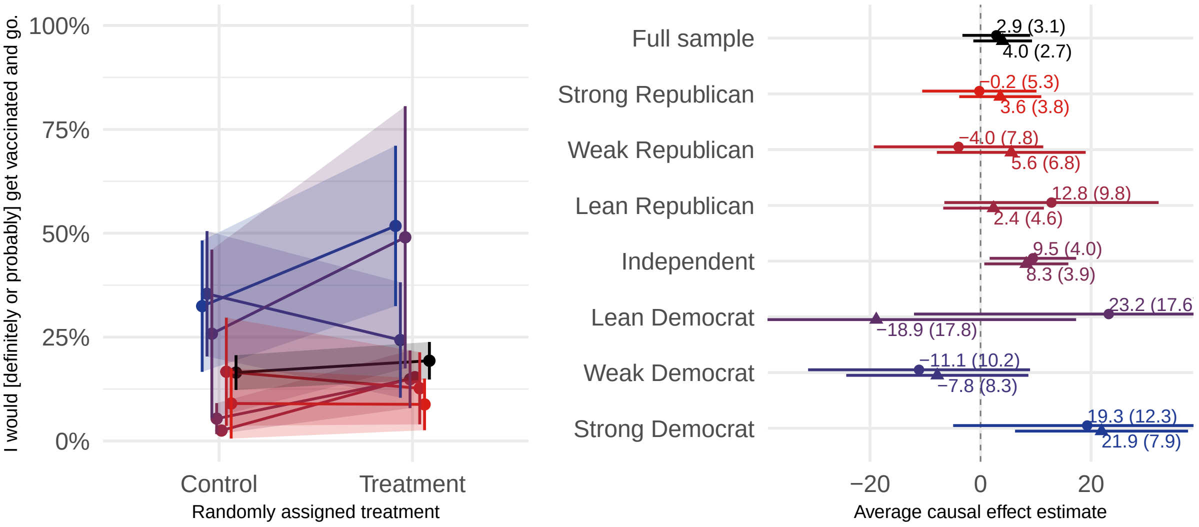

NOTE: Plotting symbols on the right-side panel represent the conditional average treatment effects within each row. Circles show the effects without covariates. Triangles represent the estimates from a model with covariates as discussed earlier. Lines on the left-side panel represent the average effects overall (black line) and for each level of partisanship (red for Republicans and blue for Democrats). Shades of the lines increase with increasing partisan intensity.

E.4 Information Experiments

Figure S60: Effects of Contagiousness Information by Source (Experiment I-1)

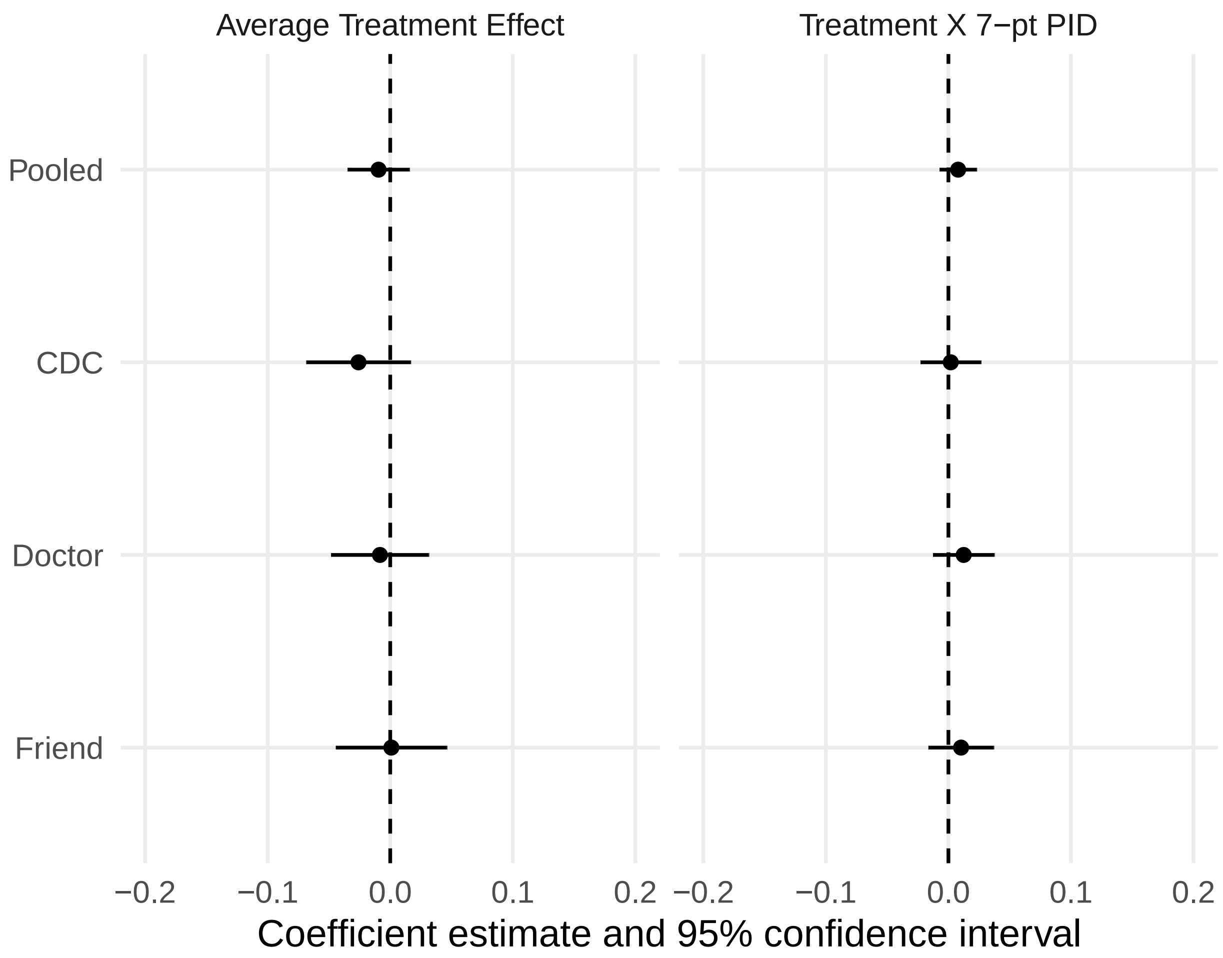

Figure S61: Effects of Source of Contagiousness Information (Experiment I-1)

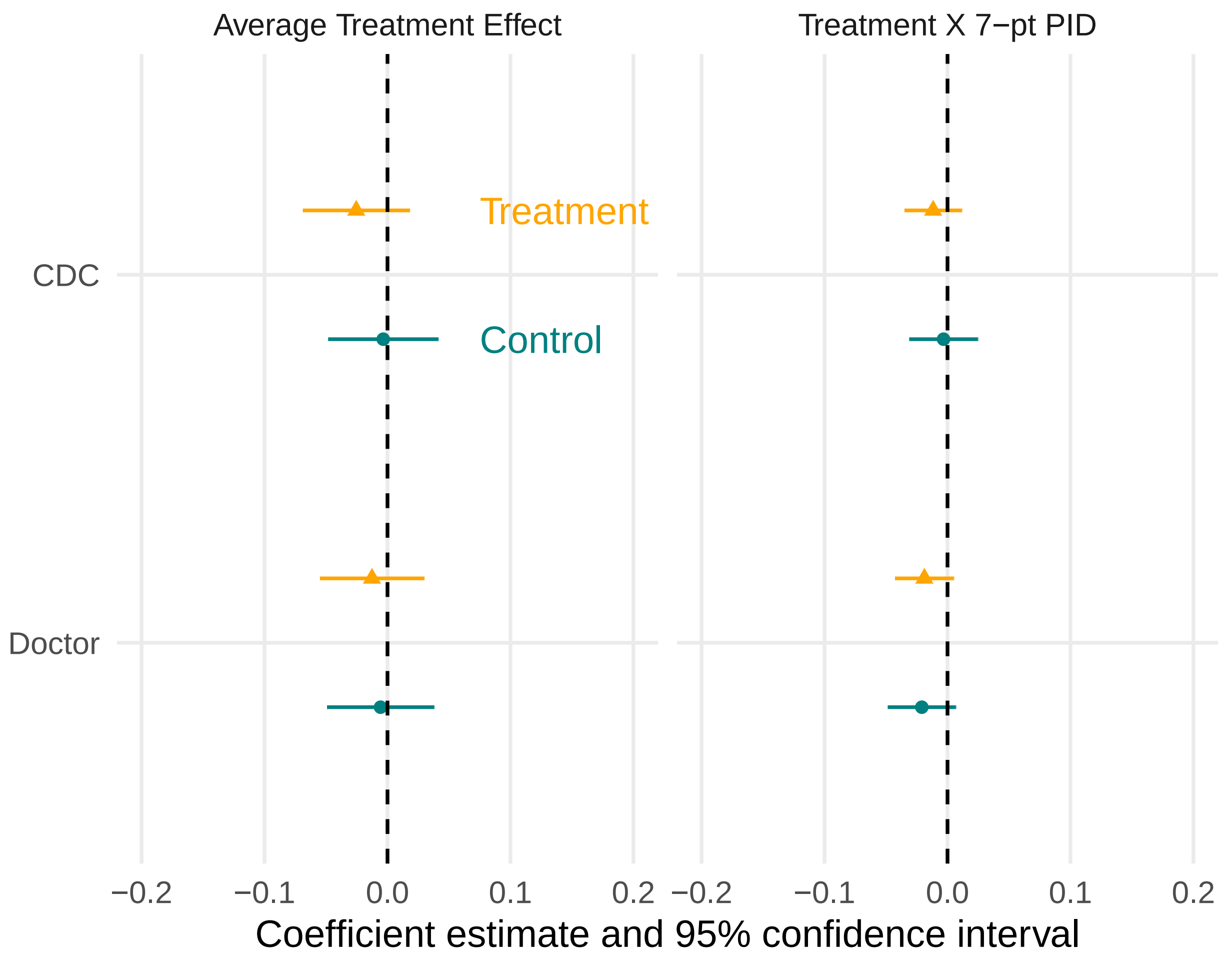

Information Experiments CATEs, Difference-in-CATEs, and Equivalence Tests

Experiment	CATEs		DIC	Equivalence Tests	
-	Dem	GOP	GOP - Dem	5pp	10pp
I-1: Contagiousness Conversation	-0.050 (0.030)	-0.007 (0.016)	0.043 (0.023)	0.371	0.006 *
I-2: Delta Variant Conversation	0.013 (0.033)	0.025 (0.018)	0.012 (0.025)	0.065	0.000 *
I-3: Bivalent Booster Information	0.104 (0.019) *	0.077 (0.022) *	-0.027 (0.031)	0.230	0.009 *
I-4: Bivalent Booster Information (Children)	0.149 (0.041) *	0.235 (0.056) *	0.085 (0.079)	0.671	0.426
I-5: Holiday Surge Information (Children)	0.047 (0.057)	0.044 (0.047)	-0.003 (0.066)	0.236	0.070

F Survey instruments

Endorsement Experiment: October 2020 and March – April 2021

Question Prompt (October 2020):

"If a safe and effective vaccine for COVID-19 were made easily available **through a fast-track approval process** at no cost to everyone in the next several weeks, how likely would you be to get it? Assume the vaccine has the following properties:"

- It has only a few, mild side effects, like stiffness at the injection site.
- It would protect you from getting COVID-19 for at least a year.
- It is endorsed by _________.

Response Options:

- Very likely
- Somewhat likely
- Somewhat unlikely
- Very unlikely

F.1 Activity-Specific Mandates Experiment: June – July 2021

This experiment assessed whether survey respondents would get vaccinated in order to go to a specific activity. Only half of the unvaccinated survey respondents were assigned to these experimental conditions. The experimental conditions varied in two ways:

- **Whether vaccination is required for activity**
  - **Control:** Vaccination is not required for attendance.
  - **Treatment:** Vaccination is required for attendance.
- **What activity is involved**
  - **Arm 1:** Going to a restaurant.
  - **Arm 2:** Going to a team game.
  - **Arm 3:** Going to a concert.
  - **Arm 4:** Traveling on a trip.

Stylized Question Prompt: "Your friend's favorite [ARM: specific activity] is occurring near your town. You know it would be the perfect gift for your friend's birthday and it costs exactly what you had hoped to spend. You want to surprise your friend with this gift. [TREATMENT: 'Because' or 'Even though'] there will be lots of people together, proof of a COVID-19 vaccination

[TREATMENT: 'is required' or 'is NOT required'] to enter the venue. You believe your friend has been vaccinated. Which of the following best describes what you would do in this situation?"

Note: Each prompt, except for the last two sentences, is slightly different for each activity. For example, the "trip" condition mentions travel restrictions. The full descriptions are given below.

- I would definitely get vaccinated and go.
- I would probably get vaccinated and go.
- I would not get vaccinated but still try to go.
- I would probably not get vaccinated and buy something else.
- I would definitely not get vaccinated and buy something else.

Full Question Prompts by Activity:

CONCERT: "Your friend's favorite band is giving a concert near your town. You know it would be the perfect gift for your friend's birthday and it costs exactly what you had hoped to spend. You want to surprise your friend with this gift. ['Because' or 'Even though'] will be lots of people together, proof of a COVID-19 vaccination ['is' or 'is **NOT**'] required to enter the venue.

TEAM: "Your friend's favorite team is playing near your town. You know it would be the perfect gift for your friend's birthday and it costs exactly what you had hoped to spend. You want to surprise your friend with this gift. ['Because' or 'Even though'] there will be lots of people together, proof of a COVID-19 vaccination ['is' or 'is **NOT**'] required to enter the venue."

RESTAURANT: "Your friend's favorite restaurant is finally re-opening. You know going would be the perfect gift for your friend's birthday and the meal will cost exactly what you had hoped to spend. You want to surprise your friend with this gift. ['Because' or 'Even though'] there will be lots of people together, proof of a COVID-19 vaccination ['is' or 'is **NOT**'] required to enter the restaurant. "

TRIP: "You and your friend have always wanted to take a trip together. Travel is affordable right now, and you know a trip would be the perfect gift for your friend's birthday. It would cost exactly what you had hoped to spend. You want to surprise your friend with this gift. ['Because of travel restrictions' or 'Even with travel restrictions'], proof of a COVID-19 vaccination ['is' or 'is **NOT**'] required to take this trip." Employment Mandates: June – July 2021 Question Prompt: "If your employer made a COVID-19 vaccination mandatory to return to work, would you get the vaccine?"

Response Options:

- Yes
- No

F.2 CDC Mask Guidance Experiments

G-1: CDC Mask Guidance 1: Wave 6 Experiment
[CONTROL] Thinking about wearing masks inside of public places, do you think...

- Everyone should continue to do this for a little while longer regardless of vaccination status
- Everyone should stop doing this now regardless of vaccination status
- Vaccinated people don’t need to do this but unvaccinated people do

[TREATMENT] Thinking about wearing masks inside of public places, do you think...

- Everyone should continue to do this for a little while longer regardless of vaccination status
- Everyone should stop doing this now regardless of vaccination status
- Following CDC recommendations, vaccinated people don’t need to do this but unvaccinated people do

G-3: CDC Mask Guidance 2
[CONTROL] Thinking about wearing masks inside of public places, do you think...

- Everyone should continue to do this for a little while longer regardless of vaccination status
- Everyone should stop doing this now regardless of vaccination status
- Vaccinated people don’t need to do this but unvaccinated people do

[TREATMENT] Thinking about wearing masks inside of public places, do you think...

- Following CDC recommendations, everyone should continue to do this for a little while longer regardless of vaccination status
- Everyone should stop doing this now regardless of vaccination status
- Vaccinated people don’t need to do this but unvaccinated people do

F.3 I-1: Contagiousness Conversation

[TREATMENT A] Imagine a friend mentions that over 90% of Americans in the hospital right now due to COVID-19 are unvaccinated. Your friend also says that the Delta variant of the virus is more than twice as contagious as the original virus and that it is as contagious as the Chicken Pox. Would this information make you more or less likely to get vaccinated?

- More likely
- Less likely
- It wouldn’t affect my decision

[TREATMENT B] Imagine a friend mentions that lots of unvaccinated people are being hospitalized for COVID-19 right now. Imagine your friend also says that it seems like the virus has become more contagious. Would this information make you more or less likely to get vaccinated?

- More likely
- Less likely
- It wouldn't affect my decision

[TREATMENT C] Imagine your doctor mentions that over 90% of Americans in the hospital right now due to COVID-19 are unvaccinated. Your doctor also says that the Delta variant of the virus is more than twice as contagious as the original virus and that it is as contagious as the Chicken Pox. Would this information make you more or less likely to get vaccinated?

- More likely
- Less likely
- It wouldn't affect my decision

[TREATMENT D] Imagine your doctor mentions that lots of unvaccinated people are being hospitalized for COVID-19 right now. Imagine your doctor also says that it seems like the virus has become more contagious. Would this information make you more or less likely to get vaccinated?

- More likely
- Less likely
- It wouldn't affect my decision

[TREATMENT E] Imagine the CDC reports that over 90% of Americans in the hospital right now due to COVID-19 are unvaccinated. Imagine it also reports that the Delta variant of the virus is more than twice as contagious as the original virus and that it is as contagious as the Chicken Pox. Would this information make you more or less likely to get vaccinated?

- More likely
- Less likely
- It wouldn't affect my decision

[TREATMENT F] Imagine the CDC reports that lots of unvaccinated people are being hospitalized for COVID-19 right now. Imagine it also reports that the virus has become more contagious. Would this information make you more or less likely to get vaccinated?

- More likely
- Less likely
- It wouldn't affect my decision

F.4 I-2: Delta Variant Conversation

[CONTROL] Imagine you're having a conversation with your doctor about the way businesses in your neighborhood are reacting to the spread of the highly contagious Delta variant of the virus.
Your doctor listens to your concerns about the vaccine and understands your worry but emphasizes the increased contagiousness of Delta, saying the risks are minimal. Your doctor urges you to get a vaccine shot. Would you...

- Let your doctor vaccinate you in the office that same day
- Make an appointment to get vaccinated later and keep it
- Make an appointment to get vaccinated later and cancel it
- Decline to be vaccinated

[TREATMENT] Imagine you're having a conversation with your doctor about the way businesses in your neighborhood are reacting to the state of the virus.
Your doctor listens to your concerns about the vaccine and understands your worry but emphasizes the risks are minimal. Your doctor urges you to get a vaccine shot. Would you...

- Let the doctor vaccinate you in the office that same day
- Make an appointment to get vaccinated later and keep it
- Make an appointment to get vaccinated later and cancel it
- Decline to be vaccinated

F.5 Bivalent Booster Experiments

F.5.1 Step 1: Measure Bivalent Booster Uptake

On August 31, 2022, the Food and Drug Administration authorized the use of an updated COVID-19 vaccine and booster for adults. On October 12, 2022, it was approved for children age five and up. These "bivalent" vaccines and boosters were approved for adults earlier in 2022 and protect against the original COVID strain and the more recent Omicron variants of the virus.

Have you received a dose of a "bivalent" COVID-19 booster since August 31, 2022?

- Yes
- No
- Not sure (I got a booster shot since August 31, but don't know if it was the new one or the original)

Have any of the eligible children in your household received a dose of the "bivalent" COVID-19 booster since October 12, 2022?

- Yes

- No
- Not sure (I got them a booster shot since October 12, but don't know if it was the new one or the original)

F.5.2 Step 2: Deliver Information About Potential Surge

Start of Block: BIVALENT EXPERIMENT STEP 2

[CONTROL] How likely are you to get the "bivalent" COVID-19 booster this year?

- I will definitely get it
- I am very likely to get it
- I am somewhat likely to get it
- I am not very likely to get it
- I will not get it

[TREATMENT: VACCINATED INDIVIDUALS] Doctors and researchers are warning Americans that another COVID-19 surge will occur this Winter though they are not yet sure how it will compare to last year's Omicron surge. The CDC reports that vaccines and boosters are the best way to protect yourself and your family against severe COVID-19 disease, potential long-term complications, and death.
Knowing that another COVID-19 surge is likely between November and January, how likely are you to get the "bivalent" COVID-19 booster this year?

- I will definitely get it
- I am very likely to get it
- I am somewhat likely to get it
- I am not very likely to get it
- I will not get it

[TREATMENT: UNVACCINATED INDIVIDUALS] Doctors and researchers are warning Americans that another COVID-19 surge will occur this Winter though they are not yet sure how it will compare to last year's Omicron surge. The CDC reports that vaccines and boosters are the best way to protect yourself and your family against severe COVID-19 disease (and potential long-term complications) and death.

Given that another Winter COVID-19 surge is coming before the end of the year, which of the following best describes what you will do?

- I will definitely get a vaccine
- I will probably get a vaccine

- I am unsure about getting vaccinated
- I will probably NOT get vaccinated
- I will definitely NOT get vaccinated

F.5.3 Step 3: Measure Intentions Towards Childrens' Vaccinations

[UNBOOSTED CHILDREN] How likely are you to get the children living in your household the "bivalent" COVID-19 booster this year?

- I will definitely do it
- I am very likely to do it
- I am somewhat likely to do it
- I am not very likely to do it
- I will not do it

[UNVACCINATED CHILDREN] How likely are you to vaccinate the eligible, unvaccinated children in your household for COVID-19?

- Extremely likely
- Very likely
- Somewhat likely
- Not at all likely
- Not sure

F.6 Measuring Party Identification

Generally speaking, do you think of yourself as a ...?

- Democrat
- Republican
- Independent
- Something else ___________________

[IF DEMOCRAT] Do you think of yourself as a...

- Strong Democrat
- Not very strong Democrat

[IF Republican] Do you think of yourself as a...

- Strong Republican
- Not very strong Republican

[IF INDEPENDENT OR SOMETHING ELSE] Do you think of yourself as closer to the Republican Party or to the Democratic Party?

- Closer to the Republican Party
- Closer to the Democratic Party
- Neither

F.7 Measuring Vaccination Status

F.7.1 Vaccine Intent: October 2020 and December 2020

Question Prompt: "If a vaccine for COVID-19 were approved by the FDA, free to everyone, and easily available would you get it?"
Response Options:

- Yes
- No
- Unsure

F.7.2 Vaccine Uptake: March - April 2021, June - July 2021, and September – October 2021

Question Prompt: "How many doses of a COVID-19 vaccine have you received to date, if any?"
Response Options:

- None
- 1 out of 1
- 1 out of 2
- 2 out of 2

F.7.3 Vaccine Uptake: September 2022

Question Prompt: "How many doses of a COVID-19 vaccine have you received to date, **NOT INCLUDING BOOSTER SHOTS?**"
Response Options:

- None
- 1 out of 1
- 1 out of 2
- 2 out of 2
- More than 2 (NOT INCLUDING A BOOSTER)

G Estimates from all studies

Figure S62: Estimates from all studies

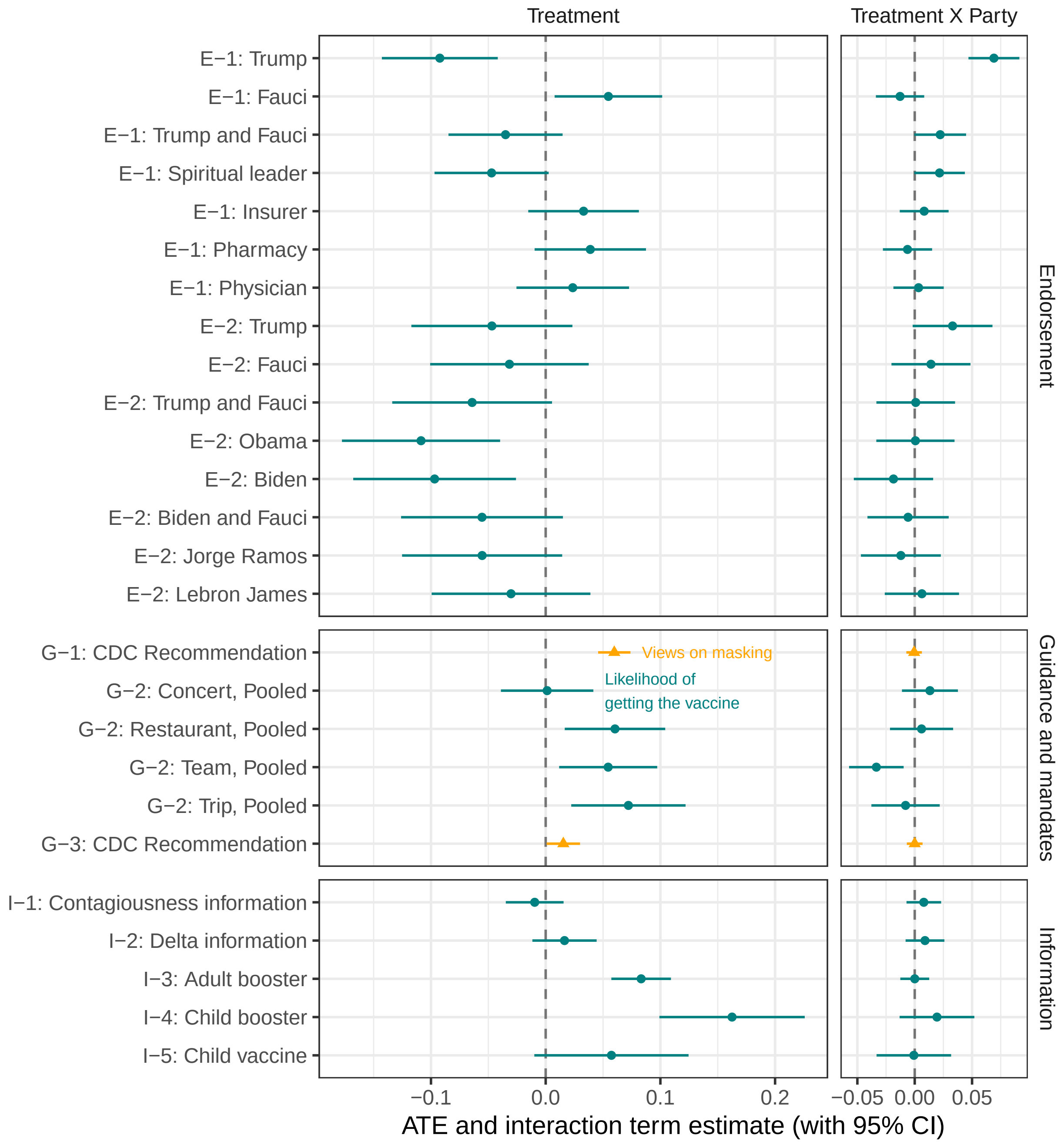

Supplement: pgaf185_Supplementary_Data [file pgaf185_supplementary_data.pdf]
